# Visible‐Light‐Mediated Lewis Acid‐Catalyzed Diradical Hydrogen Atom Transfer Reaction of Enamides

**DOI:** 10.1002/advs.76846

**Published:** 2026-08-06

**Authors:** Yao Huang, Baoyu Yang, Xiang Zhou, Israel Fernández, Kexue Xia, Yang Xiong

**Affiliations:** ^1^ Chongqing Key Laboratory of Natural Product Synthesis and Drug Research Innovative Drug Research Center School of Pharmaceutical Sciences Chongqing University Chongqing People's Republic of China; ^2^ Departamento de Química Orgánica and Centro de Innovación en Química Avanzada (ORFEO‐CINQA) Facultad de Ciencias Químicas Universidad Complutense de Madrid Madrid Spain; ^3^ College of Traditional Chinese Medicine Chongqing Medical University Chongqing People's Republic of China

**Keywords:** diradical hydrogen atom transfer reaction, enamides, Lewis acid‐catalyzed, visible‐light‐mediated, β‐lactams

## Abstract

β‐Lactams are of great importance in organic chemistry and medicinal science due to their essential biologically active and unique architectures. Herein, the facile photochemical synthesis of highly functionalized β‐lactams derivatives from easily accessible enamides, enabled by a Lewis acid‐catalyzed diradical hydrogen atom transfer (DHAT), is described. Notably, trisubstituted enamides with tertiary C(sp^3^)−H bonds can efficiently deliver adjacent all‐carbon quaternary β‐lactams. This effective protocol is compatible with a variety of functional groups and can be applied to the modification of bioactive molecules. Based on mechanistic evidences and computational studies, it is suggested that Lewis acid catalysis can lower the energy of the triplet excited state of enamides and modulate the reactivity of the readily formed 1,2‐diradical intermediate to unlock a carbon‐to‐carbon 1,5‐DHAT and consequent cyclization of the 1,4‐diradical intermediate that allows the functionalization of C(sp^3^)−H bonds to construct a variety of novel β‐lactams. This distinctive activation mode of Lewis acid catalysis paves a new way of DHAT reactions for the transformation of C(sp^3^)−H bonds.

## Graphical abstract

The Lewis acid catalysis was crucial for the success of the DHAT reaction of enamides by lowering the energy of the triplet excited state and modulating the reactivity of the formed diradical intermediate in a carbon‐to‐carbon 1,5‐DHAT and consequent cyclization that allows the functionalization of C(sp^3^)−H bonds to construct a variety of novel β‐lactams.

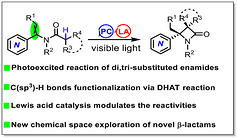

## 1 Introduction

**FIGURE 1:**
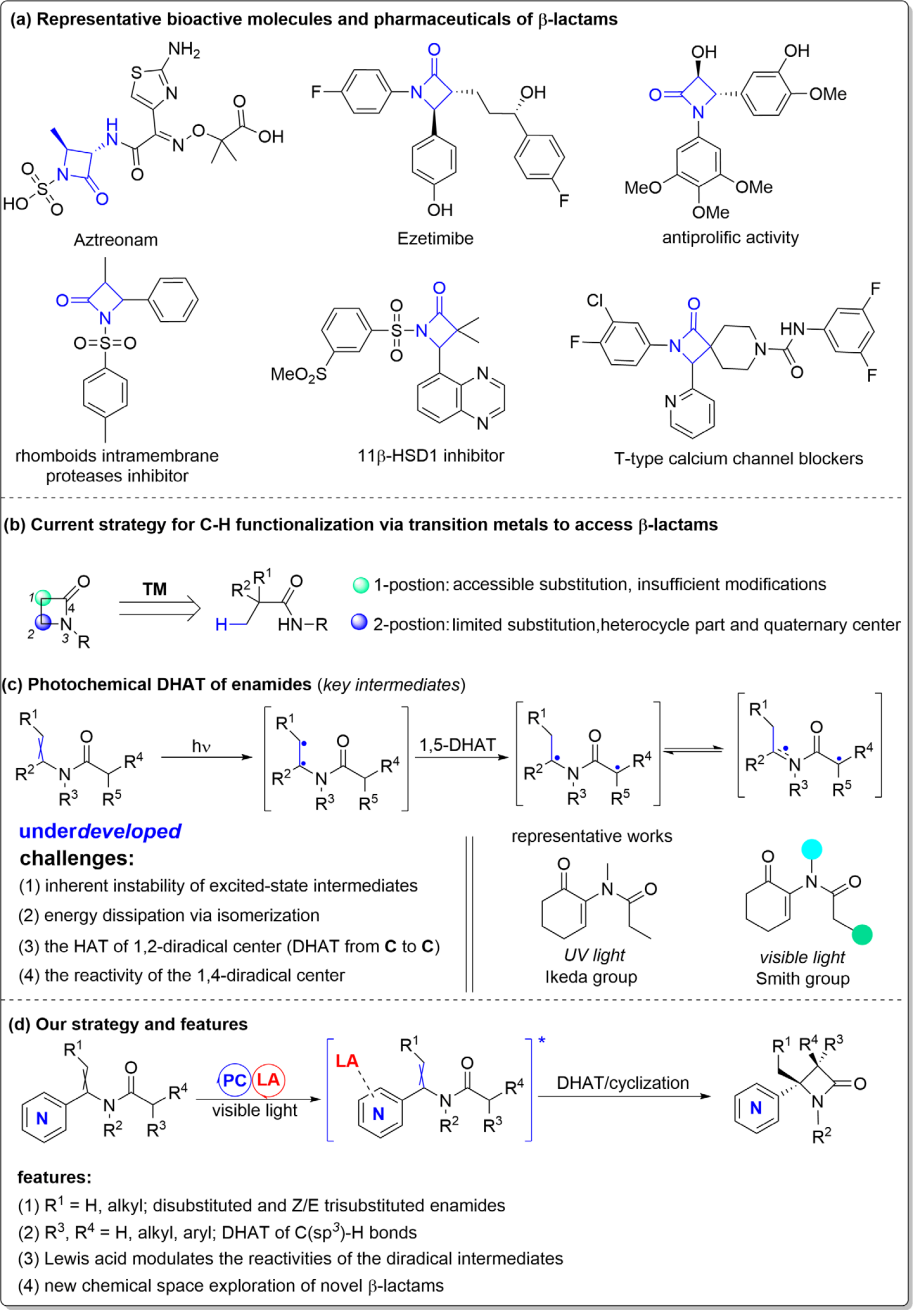
Background and photochemical DHAT reactions of enamides. (a) Pharmaceutically examples of β‐lactams; (b) Retrosynthesis of β‐lactams via C−H functionalization using transition metals; (c) Photochemical DHAT of enamides; (d) Our design.

β‐Lactams have a crucial role in the fields of organic chemistry and medicinal science due to their prevalence in essential natural products, biologically active molecules, and pharmaceuticals [1, 2]. It is widely accepted that β‐lactams are extremely important architectures in a variety of antibiotics, such as penicillins, cephalosporins, and carbapenems (Figure 1a) [3, 4]. In addition, β‐lactams were also extensively used as versatile synthetic intermediates in organic synthesis [5, 6, 7]. In this context, innovative methodologies to obtain β‐lactams with different physical properties and unexplored chemical space are of great importance and highly desirable. It was noted that the Staudinger ketene‐imine [2+2] cycloaddition is regarded as one of the most fundamental and versatile methods for the synthesis of such derivatives; however, the generation of substituted ketenes in the reaction limited its applications in some conditions [8, 9]. Despite the great interests and recent advances in the synthesis of these compounds, an alternative strategy involving C−H transformations represents an atom‐efficient route to access this motif in a straightforward manner (Figure 1b) [10, 11, 12, 13]. However, this strategy is likely not suitable for tertiary C(sp^3^)−H transformations and heterocycle‐containing β‐lactams. Considering the distinctive structural and bioactive properties imparted by heterocycles and quaternary carbons in pharmaceuticals [14, 15], the development of efficient strategies to incorporate these vital motifs into β‐lactam scaffolds is necessary to increase their potential in current drug design and therapy. Accordingly, the pursuit of modular and practical synthetic strategies toward highly functionalized β‐lactams remains an important issue to address.

On the other hand, enamide serves as a crucial synthetic building block for the synthesis of various nitrogen‐containing compounds [16]. Despite that, the excited‐state characteristics of enamides have significantly hampered the development of their photochemical applications [17]. In fact, the photochemical reactivity of excited enamides is significantly restricted by their ππ* character and the inherent instability of excited‐state intermediates due to the non‐ bonding electron pair on nitrogen [18]. Moreover, the excited isomerization greatly hampers the photochemical reactions of these species. In recent years, with the great renaissance and progress of energy transfer reactions [19, 20, 21, 22, 23, 24], substantial research efforts have been directed toward expanding the boundaries of their reactivity via diradical intermediates.

However, in contrast to photochemical [2+2] reactions [25, 26], the development of other photochemical transformations of excited enamides is still in its early stages, especially diradical hydrogen atom transfer (DHAT) reactions [27]. In fact, the DHAT reactions featuring diradical species derived from excited states, which exhibit unique reactivity, can provide alternative reaction pathways for the transformation of C(sp^3^)−H bonds [28]. In particular, the DHAT and subsequent cyclization of photo‐excited enamides could offer a facile way to realize diverse functionalization of C(sp^3^)−H bonds to access various β‐lactams for exploring new chemical space. While some studies have reported transformations along this line for the construction of β‐lactams, strategies for modulating diradical reactivity and expanding the chemical space remain at a nascent stage [29, 30, 31, 32, 33, 34, 35, 36, 37, 38]. Moreover, several elegant works suggested that upon UV or visible light irradiation, the excited state of enamides can undergo a 1,5‐DHAT via a 1,2‐diradical intermediate to form a new 1,4‐diradical intermediate. However, subsequent intramolecular cyclization is critically governed by the substrate nature due to the lack of precise control of the reactivity and relative stability of the diradical [30, 39]. It is assumed that the increased ππ* character, inherent instability of the excited‐state intermediates, and the reactivity of the involved two diradical intermediates in the processes are the crucial issues to be addressed (Figure 1c). Meanwhile, photochemical C(sp^3^)–H functionalization for the construction of quaternary carbon centers remains challenging because of the high steric hindrance involved. Nevertheless, the development of a visible light‐mediated methodology can greatly expand the scope and generality of this process and pave a practical way for the divergent synthesis of multi‐substituted β‐lactams via C(sp^3^)−H bond functionalization.

Moreover, it is widely accepted that Lewis acids can change the reactivity of excited‐state organic molecules [40, 41, 42, 43, 44, 45, 46]. Recently, Glorius, Brown, You, Feng, and other groups have found that Lewis acids play a vital role in photochemical dearomative cycloaddition reactions [47, 48, 49, 50]. However, to the best of our knowledge, the use of a Lewis acid catalyst to modulate diradical reactivity of excited enamides in DHAT reactions has not been previously reported. Based on our previous studies involving DHAT reactions [51, 52, 53, 54], we hypothesized that, upon a strategic combination of photosensitizer and Lewis acid, it would be possible to modulate the reactivity of the diradical intermediates in the process to unlock the triplet reactivity of enamides for an unprecedented DHAT/cyclization to access highly functionalized β‐lactams. Meanwhile, a flexible coordination with the substrates may allow this process to be catalyzed by a Lewis acid. This hypothesis would enable precise control over diradical reactivity, thereby offering a new concept to advance the general approach for such DHAT reactions (Figure 1d). Herein, we have studied the visible light‐mediated reaction of excited enamides for a Lewis acid‐catalyzed DHAT/cyclization. It is found that various C(sp^3^)−H bonds can be functionalized to provide an effective route to a variety of β‐lactams skeletons. Notably, trisubstituted *Z*/*E* enamides with tertiary C(sp^3^)−H bonds can efficiently deliver adjacent all‐carbon quaternary β‐lactams, unlocking a range of opportunities in biological applications. Mechanistic evidences and computational studies suggest that the Lewis acid catalyst indeed modulates the reactivity of the carbon diradical intermediates to unlock a carbon‐to‐carbon DHAT and its subsequent cyclization that allows the functionalization of C(sp^3^)−H bonds to construct a variety of β‐lactams.

## 2 Results and Discussion

### 2.1 Reaction Design and Development

In light of above considerations, we performed the optimization of the DHAT/cyclization reaction with *N*‐benzyl‐*N*‐(1‐(pyridin‐2‐yl)vinyl)‐cyclohex‐ane‐carboxamide **a1** (Table 1, see Figure S1 for the synthesis method) [51] This model substrate features a cyclohexyl group at the α‐position to the carbonyl group for a possible DHAT and subsequent cyclization to form a β‐lactam with adjacent all‐carbon quaternary centers and the pyridine moiety the coordination site with the Lewis acid. We attempted to make a judicious choice of iridium triplet sensitizers and Lewis acids to get the desired product **b1**. To our delight, when [Ir(dFppy)_2_dtbbpy]PF_6_ and Sc(OTf)_3_ were used under visible light irradiation, product **b1** was obtained (entry 1, Table 1). Various Lewis acids and Brønsted acids were also evaluated (entries 2–5, Table 1), and Zn(OTf)_2_ was found to give the product in the highest yield. Moreover, through a solvent screening process, DCM was identified as the solvent giving the highest yield (entries 6–8, Table 1). Furthermore, other photosensitizers were also tested but performed less efficiently (entries 9–11, Table 1). Lastly, several control experiments confirmed that the reaction requires the presence of the Lewis acid, the photocatalyst, and light (entries 12–14, Table 1).

**TABLE 1:** Reaction development and optimization.

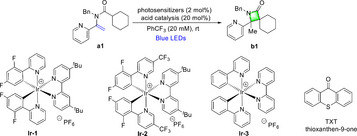				
Entry ^a^	sens	Solvent	Acids	Yield ^b^ (%)
1	**Ir‐1**	PhCF_3_	Sc(OTf)_3_	65
2	**Ir‐1**	PhCF_3_	Y(OTf)_3_	60
3	**Ir**‐**1**	PhCF_3_	Zn(OTf)_2_	72
4	**Ir‐1**	PhCF_3_	Mg(OTf)_2_	37
5	**Ir‐1**	PhCF_3_	TsOH	40
6	**Ir‐1**	CH_2_Cl_2_	Zn(OTf)_2_	91/87 ^c^
7	**Ir‐1**	CH_3_CN	Zn(OTf)_2_	47
8	**Ir‐1**	THF	Zn(OTf)_2_	35
9 ^d^	TXT	CH_2_Cl_2_	Zn(OTf)_2_	58
10	**Ir‐2**	CH_2_Cl_2_	Zn(OTf)_2_	6
11	**Ir‐3**	CH_2_Cl_2_	Zn(OTf)_2_	70
12	**Ir‐1**	CH_2_Cl_2_	—	22
13	—	CH_2_Cl_2_	Zn(OTf)_2_	—
14 ^e^	**Ir‐1**	CH_2_Cl_2_	Zn(OTf)_2_	—

^a^ **a1** (0.2 mmol), photosensitizers (2 mol%), acid catalyst (20 mol%) in solvent (10 mL) under Ar, rt, blue LEDs λ_max_ = 427 nm, 24 h.

^b^ The yield was determined by ^1^H‐NMR using CH_2_Br_2_ as the internal standard.

^c^ Isolated yield.

^d^ blue LEDs λ_max_ = 420 nm.

^e^ Without light.

### 2.2 Reaction Scope

**FIGURE 2:**
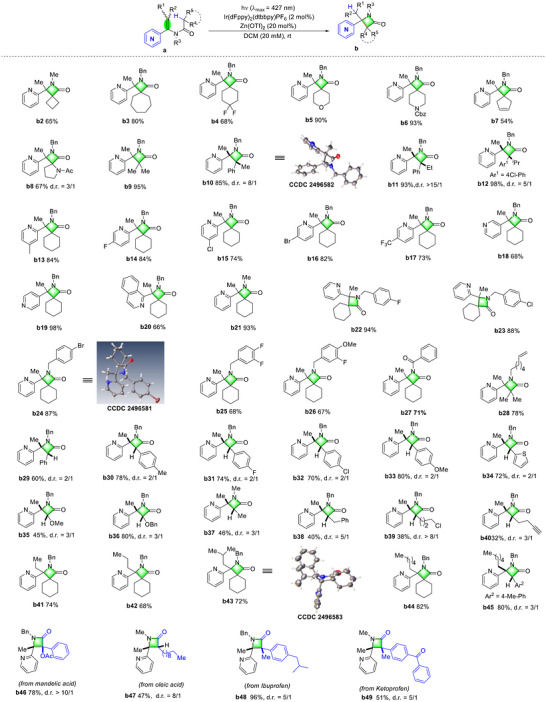
The substrate scope. Reaction conditions: **a** (0.2 mmol), **Ir‐1** (2 mol%), Zn(OTf)_2_ (20 mol%) in DCM (10 mL) under Ar, rt, blue LEDs, λ_max_ = 427 nm, 24 h, for **b10**‐**b12** (reaction time *t* = 12 h), isolated yield, diastereomeric ratio (d.r.) was determined by crude ^1^H NMR integration.

By using the optimized conditions (entry 6, Table 1), the scope of the reaction was then explored with a variety of enamides (Figure 2 and Figure S2). Initially, the transformations of tertiary C(sp^3^)−H bonds at the α‐position of the carbonyl group in enamides **a2**‐**a8** were investigated. It was found that the systematic variation of cyclic substituents, ranging from four‐membered to seven‐membered rings, can access diverse spiro‐β‐lactams (**b2**‐**b8**) with continuous all‐carbon quaternary centres in good to excellent yields, being tolerant to functional groups such as fluoro, alkoxy, alkene, and amide. In particular, spiro‐β‐lactam **b8** has a promising potential for application in chemical space exploration of Zelquistinel for NMDA receptor modulators [55]. Meanwhile, enamides **a9**‐**a12** with acyclic substituents also proceeded efficiently to afford **b9**‐**b12**. The assignment of the product **b10** was corroborated by single‐crystal x‐ray diffraction (CCDC2496582). It was found that the major diastereoisomer displays the phenyl group *cis* relative to the pyridine moiety. Notably, in the absence of the Lewis acid, the dr value of **b10** was only 3:1, indicating that the addition of the Lewis acid modulates the reactivity of the reaction. Moreover, different substituents such as fluoro, chloro, bromo, and trifluoromethyl on the pyridine moiety can also undergo this transformation, thus delivering the corresponding products **b13**‐**b17** in good yields. Furthermore, other *N*‐heteroaromatic units could also be successfully employed, affording the desired products **b18**‐**b20**. In addition, the reaction is also compatible with a variety of *N*‐substituted substrates, including alkyl, benzyl, benzoyl, and alkenoyl groups, leading to the DHAT/cyclization products **b21‐b28**, enabling subsequent diverse transformations of β‐lactams. Then, using this method, the secondary C(sp^3^)−H bonds with different substituents at the α‐position of the carbonyl group in the enamides were also effectively transformed into the corresponding β‐lactams **b29**‐**b40** in 32%–80% yields and 2/1 to > 8/1 d.r. values 1. The reaction can tolerate a wide range of functional groups such as fluoro (**b31**), chloro (**b32**, **b39**), ether (**b33**, **b35**, **b36)**, thiophenyl (**b34**), and alkyne(**b40**). Importantly, enamides **a37**‐**a40**, featuring stronger secondary C(sp^3^)−H bonds (according to Bond Dissociation Energies, BDEs) [56], can also attain the β‐lactams **b37**‐**b40**, albeit in lower yields. In sharp contrast, the primary C(sp^3^)−H bond substrates cannot work in the reaction, likely due to the relative reactivity of the diradical intermediates. Next, trisubstituted enamides, which are challenging substrates due to undesired *E*/*Z* isomerization [57] or a higher triplet energy [58]. were also employed. To our delight, it was found that such enamides efficiently produced the desired products (**b41**‐**b45**) in good yields, which highlights the distinctive reactivity imparted by Lewis acid catalysis in DHAT reactions. Lastly, to further showcase the practical utility of this strategy, molecules containing both biological and drug motifs were also investigated under the optimized conditions. Thus, the mandelic and oleic acids can be efficiently incorporated into the β‐lactam (**b46** and **b47**) with satisfactory yield and d.r. via this method, thereby significantly expanding the accessible chemical space. The Ibuprofen and Ketoprofen derivatives, to our delight, were found to be effective substrates in the reactions, resulting in the corresponding β‐lactams in good yields (**b48**, 96 %; **b49**, 51 %) and diastereomeric ratio (5/1).

### 2.3 Mechanistic Studies

**FIGURE 3:**
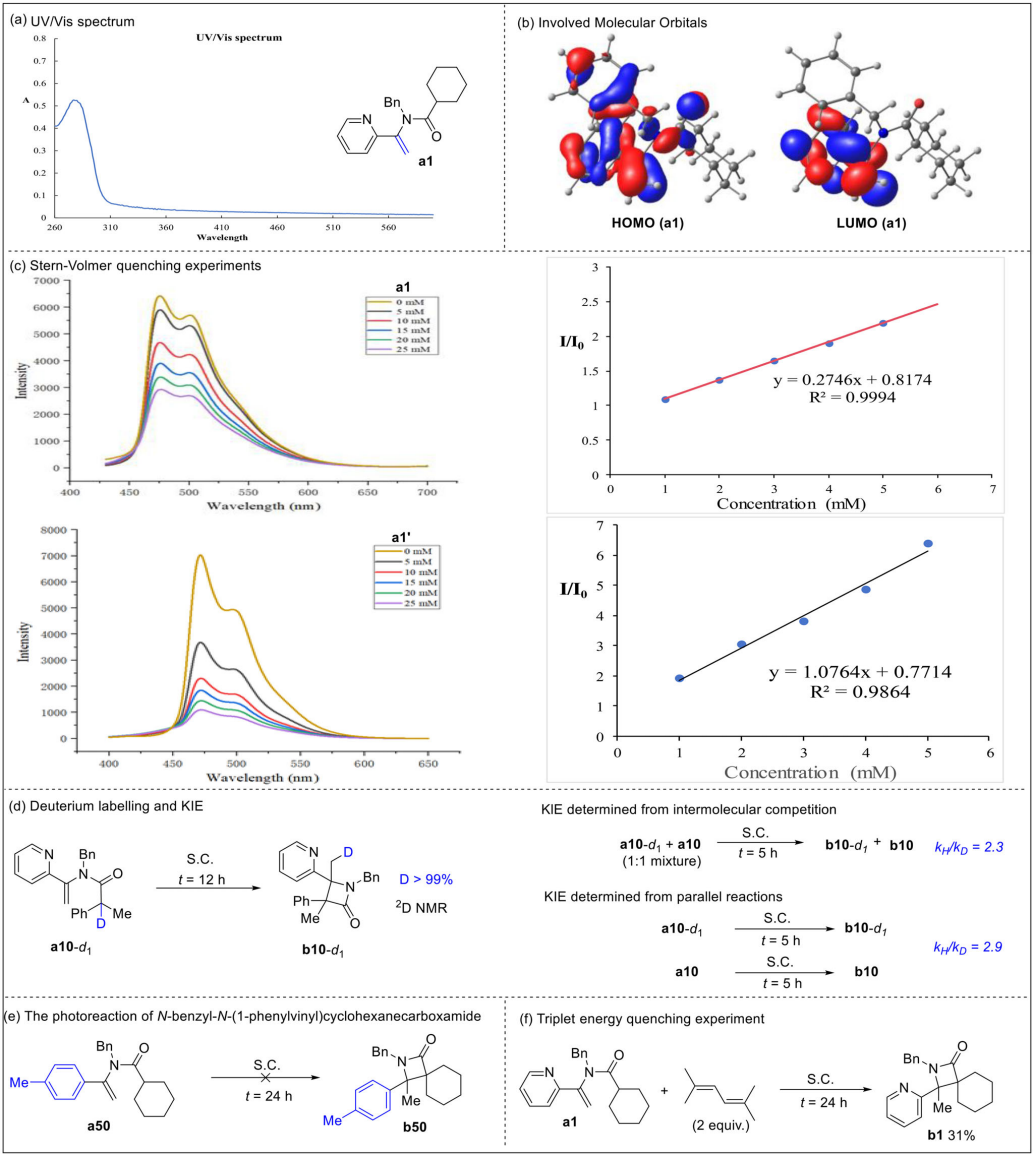
Mechanistic studies. Mechanistic experiments on the DHAT/cyclization cascade of enamides: S.C. is standard condition: **Ir‐1** (2 mol%), Zn(OTf)_2_ (20 mol%), DCM (c = 20 mm), λ_max_ = 427 nm. (a) UV/Vis spectrum for substrates **a1**. (b) Computed molecular orbitals (PCM(dichloromethane)‐TD‐B3LYP‐D3BJ/def2‐SVP//CPCM(dichloromethane)‐B3LYP‐D3BJ/def2‐SVP level) involved in the observed absorption (isosurface value of 0.04 a.u.). (c) Stern–Volmer quenching experiments. (d) The reaction of deuterated substrate **a10**‐*d*_1_ and KIE. (e) photochemical experiment of **a50**. (f) Triplet energy quenching experiments of **a1**.

To elucidate the reaction mechanism involved in this novel transformation, different control experiments were undertaken (Figure 3). First, the UV/Vis absorption properties of **a1** were studied (Figure 3a and Figure S3). The UV/Vis spectrum of **a1** revealed an absorption maximum at 277 nm, ε_277 nm_ = 2103.9 M^−1^cm^−1^, which can be tentatively assigned to a π‐π* transition [59]. Interestingly, upon adding different equiv. of Zn(OTf)_2_, the π‐π* transition did not lead to an obvious shift (see Figure S4 for details). Time‐dependent (TD) density functional theory (DFT) calculations were carried out to assign the nature of the absorption observed in the UV/Vis spectrum of **a1**. The calculations gave a vertical excitation energy of λ_calc_ = 298 nm, which is in reasonably good agreement with the experimental values of ca. 275 nm. This absorption mainly results from the HOMO→LUMO vertical transition, which, as depicted in Figure 3b, mainly involves the C═C double bond, therefore confirming the π−π^*^ nature of the absorption. Moreover, Stern–Volmer quenching experiments confirmed that the excited state of the photocatalyst was quenched by enamide **a1** or Lewis acid‐enamide **a1’** (Figure 3c). However, it was noted that the Lewis acid‐enamide **a1’** has higher quenching efficiencies than enamide **a1** (Figures S5 and S6), which might be a reason for the catalytic loading of Zn(OTf)_2_ in the reaction. Next, deuterium labeling and kinetic isotope effect (KIE) experiments were conducted to provide strong evidence about the DHAT process. It was found that product **b10**‐*d*_1_, which was obtained from deuterated substrate **a10**‐*d*_1_, exhibited one deuterium atom (D > 99%) specifically at the C1 position, a result consistent with the envisioned DHAT of the C1 position (Figure 3d). In addition, a primary KIE was supported by both intermolecular competition (k_H_/k_D_ = 2.3) and parallel reactions (k_H_/k_D_ = 2.9) experiments [60]. Moreover, under the standard reaction conditions, **a50** remained stable and was recovered essentially unchanged, with no detectable formation of the β‐lactam **b50** (Figure 3e), which highlights the crucial role of the coordinating pyridine nitrogen atom site in the photochemical transformation. Furthermore, a significant inhibition of the reaction was observed by adding 2,5‐dimethylhexa‐2,4‐diene, an efficient triplet quencher (Figure 3f), suggesting that the reaction proceeded through a triplet excited state [61].

**FIGURE 4:**
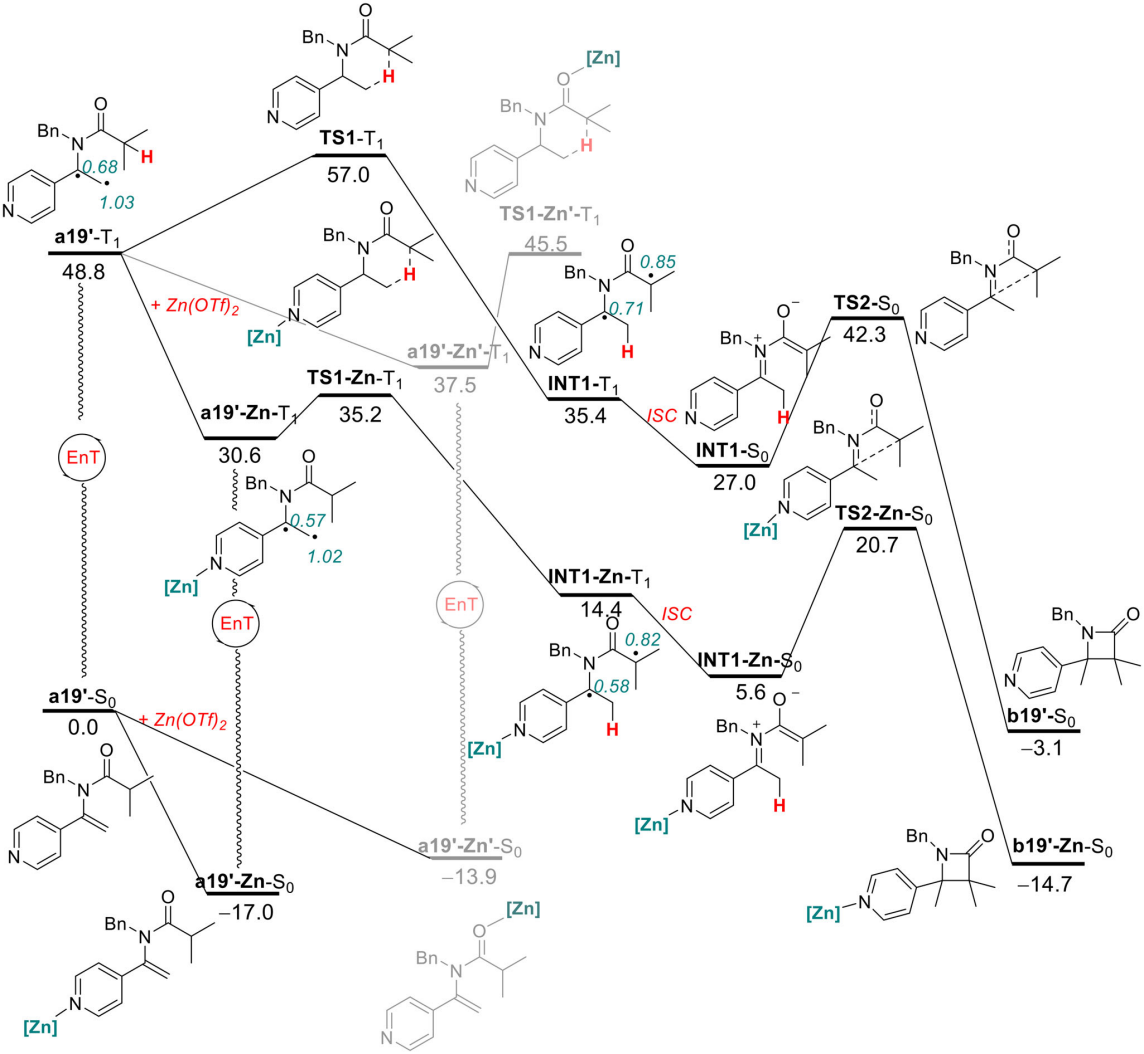
DFT studies. Computed reaction profiles for the transformation of **a19’** and its Zn(OTf)_2_ counterpart **a19’‐Zn**. Relative free energies (ΔG, at 298 K) are given in kcal/mol, and spin density values are shown in green. All data have been computed at the CPCM(dichloromethane)‐B3LYP‐D3BJ/def2‐TZVPP//CPCM(dichloromethane)‐B3LYP‐D3BJ/def2‐SVP level. [Zn] referes to Zn(OTf)_2_.

In addition, density functional theory (DFT) calculations at the dispersion‐corrected CPCM(dichloromethane)‐B3LYP‐D3BJ/def2‐TZVPP//CPCM(dichloromethane)‐B3LYP‐D3BJ/def2‐SVP level were carried out to gain more insight into the reaction mechanism and the influence of the Lewis acid catalyst on the process 2. To this end, we explored the transformation of substrate **a19’** into the β‐lactam **b19’** (where the cyclohexyl substituent was replaced by two methyl groups) both in the presence and absence of Zn(OTf)_2_ (Figure 4). Based on the above mechanistic studies and related transformations [51, 52], the uncatalyzed transformation begins with the energy transfer (EnT) from the excited photocatalyst to the C═C double bond of **a19’**, thus resulting in the formation of the triplet biradical **a19’‐T_1_** (ΔG_S‐T_ = 48.8 kcal/mol). Our calculations indicate that the spin density at the terminal carbon atom of the former alkene is higher than that at the adjacent carbon atom attached to the pyridyl group (1.03 e vs. 0.68 e). This facilitates the 1,5‐DHAT from the isopropyl group via transition state **TS1‐T_1_** (ΔG^≠^ = 8.2 kcal/mol), leading to the exergonic formation (ΔG = −13.4 kcal /mol) of triplet intermediate **INT1‐T_1_**. Subsequent intersystem crossing (ISC) produces the zwitterionic, closed‐shell singlet **INT1‐S_0_**, which readily undergoes cyclization via transition state **TS2‐S_0_** (ΔG^≠^ = 15.3 kcal/mol) to produce the observed β‐lactam **b19’** in a highly exergonic transformation (ΔG = −30.1 kcal/mol). Therefore, the computed reaction profile strongly resembles that proposed very recently by Smith and co‐workers on the photochemical formation of β‐lactams from *N*‐acyl enaminones [39]. The reaction profile computed in the presence of Zn(OTf)_2_ is rather similar in the sense that it also involves an initial 1,5‐DHAT in the triplet state, followed by cyclization in the singlet hypersurface. Two possible coordination modes were explored, namely that involving the pyridyl nitrogen atom and the alternative mode involving the amide carbonyl group. Not surprisingly, the former coordination mode is preferred in both the ground state (ΔΔG = 3.1 kcal/mol) and the excited triplet state (ΔΔG = 6.9 kcal/mol). Thus, from the more stable **a19’‐Zn‐T_1_** (ΔG_S‐T_ = 47.6 kcal/mol), the 1,5‐DHAT process takes place through **TS1‐Zn‐T_1_** (ΔG^≠^ = 4.6 kcal/mol) to produce intermediate **INT1‐Zn‐T_1_** in a highly exergonic step (ΔG = −16.2 kcal /mol). Therefore, the catalyzed 1,5‐DHAT is favored both kinetically (ΔΔG^≠^ = 3.6 kcal/mol) and thermodynamically (ΔΔG = 2.8 kcal/mol) over the analogous uncatalyzed reaction, which supports the beneficial role of the Lewis acid. Subsequent ISC leads to the analogous zwitterionic intermediate **INT1‐Zn‐S_0,_** which lies 8.8 kcal/mol below **INT1‐Zn‐T_1_**. From this species, the final cyclization step takes place via **TS2‐Zn‐S_0_** (ΔG^≠^ = 15.1 kcal/mol) to produce the observed β‐lactam again in a strongly exergonic reaction (ΔG = −20.3 kcal/mol). These mechanistic evidences indicate that the Lewis acid catalysis coordination clearly modulates the reactivity of the initial triplet diradical intermediates formed upon EnT from the excited photocatalyst to facilitate the cascade 1,5‐DHAT/cyclization reaction.

### 2.4 Synthetic Applications

**FIGURE 5:**
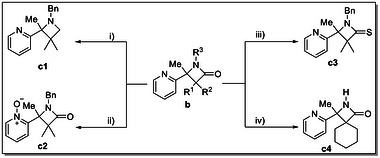
The transformations of β‐lactams. Reagents and conditions: (i) **b9**, LiAlH_4_, THF, 92%; (ii) **b9**, *m*‐CPBA, CH_2_Cl_2,_ 90%; (iii) **b9**, Lawesson reagent, THF, 94%; (iv) **b26**, CAN, MeCN, 40%.

Lastly, considering that *β*‐lactams are valuable intermediates in organic synthesis and biologically active molecules in medicinal chemistry, we have studied further derivatization reactions of the prepared lactams to demonstrate the usefulness of our methodology (Figure 5).

By using LiAlH_4_, *β*‐lactam **b9** can be easily reduced to access the azetidine **c1** for promising applications in modern medicinal chemistry and drug discovery [62]. Indeed, this approach provides a concise pathway for the chemical space exploration of azetidines. Meanwhile, the pyridine group in **b9** can be oxidized to afford pyridine oxide **c2** as a potential substrate for further valuable transformations [63]. Then, the carbonyl group of **b9** can be efficiently converted into the corresponding thiocarbonyl group product **c3** for convenient conversions upon treatment with the Lawesson reagent [64]. Furthermore, the benzyl protecting group on the nitrogen atom in **b26** can be effectively removed to get the product **c4**, which enables further incorporation into diverse bioactive molecules.

## 3 Conclusion

In summary, we have discovered a Lewis acid‐catalysed intramolecular DHAT and cyclization cascade of enamides, which allows access to various *β*‐lactams by energy transfer under visible light irradiation. This method enables modular and practical construction of an array of *β*‐lactams containing quaternary carbon centers and heteroaromatic rings, facilitating efficient modification of drug molecules with tolerance for a wide range of functional groups. Based on mechanistic evidences and DFT calculations, it is shown that the Lewis acid catalysis was crucial for the success of the processes by modulating the reactivity of the diradical intermediates derived from the excited enamides in intramolecular DHAT of a variety of C(sp^3^)−H bonds and subsequent cyclization. This distinctive activation mode of Lewis acid catalysis paves a new way of DHAT reactions for the transformation of C(sp^3^)−H bonds, which offers a number of opportunities for applications in organic synthesis and pharmaceuticals.

## Author Contributions

**Yao Huang**: methodology, software, data curation, investigation, formal analysis. **Kexue Xia**: conceptualization, software, formal analysis, Writing – review and editing, data curation. **Baoyu Yang **: methodology, software, data curation, investigation, formal analysis. **Yang Xiong**: conceptualization, methodology, software, data curation, supervision, formal analysis, investigation, funding acquisition, Writing – original draft, Writing – review and editing, project administration. **Xiang Zhou**: methodology, software, data curation, formal analysis, validation, investigation. **Israel Fernández**: software, data curation, formal analysis, Writing – review and editing, conceptualization.

## Funding

This study was supported by the National Natural Science Foundation of China (Grant No: 22301026), the Natural Science Foundation of Chongqing (Grant No. CSTB2024NSCQ‐MSX0206), the Venture & Innovation Support Program for Chongqing Overseas Returnees (Grant No. cx2024125), the Hongshen Young Scholars Program from Chongqing University (Grant No. 02470011080002), and the Science and Technology Research Program of Chongqing Municipal Education Commission (Grant No. KJQN202400483); PID2022‐139318NB‐I00 (funded by MICIU/AEI/10.13039/501100011033).

## Conflicts of Interest

The authors declare no conflicts of interest.

## Supporting information




**Supporting File**: advs76846‐sup‐0001‐SuppMat.docx.

## Data Availability

The data that supports the findings of this study are available in the supplementary material of this article.

## ENDNOTES

1 The assignment of the product **b29** was corroborated by single‐crystal X‐ray diffraction (CCDC 2554983).

2 See computational details in the supporting information.
