# Supplementary material for: Visible‐Light‐Mediated Lewis Acid‐Catalyzed Diradical Hydrogen Atom Transfer Reaction of Enamides
Source: Adv Sci (Weinh). 2026 Aug 6;13(59):e76846. doi: 10.1002/advs.76846 (PMC13445320; doi:10.1002/advs.76846)
Supplement: Supplementary file 1 [file ADVS-13-e76846-s001.docx]

**Supporting Information for**

Visible-Light-Mediated Lewis Acid-Catalyzed Diradical Hydrogen Atom Transfer Reaction of Enamides

Yao Huang,^1^ Baoyu Yang,^1^ Xiang Zhou,^1^ Israel Fernández,^2,*^ Kexue Xia,^3,*^ Yang Xiong^1,*^

^1^Chongqing Key Laboratory of Natural Product Synthesis and Drug Research, Innovative Drug Research Center, School of Pharmaceutical Sciences, Chongqing University, Chongqing 401331 (P. R. China). Email: yxiong@cqu.edu.cn.

^2^Departamento de Química Orgánica and Centro de Innovación en Química Avanzada (ORFEO-CINQA), Facultad de Ciencias Químicas, Universidad Complutense de Madrid, 28040-Madrid (Spain). Email: [israel@quim.ucm.es](mailto:israel@quim.ucm.es).

^3^College of Traditional Chinese Medicine, Chongqing Medical University, Chongqing 400016 (P. R. China). Email: x201057@163.com.

**Table of Contents**

[1. General Information	3](#_Toc234413611)

[2. Synthetic Procedures and Analytical Data	4](#_Toc234413612)

[2.1 The Synthesis of Enamides	4](#_Toc234413613)

[2.2 Visible-Light-Mediated Diradical Hydrogen Atom Transfer of Enamides	23](#_Toc234413614)

[3. UV/Vis Spectra	42](#_Toc234413615)

[4. Mechanistic Studies	43](#_Toc234413616)

[4.1 Stern-Volmer Quenching Experiments	43](#_Toc234413617)

[4.2 Deuteration Experiments and KIE	45](#_Toc234413618)

[4.3 Controlled Experiments	47](#_Toc234413619)

[4.4 Triplet Energy Quenching Experiment	48](#_Toc234413620)

[4.5 DFT Studies	49](#_Toc234413621)

[5. Transformations of the Products	73](#_Toc234413622)

[6. X-Ray Crystallographic Details	76](#_Toc234413623)

[6.1 X-Ray Crystallographic Details of **b10**	76](#_Toc234413624)

[6.2 X-Ray Crystallographic Details of **b24**	77](#_Toc234413625)

[6.3 X-Ray Crystallographic Details of **b29**	78](#_Toc234413626)

[6.4 X-Ray Crystallographic Details of **b43**	79](#_Toc234413627)

[7. References	80](#_Toc234413628)

[8. NMR-Spectra of Compounds	81](#_Toc234413629)

[8.1 NMR-Spectra of Enamides	81](#_Toc234413630)

[8.2 NMR-Spectra of Products of Photochemical Reactions	136](#_Toc234413631)

[8.3 NMR-Spectra of Transformations of the Products	197](#_Toc234413632)

[8.4 NMR-Spectra of the Deuterated Experiments	201](#_Toc234413633)

# **1. General Information**

All reactions, which are sensitive to air or moisture, were carried out in flame-dried glassware under argon pressure using standard Schlenk techniques. All glassware was dried with a heat gun under a stream of dry argon

Dry benzotrifluoride (PhCF_3_), dichloromethane (CH_2_Cl_2_), Acetonitrile (CH_3_CN) and other solvents were obtained from *Adamas* in the highest purity available and used without further purification, [water ≤50 ppm (by K.F.), 99.0%, safe dry, with molecular sieves, safe seal]. Technical solvents used for aqueous workup and for column chromatography [dichloromethane (CH_2_Cl_2_), ethyl acetate (EtOAc), *n*-hexane (Hex)] were distilled prior to use.

Photochemical experiments were performed in Kessil®PR160L lamps (PR160-427nm) and a fan was used as the photochemistry setup. 20 mL dried glass vials were used to run the photochemical reactions. The vials were placed at a distance of approximately 3-5 cm away from the lamp prior to irradiation at maximum intensity (100% power) of the lamp, (3 vials with one lamp). Prior to irradiation, the reaction mixture was evacuated/backfilled with Argon (3 times) or degassed by purging with argon in an ultrasonicating bath for 15 minutes.

Flash chromatography was performed on silica (Yantai, Jiangyou, 200-300 mesh) with the indicated eluent mixtures. Thin layer chromatography (TLC) was performed on silica coated glass plates (Yantai, Jiangyou, 2 mm) with detection by UV (λ = 254 and 366 nm).

UV/Vis spectra was measured on an Agilent Cary60 UV/Vis spectrometer. Fluorescence spectra was measured on a Hitachi F-7000 fluorescence spectrometer. Spectra were recorded using a precision cell made of quartz with a pathway of 0.1 and 1 cm. Solvents and concentrations are given for each spectrum.

^1^H, ^2^D, ^13^C, and ^19^F-NMR spectra were recorded at 303 K either on an Agilent 400MR DD2 (400 MHz) spectrometer and Agilent 600MR DD2 (600 MHz) spectrometer. NMR spectra were calibrated to the respective residual solvent signals of CDCl_3_ [δ [ppm] = (^1^H) = 7.26 ppm, δ [ppm] = (^13^C) = 77.16 ppm], CD_2_Cl_2_ [δ [ppm] = (^1^H) = 5.32 ppm, δ [ppm] = (^13^C) = 53.84], ^19^F NMR spectra were referenced to the ^19^F signal of CCl_3_F (δ = 0 ppm) as an internal standard. All ¹⁹F NMR spectra were recorded without proton decoupling. Apparent multiplets which occur as a result of coupling constant equality between magnetically non-equivalent protons are marked as virtual (*virt.*). The following abbreviations for single multiplicities were used: *br*-broad, m-multiplet, s-singlet, d-doublet, t-triplet, q-quartet, quint-quintet, sext-sextet.

High resolution mass spectroscopy (HR-MS) was performed on a *Thermo Scientific* QE Plus (ESI).

The mass analyzer type *quadrupole* was used for the HRMS measurements.

Unless otherwise stated, all reagents were purchased from commercial suppliers and used without further purification.

# 2. Synthetic Procedures and Analytical Data

## 2.1 The Synthesis of Enamides

General Procedure A

**Fig. S1.** General procedure for the synthesis of Enamides

**a1** as example

According to a literature procedure,^[1]^ replaced the nitrogen gas in the three-neck flask three times, then gradually added *N*-(1-(pyridin-2-yl)vinyl)cyclohexanecarboxamide^[2]^ (***s1*** *was used directly without purification after preparation*, 1.0 equiv.) in THF (0.1 M), while stirring in an ice bath for five minutes, slowly added NaH (1.5 equiv.) and stir the reaction mixture for 40 min at 0 °C, Then added a solution of Bromobenzene (1.5 equiv.) dropwise to this mixture at 0 °C and stirred the reaction mixture for 12 h at rt. Quenched with saturated ammonium chloride solution, extracted with EA and combined organic phase were washed with brine and dried over Na_2_SO_4_. After filtration and concentration in vacuo, the crude product was purified by column chromatography (PE/EA = 5/1) to **a1**.

*N*-benzyl-*N*-(1-(pyridin-2-yl)vinyl)cyclohexanecarboxamide (a1)

Following the general procedure A, **a1** was obtained as a white solid (531.5 mg, 1.66 mmol, 33% yield), after column chromatography (silica, PE/EtOAc 5:1).

**^1^H NMR** (400 MHz, CDCl_3_): δ [ppm] = 8.64 (d, *J* = 4.2 Hz, 1H), 7.71 – 7.60 (m, 1H), 7.35 – 7.17 (m, 7H), 6.31 (s, 1H), 5.04 (s, 1H), 4.70 (s, 2H), 2.43 – 2.33 (m, 1H), 1.72 – 1.44 (m, 7H), 1.29 – 1.16 (m, 1H), 1.10 – 0.95 (m, 2H).

**^13^C NMR** (101 MHz, CDCl_3_): δ [ppm] = 176.9, 153.6, 150.1, 145.3, 138.0, 137.2, 129.1, 128.4, 127.4, 123.5, 119.4, 117.7, 50.6, 42.2, 29.8, 25.7, 25.6.

**HRMS (ESI)** *m/z*: [M+Na] ^+^ calculated for C_21_H_24_N_2_ONa^+^: 343.1781, found: 343.1781.

N-**methyl-*N*-(1-(pyridin-2-yl)vinyl)cyclobutanecarboxamide (a2)**

Following the general procedure A, **a2** was obtained as a yellow liquid (466.8 mg, 2.16 mmol, 43.2% yield), after column chromatography (silica, PE/EtOAc 5:1).

**^1^H NMR** (400 MHz, CDCl_3_): δ [ppm] = 8.62 (d, *J* = 4.1 Hz, 1H), 7.72 – 7.64 (m, 1H), 7.28 – 7.18 (m, 2H), 6.35 (s, 1H), 5.32 (s, 1H), 3.21 – 3.14 (m, 1H), 3.11 (s, 3H), 2.40 – 2.21 (m, 2H), 1.87 – 1.69 (m, 4H).

**^13^C NMR** (101 MHz, CDCl_3_): δ [ppm] = 175.2, 153.2, 150.0, 147.4, 137.0, 123.4, 119.5, 115.9, 37.8, 35.9, 25.9, 18.0.

**HRMS (ESI)** *m/z*: [M+Na] ^+^ calculated for C_13_H_16_N_2_ONa^+^:239.1155, found: 239.1159.

***N*-benzyl-*N*-(1-(pyridin-2-yl)vinyl)cycloheptanecarboxamide (a3)**

Following the general procedure A, **a3** was obtained as a yellow solid (464.5 mg, 1.39 mmol, 27.8% yield), after column chromatography (silica, PE/EtOAc 10:1).

**^1^H NMR** (400 MHz, CDCl_3_): δ [ppm] = 8.64 (d, *J* = 4.2 Hz, 1H), 7.72 – 7.64 (m, 1H), 7.30 – 7.21 (m, 7H), 6.31 (s, 1H), 5.03 (s, 1H), 4.70 (s, 2H), 2.56 – 2.48 (m, 1H), 1.84 – 1.62 (m, 7H), 1.49 – 1.41 (m, 3H), 1.25 – 1.14 (m, 2H).

**^13^C NMR** (101 MHz, CDCl_3_): δ [ppm] = 178.2, 153.7, 150.1, 145.5, 138.1, 137.2, 129.1, 128.5, 127.4, 123.5, 119.4, 117.7, 50.5, 43.2, 31.8, 28.3, 26.7.

**HRMS (ESI)** *m/z*: [M+H] ^+^ calculated for C_22_H_27_N_2_O: 335.2118, found: 335.2114.

*N*-benzyl-4,4-difluoro-*N*-(1-(pyridin-2-yl)vinyl)cyclohexane-1-carboxamide **(a4)**

Following the general procedure A, **a4** was obtained as a yellow liquid (701.7 mg, 1.97 mmol, 39.3% yield), after column chromatography (silica, PE/EtOAc 5:1).

^1^**H NMR** (400 MHz, CDCl_3_): δ [ppm] = 8.64 (d, *J* = 4.1 Hz, 1H), 7.70 (t, *J* = 7.7 Hz, 1H), 7.35 – 7.20 (m, 7H), 6.29 (s, 1H), 5.06 (s, 1H), 4.70 (s, 2H), 2.53 – 2.41 (m, 1H), 2.16 – 2.01 (m, 2H), 2.01 – 1.89 (m, 2H), 1.80 – 1.70 (m, 2H), 1.62 – 1.42 (m, 2H).

**^19^F NMR** (564 MHz, CDCl_3_): δ [ppm] = -92.0 (d, *J* = 235.7 Hz, 1F), -101.4 (d, *J* = 236.5 Hz, 1F).

**^13^C NMR** (101 MHz, CDCl_3_): δ [ppm] = 175.1, 153.3, 150.2, 145.3, 137.7, 137.3, 129.1, 128.5, 127.6, 124.0 (d, *J* = 267.7 Hz), 123.8, 119.5, 117.9, 50.7, 39.8, 33.1, 32.8 (dd, *J* = 25.1, 23.6 Hz), 26.3 (d, *J* = 9.4 Hz).

**HRMS (ESI)** *m/z*: [M+Na] ^+^ calculated for C_21_H_22_F_2_N_2_ONa^+^: 379.1592, found: 379.1595.

*N*-benzyl-*N*-(1-(pyridin-2-yl)vinyl)tetrahydro-2H-pyran-4-carboxamide **(a5)**

Following the general procedure A, **a5** was obtained as a brown solid (715.3 mg, 2.22 mmol, 44.3% yield), after column chromatography (silica, PE/EtOAc 5:1).

**^1^H NMR** (400 MHz, CDCl_3_): δ [ppm] = 8.64 (d, *J* = 4.0 Hz, 1H), 7.69 (td, *J* = 7.8, 1.6 Hz, 1H), 7.29 – 7.19 (m, 7H), 6.30 (s, 1H), 5.05 (s, 1H), 4.70 (s, 2H), 3.96 – 3.85 (m, 2H), 3.27 – 3.14 (m, 2H), 2.69 – 2.59 (m, 1H), 2.03 – 1.91 (m, 2H), 1.54 – 1.41 (m, 2H).

**^13^C NMR** (101 MHz, CDCl_3_): δ [ppm] = 175.2, 153.5, 150.2, 145.4, 137.8, 137.2, 129.2, 128.5, 127.6, 123.7, 119.6, 117.9, 67.2, 50.9, 39.4, 29.6.

**HRMS (ESI)** *m/z*: [M+Na] ^+^ calculated for C_20_H_22_N_2_O_2_Na^+^: 345.1573, found: 345.1557.

**Benzyl 4-(benzyl(1-(pyridin-2-yl)vinyl)carbamoyl)piperidine-1-carboxylate (a6)**

Following the general procedure A, **a6** was obtained as a pale yellow solid (364.2 mg, 0.8 mmol, 16.0% yield), after column chromatography (silica, PE/EtOAc 5:1).

**^1^H NMR** (400 MHz, CDCl_3_): δ [ppm] = 8.64 (d, *J* = 4.2 Hz, 1H), 7.69 (t, *J* = 7.7 Hz, 1H), 7.35 – 7.23 (m, 12H), 6.28 (s, 1H), 5.10 (s, 2H), 5.06 (s, 1H), 4.70 (s, 2H), 4.14 (s, 2H), 2.63 – 2.47 (m, 3H), 1.90 – 1.78 (m, 2H), 1.60 (s, 2H).

**^13^C NMR** (101 MHz, CDCl_3_): δ [ppm] = 175.1, 155.2, 153.4, 150.2, 145.4, 137.7, 137.2, 136.9, 129.1, 128.5, 128.5, 128.0, 127.9, 127.6, 123.7, 119.6, 117.9, 67.1, 50.8, 43.3, 40.1, 28.8.

**HRMS (ESI)** *m/z*: [M+Na] ^+^ calculated for C_28_H_29_N_3_O_3_Na^+^: 478.2101, found: 478.2110.

***N*-benzyl-*N*-(1-(pyridin-2-yl)vinyl)cyclopent-3-ene-1-carboxamide (a7)**

Following the general procedure A, **a7** was obtained as a colorless crystal (924.8 mg, 3.04 mmol, 60.8% yield), after column chromatography (silica, PE/EtOAc 5:1).

**^1^H NMR** (400 MHz,CDCl_3_): δ [ppm] = δ 8.63 (d, *J* = 4.2 Hz, 1H), 7.68 (td, *J* = 7.9, 1.4 Hz, 1H), 7.30 – 7.21 (m, 7H), 6.34 (s, 1H), 5.57 (s, 2H), 5.03 (s, 1H), 4.73 (s, 2H), 3.21 – 3.12 (m, 1H), 2.70 (dd, *J* = 14.4, 6.9 Hz, 2H), 2.35 (dd, *J* = 14.1, 9.6 Hz, 2H).

**^13^C NMR** (101 MHz, CDCl_3_): δ [ppm] = 176.9, 153.5, 150.1, 145.4, 137.9, 137.1, 129.3, 129.0, 128.5, 127.5, 123.5, 119.5, 118.3, 50.9, 40.7, 38.2.

**HRMS (ESI)** *m/z*: [M+Na] ^+^ calculated for C_20_H_20_N_2_ONa^+^: 327.1468, found: 327.1475.

**1-Acetyl-N-benzyl-N-(1-(pyridin-2-yl)vinyl)pyrrolidine-2-carboxamide (a8)**

Following the general procedure A, **a8** was obtained as a brown solid (621.6 mg, 1.78 mmol, 35.6% yield), after column chromatography (silica, PE/EtOAc 5:1).

**^1^H NMR** (400 MHz, CDCl_3_): δ [ppm] = 8.61 (d, *J* = 4.5 Hz, 1H), 7.87 – 7.76 (m, 2H), 7.28 – 7.21 (m, 6H), 6.40 (s, 1H), 5.10 (s, 2H), 4.60 – 4.53 (m, 1H), 4.21 (s, 1H), 3.76 – 3.68 (m, 1H), 3.55 – 3.48 (m, 1H), 2.21 – 2.12 (m, 1H), 2.10 (s, 3H), 2.00 – 1.93 (m, 2H), 1.89 – 1.82 (m, 1H).

**^13^C NMR** (101 MHz, CDCl_3_): δ [ppm] = 172.5, 169.4, 149.8, 144.6, 137.4, 129.2, 128.5, 127.5, 123.9, 121.3, 119.3, 58.0, 50.5, 48.7, 31.0, 25.1, 22.6.

**HRMS (ESI)** *m/z*: [M+Na] ^+^ calculated for C_21_H_23_N_3_O_2_Na^+^: 372.1682, found: 372.1689.

*N*-benzyl-*N*-(1-(pyridin-2-yl)vinyl)isobutyramide **(a9)**

Following the general procedure A, **a9** was obtained as a yellow liquid (299.8 mg, 1.07 mmol, 21.4% yield), after column chromatography (silica, PE/EtOAc 5:1).

**^1^H NMR** (400 MHz, CDCl_3_): δ [ppm] = 8.63 (d, *J* = 3.7 Hz, 1H), 7.68 (t, *J* = 7.7 Hz, 1H), 7.31 – 7.18 (m, 7H), 6.33 (s, 1H), 5.06 (s, 1H), 4.72 (s, 2H), 2.75 – 2.61 (m, 1H), 1.07 (d, *J* = 6.6 Hz, 6H).

**^13^C NMR** (101 MHz, CDCl_3_): δ [ppm] = 178.0, 153.6, 150.1, 145.5, 138.0, 137.1, 129.2, 128.5, 127.5, 123.5, 119.5, 117.8, 53.6, 50.8, 32.1, 20.1.

**HRMS (ESI)** *m/z*: [M+Na] ^+^ calculated for C_18_H_20_N_2_ONa^+^: 303.1468, found: 303.1482.

***N*-benzyl-2-phenyl-*N*-(1-(pyridin-2-yl)vinyl)propanamide (a10)**

Following the general procedure A, **a10** was obtained as a white crystal (715.2 mg, 2.09 mmol, 41.8% yield), after column chromatography (silica, PE/EtOAc 5:1).

**^1^H NMR** (400 MHz, CDCl_3_): δ [ppm] = 8.63 (d, *J* = 4.0 Hz, 1H), 7.56 (s, 1H), 7.37 – 7.01 (m, 12H), 6.30 (s, 1H), 4.80 (s, 3H), 3.87 (d, *J* = 4.8 Hz, 1H), 1.44 (d, *J* = 6.9 Hz, 3H).

**^13^C NMR** (101 MHz, CDCl_3_): δ [ppm] = 174.4, 153.1, 149.9, 144.6, 142.0, 137.6, 137.0, 129.1, 128.4, 128.4, 127.7, 127.4, 126.7, 123.4, 119.6, 119.1, 50.8, 43.6, 20.7.

**HRMS (ESI)** *m/z*: [M+H] ^+^ calculated for C_23_H_23_N_2_O: 343.1805, found: 343.1803.

***N*-benzyl-2-phenyl-*N*-(1-(pyridin-2-yl)vinyl)butanamide (a11)**

Following the general procedure A, **a11** was obtained as a brown crystal (484.4 mg, 1.36 mmol, 27.1% yield), after column chromatography (silica, PE/EtOAc 3:1).

**^1^H NMR** (400 MHz, CDCl_3_): δ [ppm] = 8.62 (d, *J* = 4.1 Hz, 1H), 7.52 (s, 1H), 7.30 – 7.07 (m, 11H), 7.02 (s, 1H), 6.32 (s, 1H), 4.75 (s, 3H), 3.60 (s, 1H), 2.19 – 2.06 (m, 1H), 1.76 – 1.67 (m, 1H), 0.79 (t, *J* = 7.3 Hz, 3H).

**^13^C NMR** (101 MHz, CDCl_3_): δ [ppm] = 173.7,153.3, 150.0, 137.8, 136.9, 129.1, 128.4, 128.3, 128.1, 127.4, 126.7, 123.4, 119.8, 119.2, 51.5, 50.9, 28.8, 12.7.

**HRMS (ESI)** *m/z*: [M+Na] ^+^ calculated for C_24_H_24_N_2_ONa^+^: 379.1781, found: 379.1776.

***N*-benzyl-2-(4-chlorophenyl)-3-methyl-*N*-(1-(pyridin-2-yl)vinyl)butanamide (a12)**

Following the general procedure A, **a12** was obtained as a yellow liquid (1050.9 mg, 2.60 mmol, 51.9% yield), after column chromatography (silica, PE/EtOAc 10:1).

**^1^H NMR** (400 MHz, CDCl_3_): δ [ppm] = 8.64 (d, *J* = 3.8 Hz, 1H), 7.53 (s, 1H), 7.37 – 7.20 (m, 6H), 7.18 – 6.84 (m, 5H), 6.41 (s, 1H), 4.81 (s, 3H), 3.32 (s, 1H), 2.52 – 2.38 (m, 1H), 1.02 (d, *J* = 6.4 Hz, 3H), 0.57 (d, *J* = 6.7 Hz, 3H).

**^13^C NMR** (101 MHz, CDCl_3_): δ [ppm] = 173.3, 152.9, 149.9, 137.6, 136.9, 132.4, 129.9, 129.1, 128.4, 128.2, 127.5, 123.5, 119.9, 119.5, 50.9, 33.3, 22.1, 20.2.

**HRMS (ESI)** *m/z*: [M+H] ^+^ calculated for C_25_H_26_ClN_2_O: 405.1728, found: 405.1724

***N*-benzyl-*N*-(1-(4-methylpyridin-2-yl)vinyl)cyclohexanecarboxamide (a13)**

Following the general procedure A, **a13** was obtained as a white solid (137.0 mg, 0.41 mmol, 8.1% yield), after column chromatography (silica, PE/EtOAc 5:1).

**^1^H NMR** (400 MHz, CDCl_3_)): δ [ppm] = 8.48 (d, *J* = 4.9 Hz, 1H), 7.30 – 7.20 (m, 5H), 7.06 (d, *J* = 4.7 Hz, 1H), 7.00 (s, 1H), 6.30 (s, 1H), 5.03 (s, 1H), 4.70 (s, 2H), 2.42 – 2.35 (m, 1H), 2.31 (s, 3H), 1.71 – 1.53 (m, 7H), 1.25 – 1.15 (m, 1H), 1.09 – 0.97 (m, 2H).

**^13^C NMR** (101 MHz, CDCl_3_): δ [ppm] = 177.0, 153.5, 149.9, 148.4, 145.6, 138.1, 129.2, 128.4, 127.4, 124.4, 120.4, 117.4, 50.8, 42.3, 29.9, 25.8, 25.6, 21.3.

**HRMS (ESI)** *m/z*: [M+Na] ^+^ calculated for C_22_H_26_N_2_ONa^+^: 357.1937, found: 357.1951.

***N*-benzyl-*N*-(1-(5-fluoropyridin-2-yl)vinyl)cyclohexanecarboxamide (a14)**

Following the general procedure A, **a14** was obtained as a brown solid (618.9 mg, 1.83 mmol, 36.5% yield), after column chromatography (silica, PE/EtOAc 5:1).

**^1^H NMR** (400 MHz, CDCl_3_): δ [ppm] = 8.48 (d, *J* = 2.6 Hz, 1H), 7.41 – 7.32 (m, 1H), 7.28 – 7.19 (m, 6H), 6.21 (s, 1H), 5.03 (s, 1H), 4.68 (s, 2H), 2.40 – 2.30 (m, 1H), 1.69 – 1.53 (m, 7H), 1.23 – 1.14 (m, 1H), 1.11 – 0.97 (m, 2H).

**^19^F NMR** (564 MHz, CDCl_3_): δ [ppm] = -126.9 (s, 1F).

^1^**^3^C NMR** (101 MHz, CDCl_3_): δ [ppm] = 176.9, 159.3 (d, *J* = 264.3 Hz), 144.6, 138.7, 138.5, 137.9, 129.1, 128.5, 127.5, 123.6, 123.4, 120.6, 120.6, 117.5, 50.7, 42.3, 29.9, 25.7, 25.6.

**HRMS (ESI)** *m/z*: [M+Na] ^+^ calculated for C_21_H_23_FN_2_ONa^+^: 361.1687, found: 361.1688.

N-benzyl*-N*-(1-(4-chloropyridin-2-yl)vinyl)cyclohexanecarboxamide **(a15)**

Following the general procedure A, **a15** was obtained as a yellow solid (1041.1mg, 2.94 mmol, 58.7% yield), after column chromatography (silica, PE/EtOAc 4:1).

**^1^H NMR** (400 MHz, CDCl_3_): δ [ppm] = 8.52 (d, *J* = 5.2 Hz, 1H), 7.32 – 7.22 (m, 7H), 6.34 (s, 1H), 5.10 (s, 1H), 4.69 (s, 2H), 2.38 – 2.30 (m, 1H), 1.70 – 1.57 (m, 7H), 1.23 – 1.13 (m, 1H), 1.08 – 0.97 (m, 2H).

**^13^C NMR** (101 MHz, CDCl_3_): δ [ppm] = 176.8, 155.4, 150.9, 145.4, 144.6, 137.8, 129.2, 128.5, 127.6, 123.7, 120.0, 119.0, 50.7, 42.4, 29.9, 25.7, 25.6.

**HRMS (ESI)** *m/z*: [M+Na] ^+^ calculated for C_21_H_23_ClN_2_ONa^+^: 377.1391, found: 377.1419.

***N*-benzyl-*N*-(1-(5-bromopyridin-2-yl)vinyl)cyclohexanecarboxamide (a16)**

Following the general procedure A, **a16** was obtained as a white solid (525.5 mg, 1.32 mmol, 26.3% yield), after column chromatography (silica, PE/EtOAc 10:1).

**^1^H NMR** (400 MHz, CDCl_3_): δ [ppm] =8.67 (s, 1H), 7.78 (dd, *J* = 8.4, 2.1 Hz, 1H), 7.36 – 7.18 (m, 5H), 7.11 (d, *J* = 8.4 Hz, 1H), 6.31 (s, 1H), 5.08 (s, 1H), 4.68 (s, 2H), 2.39 – 2.29 (m, 1H), 1.89 – 1.79 (m, 1H), 1.69 – 1.58 (m, 5H), 1.36 – 1.18 (m, 2H), 1.14 – 0.96 (m, 2H).

**^13^C NMR** (101 MHz, CDCl_3_): δ [ppm] = 176.8, 152.3, 151.2, 144.7, 139.6, 137.8, 129.2, 128.5, 127.6, 120.7, 118.4, 50.7, 42.4, 29.9, 29.1, 25.9, 25.8, 25.7.

**HRMS (ESI)** *m/z*: [M+Na] ^+^ calculated for C_21_H_23_BrN_2_ONa^+^: 421.0886, found: 421.0879.

*N*-benzyl-*N*-(1-(5-(trifluoromethyl)pyridin-2-yl)vinyl)cyclohexanecarboxamide **(a17)**

Following the general procedure A, **a17** was obtained as a brown solid (283.4mg,0.73mmol, 14.6% yield), after column chromatography (silica, PE/EtOAc 4:1).

**^1^H NMR** (400 MHz, CDCl_3_): δ [ppm] = 8.88 (s, 1H), 7.90 (d, *J* = 7.3 Hz, 1H), 7.32 (d, *J* = 8.2 Hz, 1H), 7.27 – 7.15 (m, 5H), 6.44 (s, 1H), 5.18 (s, 1H), 4.69 (s, 2H), 2.37 – 2.25 (m, 1H), 1.70 – 1.56 (m, 7H), 1.24 – 1.16 (m, 1H), 1.10 – 0.95 (m, 2H).

**^19^F NMR** (564 MHz, CDCl_3_): δ [ppm] = -62.4 (s, 3F).

**^13^C NMR** (101 MHz, CDCl_3_): δ [ppm] = 176.8, 157.1, 147.0, 144.6, 137.7, 134.5, 129.2, 128.6, 127.7, 124.9 (d, *J* = 213.1 Hz), 120.3, 119.0, 50.7, 42.4, 29.9, 25.7, 25.6.

**HRMS (ESI)** *m/z*: [M+Na] ^+^ calculated for C_22_H_23_F_3_N_2_ONa^+^: 411.1655, found: 411.1660.

***N*-benzyl-*N*-(1-(pyridin-3-yl)vinyl)cyclohexanecarboxamide (a18)**

Following the general procedure A, **a18** was obtained as a brown crystal (630.8 mg, 1.97 mmol, 39.4% yield), after column chromatography (silica, PE/EtOAc 5:1).

**^1^H NMR** (400 MHz, CDCl_3_): δ [ppm] = 8.68 (s, 1H), 8.59 (d, *J* = 4.2 Hz, 1H), 7.59 (d, *J* = 8.0 Hz, 1H), 7.34 – 7.18 (m, 6H), 5.67 (s, 1H), 4.97 (s, 1H), 4.64 (s, 2H), 2.53 – 2.43 (m, 1H), 1.75 – 1.55 (m, 7H), 1.25 – 1.15 (m, 1H), 1.14 – 0.96 (m, 2H).

**^13^C NMR** (101 MHz, CDCl_3_): δ [ppm] = 176.7, 150.2, 147.6, 143.8, 137.6, 133.1, 132.0, 129.1, 128.6, 127.6, 123.7, 115.7, 50.2, 42.3, 30.2, 25.7, 25.7.

**HRMS (ESI)** *m/z*: [M+Na] ^+^ calculated for C_21_H_24_N_2_ONa^+^: 343.1781, found: 343.1780.

***N*-benzyl-*N*-(1-(pyridin-4-yl)vinyl)cyclohexanecarboxamide (a19)**

Following the general procedure A, **a19** was obtained as a brown crystal (285.0 mg, 0.89 mmol, 17.8% yield), after column chromatography (silica, PE/EtOAc 1:1).

**^1^H NMR** (400 MHz, CDCl_3_): δ [ppm] = 8.62 (d, *J* = 5.6 Hz, 2H), 7.23 (dt, *J* = 12.4, 7.0 Hz, 7H), 5.83 (s, 1H), 5.06 (s, 1H), 4.65 (s, 2H), 2.43 – 2.33 (m, 1H), 1.71 – 1.56 (m, 7H), 1.24 – 1.16 (m, 1H), 1.08 – 0.98 (m, 2H).

**^13^C NMR** (101 MHz, CDCl_3_): δ [ppm] = 176.6, 150.7, 137.6, 129.0, 128.6, 127.6, 120.0, 117.6, 50.3, 42.3, 30.0, 25.7, 25.6.

**HRMS (ESI)** *m/z*: [M+H] ^+^ calculated for C_21_H_25_N_2_O: 321.1961, found: 321.1957.

***N*-benzyl-*N*-(1-(isoquinolin-1-yl)vinyl)cyclohexanecarboxamide (a20)**

Following the general procedure A, **a20** was obtained as a yellow solid (547.9 mg, 1.48 mmol, 29.5 % yield), after column chromatography (silica, PE/EtOAc 6:1).

**^1^H NMR** (400 MHz, CDCl_3_): δ [ppm] = 8.54 (d, *J* = 4.1 Hz, 1H), 8.19 (d, *J* = 5.2 Hz, 1H), 7.86 (d, *J* = 8.1 Hz, 1H), 7.69 (t, *J* = 7.4 Hz, 1H), 7.62 (d, *J* = 5.2 Hz, 1H), 7.56 (t, *J* = 7.3 Hz, 1H), 7.33 – 7.16 (m, 5H), 5.51 (s, 2H), 4.76 (s, 2H), 2.79 (s, 1H), 1.74 – 1.55 (m, 7H), 1.24 – 1.15 (m, 1H), 1.08 – 0.93 (m, 2H).

**^13^C NMR** (101 MHz, CDCl_3_): δ [ppm] = 177.5, 156.1, 144.3, 142.2, 138.6, 137.0, 130.2, 128.6, 128.4, 127.7, 127.5, 127.1, 126.8, 125.9, 121.0, 119.8, 50.8, 42.4, 30.1, 25.9, 25.8.

**HRMS (ESI)** *m/z*: [M+H] ^+^ calculated for C_25_H_27_N_2_O: 371.2094, found: 371.2092.

***N*-methyl-*N*-(1-(pyridin-2-yl)vinyl)cyclohexanecarboxamide (a21)**

Following the general procedure A, **a21** was obtained as a yellow liquid (693.5 mg, 2.84 mmol, 56.7% yield), after column chromatography (silica, PE/EtOAc 5:1).

**^1^H NMR** (400 MHz, CDCl_3_): δ [ppm] = 8.66 (d, *J* = 3.9 Hz, 1H), 7.72 (t, *J* = 7.7 Hz, 1H), 7.30 – 7.22 (m, 2H), 6.41 (s, 1H), 5.43 (s, 1H), 3.13 (s, 3H), 2.49 – 2.34 (m, 1H), 1.70 – 1.44 (m, 7H), 1.24 – 1.14 (m, 1H), 1.12 – 0.95 (m, 2H).

**^13^C NMR** (101 MHz, CDCl_3_): δ [ppm] = 176.1, 152.6, 149.2, 147.0, 136.2, 122.6, 118.5, 114.7, 41.0, 35.0, 28.9, 24.8, 24.7.

**HRMS (ESI)** *m/z*: [M+Na] ^+^ calculated for C_15_H_20_N_2_ONa^+^: 267.1468, found: 267.1468.

***N*-(4-fluorobenzyl)-*N*-(1-(pyridin-2-yl)vinyl)cyclohexanecarboxamide (a22)**

Following the general procedure A, **a22** as obtained as a brown crystal (517.4 mg, 1.53 mmol, 30.6% yield), after column chromatography (silica, PE/EtOAc 3:1).

**^1^H NMR** (400 MHz, CDCl_3_): δ [ppm] = 8.64 (d, *J* = 4.3 Hz, 1H), 7.68 (td, *J* = 7.8, 1.3 Hz, 1H), 7.29 – 7.18 (m, 4H), 6.94 (t, *J* = 8.6 Hz, 2H), 6.32 (s, 1H), 5.02 (s, 1H), 4.64 (s, 2H), 2.45 – 2.33 (m, 1H), 1.70 – 1.52 (m, 7H), 1.23 – 1.12 (m, 1H), 1.11 – 0.95 (m, 2H).

**^19^F NMR** (564 MHz, CDCl_3_): δ [ppm] = -115.3 (s, 1F).

**^13^C NMR** (101 MHz, CDCl_3_): δ [ppm] = 176.0, 161.3 (d, *J* = 245.6 Hz), 152.6, 149.2, 144.4, 136.2, 132.9, 129. 9, 122.6, 118.5, 116.7, 114.4, 114.1, 48.9, 41.3, 28.9, 24.8, 24.6.

**HRMS (ESI)** *m/z*: [M+Na] ^+^ calculated for C_21_H_23_FN_2_ONa^+^: 361.1687, found: 361.1694.

***N*-(4-chlorobenzyl)-*N-*(1-(pyridin-2-yl)vinyl)cyclohexanecarboxamide (a23)**

Following the general procedure A, **a23** was obtained as a white crystal (301.0 mg, 0.85 mmol, 16.9 % yield), after column chromatography (silica, PE/EtOAc 5:1).

**^1^H NMR** (400 MHz, CDCl_3_): δ [ppm] = 8.64 (d, *J* = 4.2 Hz, 1H), 7.68 (td, *J* = 7.8, 1.3 Hz, 1H), 7.27 – 7.19 (m, 6H), 6.30 (s, 1H), 5.03 (s, 1H), 4.63 (s, 2H), 2.42 – 2.34 (m, 1H), 1.68 – 1.52 (m, 7H), 1.23 – 1.14 (m, 1H), 1.11 – 0.97 (m, 2H).

**^13^C NMR** (101 MHz, CDCl_3_): δ [ppm] = 177.0, 153.5, 150.2, 145.4, 137.2, 136.6, 133.3, 130.6, 128.6, 123.6, 119.5, 117.7, 50.0, 42.2, 29.9, 25.7, 25.6.

**HRMS (ESI)** *m/z*: [M+H] ^+^ calculated for C_21_H_24_ClN_2_O: 355.1572, found: 355.1569.

***N*-(4-bromobenzyl)-*N-*(1-(pyridin-2-yl)vinyl)cyclohexanecarboxamide (a24)**

Following the general procedure A, **a24** was obtained as a white solid (760.6 mg, 1.91 mmol, 38.2% yield), after column chromatography (silica, PE/EtOAc 5:1).

**^1^H NMR** (400 MHz, CDCl_3_): δ [ppm] = 8.67 – 8.62 (m, 1H), 7.72 – 7.66 (m, 1H), 7.38 (d, *J* = 8.2 Hz, 2H), 7.28 – 7.22 (m, 2H), 7.15 (d, *J* = 8.2 Hz, 2H), 6.31 (s, 1H), 5.03 (s, 1H), 4.62 (s, 2H), 2.44 – 2.34 (m, 1H), 1.69 – 1.51 (m, 7H), 1.22 – 1.12 (m, 1H), 1.10 – 0.96 (m, 2H).

**^13^C NMR** (101 MHz, CDCl_3_): δ [ppm] = 177.0, 153.4, 150.1, 145.4, 137.2, 131.5, 130.9, 123.6, 121.5, 119.5, 117.7, 50.0, 42.2, 29.9, 25.7, 25.6.

**HRMS (ESI)** *m/z*: [M+H] ^+^ calculated for C_21_H_24_BrN_2_O: 399.1067, found: 399.1065.

***N*-(3,4-difluorobenzyl)-*N*-(1-(pyridin-2-yl)vinyl)cyclohexanecarboxamide (a25)**

Following the general procedure A, **a25** was obtained as a yellow solid (573.5 mg, 1.61 mmol, 32.2% yield), after column chromatography (silica, PE/EtOAc 5:1).

**^1^H NMR** (400 MHz, CDCl_3_): δ [ppm] = 8.64 (d, *J* = 4.1 Hz, 1H), 7.70 (td, *J* = 7.8, 1.7 Hz, 1H), 7.28 – 7.22 (m, 2H), 7.16 – 7.10 (m, 1H), 7.07 – 6.94 (m, 2H), 6.31 (s, 1H), 5.06 (s, 1H), 4.61 (s, 2H), 2.44 – 2.35 (m, 1H), 1.69 – 1.52 (m, 7H), 1.23 – 1.14 (m, 1H), 1.08 – 0.96 (m, 2H).

**^19^F NMR** (564 MHz, CDCl_3_): δ [ppm] = -137.7 – -137.8 (m, 1F), -139.7 – -139.9 (m, 1F).

**^13^C NMR** (101 MHz, CDCl_3_): δ [ppm] = 177.1, 166.2 (d, *J* = 220.7 Hz), 162.2 (d, *J* = 213.3 Hz), 153.4, 150.2, 145.5, 137.2, 135.1, 125.2, 123.7, 119.5, 118.1 (d, *J* = 17.2 Hz), 117.7, 117.1 (d, *J* = 17.1 Hz), 49.8, 42.2, 29.9, 25.7, 25.6.

**HRMS (ESI)** *m/z*: [M+H] ^+^ calculated for C_21_H_23_F_2_N_2_O: 379.1592, found: 379.1598.

***N*-(3-fluoro-4-methoxybenzyl)-*N*-(1-(pyridin-2-yl)vinyl)cyclohexanecarboxamide (a26)**

Following the general procedure A, **a26** was obtained as a brown crystal (603.8 mg, 1.64 mmol, 32.8% yield), after column chromatography (silica, PE/EtOAc 5:1).

**^1^H NMR** (400 MHz, CDCl_3_): δ [ppm] = 8.64 (d, *J* = 4.1 Hz, 1H), 7.68 (t, *J* = 7.7 Hz, 1H), 7.30 – 7.21 (m, 2H), 7.06 – 6.94 (m, 2H), 6.83 (t, *J* = 8.4 Hz, 1H), 6.32 (s, 1H), 5.04 (s, 1H), 4.59 (s, 2H), 3.84 (s, 3H), 2.42 – 2.32 (m, 1H), 1.69 – 1.49 (m, 7H), 1.23 – 1.11 (m, 1H), 1.10 – 0.96 (m, 2H).

**^13^C NMR** (101 MHz, CDCl_3_): δ [ppm] = 177.0, 153.5, 152.2 (d, *J* = 245.5 Hz), 150.1, 145.3, 137.2, 131.1, 125.0, 119.5, 117.7, 117.0, 116.8, 113.1, 56.3, 49.8, 42.2, 29.9, 25.7, 25.6.

**^19^F NMR** (564 MHz, CDCl_3_): δ [ppm] = -135.26 (s, 1F).

**HRMS (ESI)** *m/z*: [M+Na] ^+^ calculated for C_22_H_25_FN_2_O_2_Na^+^: 391.1792, found: 391.1787.

***N*-(cyclohexanecarbonyl)-*N*-(1-(pyridin-2-yl)vinyl)benzamide (a27)**

Following the general procedure A, **a27** was obtained as a brown solid (137.0 mg, 0.41 mmol, 8.1% yield), after column chromatography (silica, PE/EtOAc 5:1).

**^1^H NMR** (400 MHz, CDCl_3_): δ [ppm] = 8.59 (d, *J* = 4.7 Hz, 1H), 7.89 – 7.83 (m, 2H), 7.71 – 7.65 (m, 1H), 7.55 (d, *J* = 8.0 Hz, 1H), 7.46 (t, *J* = 7.4 Hz, 1H), 7.35 (t, *J* = 7.6 Hz, 2H), 7.22 (dd, *J* = 6.7, 4.9 Hz, 1H), 5.98 (s, 1H), 5.27 (s, 1H), 2.98 – 2.84 (m, 1H), 2.10 – 2.00 (m, 2H), 1.75 (d, *J* = 6.4 Hz, 2H), 1.64 (d, *J* = 4.1 Hz, 1H), 1.53 – 1.41 (m, 2H), 1.29 – 1.19 (m, 3H).

**^13^C NMR** (101 MHz, CDCl_3_): δ [ppm] = 181.5, 171.1, 137.0, 133.4, 132.3, 130.2, 129.1, 128.8, 128.5, 128.1, 127.5, 43.1, 29.7, 29.0, 25.9, 25.7, 25.5.

**HRMS (ESI)** *m/z*: [M+H] ^+^ calculated for C_21_H_23_N_2_O_2_: 335.1754, found: 335.1746.

***N*-(hex-5-en-1-yl)-*N*-(1-(pyridin-2-yl)vinyl)isobutyramide (a28)**

Following the general procedure A, **a28** was obtained as a yellow liquid (144.3 mg, 0.53 mmol, 10.5% yield), after column chromatography (silica, PE/EtOAc 8:1).

**^1^H NMR** (400 MHz, CDCl_3_): δ [ppm] = 8.65 (d, *J* = 3.7 Hz, 1H), 7.71 (td, *J* = 7.8, 1.5 Hz, 1H), 7.26 (dd, *J* = 7.5, 3.8 Hz, 2H), 6.50 (s, 1H), 5.83 – 5.65 (m, 1H), 5.40 (s, 1H), 5.01 – 4.85 (m, 2H), 3.49 (s, 2H), 2.77 – 2.63 (m, 1H), 2.11 – 2.01 (m, 2H), 1.60 (dt, *J* = 15.5, 7.6 Hz, 2H), 1.39 (dt, *J* = 15.0, 7.5 Hz, 2H), 1.05 (d, *J* = 6.7 Hz, 6H).

**^13^C NMR** (101 MHz, CDCl_3_): δ [ppm] = 177.8, 153.6, 150.1, 146.0, 138.7, 137.2, 123.6, 119.5, 117.0, 114.7, 47.0, 33.5, 31.9, 27.6, 26.2, 20.2.

**HRMS (ESI)** *m/z*: [M+H] ^+^ calculated for C_17_H_25_N_2_O: 273.1961, found: 273.1960.

***N*-benzyl-2-phenyl-*N*-(1-(pyridin-2-yl)vinyl)acetamide(a29)**

Following the general procedure A, **a29** was obtained as a red liquid (869.7 mg, 2.65 mmol, 52.9% yield), after column chromatography (silica, PE/EtOAc 5:1).

**^1^H NMR** (400 MHz, CDCl_3_): δ [ppm] = 8.64 (d, *J* = 4.2 Hz, 1H), 7.61 (td, *J* = 7.8, 1.5 Hz, 1H), 7.27 – 7.11 (m, 12H), 6.33 (s, 1H), 5.00 (s, 1H), 4.71 (s, 2H), 3.64 (s, 2H).

**^13^C NMR** (101 MHz, CDCl_3_): δ [ppm] = 171.4, 153.2, 150.1, 145.3, 137.5, 137.0, 135.6, 129.3, 128.4, 127.5, 126.7, 123.5, 119.8, 119.0, 50.9, 40.9.

**HRMS (ESI)** *m/z*: [M+Na] ^+^ calculated for C_22_H_20_N_2_ONa^+^: 351.1468, found: 351.1471.

***N*-benzyl-*N*-(1-(pyridin-2-yl)vinyl)-2-(p-tolyl)acetamide (a30)**

Following the general procedure A, **a30** was obtained as a red liquid (677.6 mg, 1.98 mmol, 39.6% yield), after column chromatography (silica, PE/EtOAc 5:1).

**^1^H NMR** (400 MHz, CDCl_3_): δ [ppm] = 8.64 (d, *J* = 4.2 Hz, 1H), 7.63 (t, *J* = 7.1 Hz, 1H), 7.26 – 7.12 (m, 7H), 7.10 – 6.99 (m, 4H), 6.35 (s, 1H), 5.01 (s, 1H), 4.71 (s, 2H), 3.59 (s, 2H), 2.28 (s, 3H).

**^13^C NMR** (101 MHz, CDCl_3_): δ [ppm] = 171.7, 153.1, 150.1, 145.1, 137.5, 137.1, 136.2, 132.4, 129.3, 129.2, 129.1, 128.4, 127.5, 123.5, 119.8, 119.1, 50.9, 40.4, 21.2.

**HRMS (ESI)** *m/z*: [M+Na] ^+^ calculated for C_23_H_22_N_2_ONa^+^: 365.1624, found: 365.1624.

*N*-benzyl-2-(4-fluorophenyl)-*N-*(1-(pyridin-2-yl)vinyl)acetamide **(a31)**

Following the general procedure A, **a31** was obtained as a red liquid (931.0 mg,2.69 mmol, 53.9% yield), after column chromatography (silica, PE/EtOAc 5:1).

**^1^H NMR** (400 MHz, CDCl_3_): δ [ppm] = 8.64 (d, *J* = 4.3 Hz, 1H), 7.63 (t, *J* = 7.4 Hz, 1H), 7.30 – 7.10 (m, 9H), 6.90 (t, *J* = 8.6 Hz, 2H), 6.34 (s, 1H), 5.02 (s, 1H), 4.71 (s, 2H), 3.60 (s, 2H).

**^19^F NMR** (564 MHz, CDCl_3_): δ [ppm] = -115.3 (s, 1F).

**^13^C NMR** (101 MHz, CDCl_3_): δ [ppm] = 171.2, 161.8 (d, *J* = 244.4 Hz), 163.0, 160.6, 153.1, 150.1, 145.3, 137.4, 137.0, 130.3 (d, *J* = 7.9 Hz), 129.3, 128.4, 127.6, 123.6, 119.7, 118.9, 115.1 (d, *J* = 21.3 Hz), 50.9, 39.9.

**HRMS (ESI)** m/z: [M+Na] ^+^ calculated for C_22_H_19_FN_2_ONa^+^: 369.1374, found: 369.1383.

***N*-benzyl-2-(4-chlorophenyl)-*N*-(1-(pyridin-2-yl)vinyl)acetamide (a32)**

Following the general procedure A, **a32** was obtained as a yellow liquid (680.7 mg, 1.88 mmol, 37.6 % yield), after column chromatography (silica, PE/EtOAc 5:1).

**1H NMR** (400 MHz, CDCl3): δ [ppm] = 8.64 (d, J = 4.3 Hz, 1H), 7.64 (t, J = 7.6 Hz, 1H), 7.37 – 7.14 (m, 9H), 7.10 (d, J = 8.2 Hz, 2H), 6.33 (s, 1H), 5.02 (s, 1H), 4.71 (s, 2H), 3.60 (s, 2H).

**^13^C NMR** (101 MHz, CDCl_3_): δ [ppm] = 170.9, 153.0, 150.1, 145.3, 137.4, 137.1, 134.0, 132.6, 130.7, 129.3, 128.5, 127.6, 123.6, 119.8, 119.0, 51.0, 40.2.

**HRMS (ESI)** *m/z*: [M+H] ^+^ calculated for C_22_H_20_ClN_2_O: 363.1259, found: 363.1262.

***N*-benzyl-2-(4-methoxyphenyl)-*N*-(1-(pyridin-2-yl)vinyl)acetamide (a33)**

Following the general procedure A, **a33** was obtained as a yellow liquid (1414.9 mg, 3.95 mmol, 77.9% yield), after column chromatography (silica, PE/EtOAc 5:1).

**^1^H NMR** (400 MHz, CDCl_3_): δ [ppm] = 8.65 (d, *J* = 4.3 Hz, 1H), 7.63 (t, *J* = 7.6 Hz, 1H), 7.31 – 7.20 (m, 6H), 7.17 (d, *J* = 7.9 Hz, 1H), 7.08 (d, *J* = 8.3 Hz, 2H), 6.76 (d, *J* = 8.4 Hz, 2H), 6.36 (s, 1H), 5.02 (s, 1H), 4.71 (s, 2H), 3.76 (s, 3H), 3.57 (s, 2H).

**^13^C NMR** (101 MHz, CDCl_3_): δ [ppm] = 171.8, 158.4, 153.2, 150.1, 145.2, 137.6, 137.1, 130.4, 129.3, 128.44, 127.6, 123.5, 119.8, 119.1, 113.9, 55.4, 50.9, 40.0.

**HRMS (ESI)** *m/z*: [M+Na] ^+^ calculated for C_23_H_22_N_2_O_2_Na^+^: 381.1573, found: 381.1575.

***N*-benzyl-*N*-(1-(pyridin-2-yl)vinyl)-2-(thiophen-2-yl)acetamide (a34)**

Following the general procedure A, **a34** was obtained as a yellow liquid (604.7 mg, 1.81 mmol, 36.1 % yield), after column chromatography (silica, PE/EtOAc 5:1).

**^1^H NMR** (400 MHz, CDCl_3_): δ [ppm] = 8.65 (d, *J* = 4.2 Hz, 1H), 7.65 (td, *J* = 7.8, 1.7 Hz, 1H), 7.32 – 7.19 (m, 7H), 7.17 – 7.12 (m, 1H), 6.88 – 6.84 (m, 1H), 6.75 (d, *J* = 2.5 Hz, 1H), 6.34 (s, 1H), 5.08 (s, 1H), 4.73 (s, 2H), 3.85 (s, 2H).

**^13^C NMR** (101 MHz, CDCl_3_): δ [ppm] = 170.4, 150.2, 145.2, 137.3, 137.2, 136.9, 129.4, 128.5, 127.6, 126.5, 126.4, 124.8, 123.6, 119.8, 119.1, 51.0, 35.2.

**HRMS (ESI)** *m/z*: [M+Na] ^+^ calculated for C_20_H_18_N_2_OSNa^+^: 357.1032, found: 357.1031.

N-**benzyl-2-methoxy-*N*-(1-(pyridin-2-yl)vinyl)acetamide (a35)**

Following the general procedure A, **a35** was obtained as a red liquid (719.4 mg, 2.55 mmol, 50.9% yield), after column chromatography (silica, PE/EtOAc 5:1).

**^1^H NMR** (400 MHz, CDCl_3_): δ [ppm] = 8.62 (d, *J* = 4.2 Hz, 1H), 7.67 (td, *J* = 7.8, 1.5 Hz, 1H), 7.31 – 7.19 (m, 7H), 6.25 (s, 1H), 5.06 (s, 1H), 4.71 (s, 2H), 4.01 (s, 2H), 3.35 (s, 3H).

**^13^C NMR** (101 MHz, CDCl_3_): δ [ppm] = 169.5, 152.7, 150.1, 144.0, 137.1, 129.3, 128.4, 127.6, 123.6, 119.7, 118.8, 70.3, 59.2, 50.6.

**HRMS (ESI)** *m/z*: [M+Na] ^+^ calculated for C_17_H_18_N_2_O_2_Na^+^: 305.1260, found: 305.1273.

***N*-benzyl-2-(benzyloxy)-*N*-(1-(pyridin-2-yl)vinyl)acetamide (a36)**

Following the general procedure A, **a36** was obtained as a yellow liquid (1490.1 mg, 4.16 mmol, 83.2 % yield), after column chromatography (silica, PE/EtOAc 5:1).

**^1^H NMR** (400 MHz, CDCl_3_): δ [ppm] = 8.61 (d, *J* = 4.3 Hz, 1H), 7.64 (td, *J* = 7.8, 1.7 Hz, 1H), 7.38 – 7.17 (m, 12H), 6.21 (s, 1H), 5.04 (s, 1H), 4.72 (s, 2H), 4.58 (s, 2H), 4.12 (s, 2H).

**^13^C NMR** (101 MHz, CDCl_3_): δ [ppm] = 169.7, 152.7, 150.0, 144.0, 137.6, 137.2, 129.4, 128.5, 128.4, 128.1, 127.8, 127.7, 123.6, 119.8, 118.8, 73.3, 67.9, 50.7.

**HRMS (ESI)** *m/z*: [M+Na] ^+^ calculated for C_23_H_22_N_2_O_2_Na^+^: 381.1573, found: 381.1584.

***N*-methyl-*N*-(1-(pyridin-2-yl)vinyl)propionamide (a37)**

Following the general procedure A, **a37** was obtained as a yellow liquid (344.1 mg, 1.81 mmol, 36.2 % yield), after column chromatography (silica, PE/EtOAc 5:1).

**^1^H NMR** (400 MHz, CDCl_3_): δ [ppm] = 8.65 (d, *J* = 3.6 Hz, 1H), 7.73 (t, *J* = 7.6 Hz, 1H), 7.33 – 7.24 (m, 2H), 6.42 (s, 1H), 5.45 (s, 1H), 3.17 (s, 3H), 2.26 (dd, *J* = 14.6, 7.2 Hz, 2H), 1.07 (t, *J* = 7.4 Hz, 3H).

**^13^C NMR** (101 MHz, CDCl_3_): δ [ppm] = 174.4, 153.2, 150.1, 147.9, 137.2, 123.5, 119.5, 116.3, 35.9, 27.0, 9.9.

**HRMS (ESI)** *m/z*: [M+Na]^+^ calculated for C_11_H_14_N_2_ONa^+^: 213.0998, found: 213.1002.

***N*-benzyl-3-phenyl-*N*-(1-(pyridin-2-yl)vinyl)propanamide (a38)**

Following the general procedure A, **a38** was obtained as a yellow liquid (999.2 mg, 2.91 mmol, 58.3 % yield), after column chromatography (silica, PE/EtOAc 5:1).

**^1^H NMR** (400 MHz, CDCl_3_): δ [ppm] = 8.60 (d, *J* = 4.4 Hz, 1H), 7.59 (t, *J* = 7.7 Hz, 1H), 7.25 – 7.04 (m, 12H), 6.23 (s, 1H), 4.84 (s, 1H), 4.68 (s, 2H), 2.97 (t, *J* = 7.6 Hz, 2H), 2.58 (t, *J* = 7.6 Hz, 2H).

**^13^C NMR** (101 MHz, CDCl_3_): δ [ppm] = 172.6, 153.1, 150.0, 145.2, 141.2, 137.5, 137.1, 129.2, 128.6, 128.4, 128.3, 127.4, 126.1, 123.4, 119.5, 118.4, 50.7, 35.6, 31.8.

**HRMS (ESI)** *m/z*: [M+H] ^+^ calculated for C_23_H_23_N_2_O: 343.1805, found: 343.1792.

***N-*benzyl-5-chloro-*N*-(1-(pyridin-2-yl)vinyl)pentanamide (a39)**

Following the general procedure A, **a39** was obtained as a white brown solid (380.6 mg, 1.16 mmol, 23.2% yield), after column chromatography (silica, PE/EtOAc 5:1).

**^1^H NMR** (400 MHz, CDCl_3_): δ [ppm] = 8.63 (d, *J* = 4.4 Hz, 1H), 7.67 (td, *J* = 7.8, 1.6 Hz, 1H), 7.34 – 7.19 (m, 7H), 6.29 (s, 1H), 5.06 (s, 1H), 4.71 (s, 2H), 3.45 (t, *J* = 6.2 Hz, 2H), 2.28 (t, *J* = 6.7 Hz, 2H), 1.83 – 1.65 (m, 4H).

**^13^C NMR** (101 MHz, CDCl_3_): δ [ppm] = 172.8, 153.2, 150.1, 145.4, 137.6, 137.1, 129.1, 128.4, 127.4, 123.5, 119.5, 118.4, 50.7, 44.7, 32.9, 32.1, 22.8.

**HRMS (ESI)** *m/z*: [M+Na] ^+^ calculated for C_19_H_21_ClN_2_ONa^+^: 351.1235, found: 351.1235.

***N*-benzyl*-N*-(1-(pyridin-2-yl)vinyl)hex-5-ynamide (a40)**

Following the general procedure A, **a40** was obtained as a yellow liquid (413.7mg,1.36mmol, 27.1% yield), after column chromatography (silica, PE/EtOAc 5:1).

**^1^H NMR** (400 MHz, CDCl_3_): δ [ppm] = 8.64 (d, *J* = 4.2 Hz, 1H), 7.69 (t, *J* = 7.7 Hz, 1H), 7.29 – 7.22 (m, 7H), 6.32 (s, 1H), 5.09 (s, 1H), 4.72 (s, 2H), 2.41 (t, *J* = 7.2 Hz, 2H), 2.20 (td, *J* = 6.8, 2.3 Hz, 2H), 1.89 – 1.83 (m, 2H), 1.81 (s, 1H).

**^13^C NMR** (101 MHz, CDCl_3_): δ [ppm] = 172.8, 153.2, 150.1, 145.3, 137.7, 137.2, 129.3, 128.5, 127.5, 123.6, 119.7, 118.6, 83.8, 68.9, 50.8, 32.4, 24.2, 18.0.

**HRMS (ESI)** *m/z*: [M+H] ^+^ calculated for C_20_H_21_N_2_O: 305.1648, found: 305.1650.

***N*-benzyl-*N*-(1-(pyridin-2-yl)prop-1-en-1-yl)cyclohexanecarboxamide (a41)**

Following the general procedure A, **a41** was obtained as a yellow liquid (878.9 mg, 2.63 mmol, 52.6% yield), after column chromatography (silica, PE/EtOAc 5:1).

**^1^H NMR** (400 MHz, CDCl_3_): δ [ppm] = 8.66 (d, *J* = 4.1 Hz, 1H), 7.66 (td, *J* = 7.8, 1.7 Hz, 1H), 7.28 – 7.14 (m, 7H), 5.63 (q, *J* = 7.4 Hz, 1H), 4.61 (s, 2H), 2.66 – 2.54 (m, 1H), 1.98 (d, *J* = 7.5 Hz, 3H), 1.72 – 1.54 (m, 7H), 1.25 – 1.17 (m, 1H), 1.15 – 1.02 (m, 2H).

**^13^C NMR** (101 MHz, CDCl_3_): δ [ppm] = 177.5, 155.1, 149.6, 138.3, 138.3, 136.4, 130.7, 129.1, 128.4, 127.2, 122.9, 122.5, 50.7, 42.3, 30.1, 25.9, 25.8, 14.7.

**HRMS (ESI)** *m/z*: [M+Na] ^+^ calculated for C_22_H_26_N_2_ONa^+^: 357.1937, found: 357.1929.

***N*-benzyl-*N*-(1-(pyridin-2-yl)but-1-en-1-yl)cyclohexanecarboxamide (a42)**

Following the general procedure A, **a42** was obtained as a yellow liquid (797.4 mg, 2.29 mmol, 45.8 % yield), after column chromatography (silica, PE/EtOAc 5:1).

**^1^H NMR** (400 MHz, CDCl_3_): δ [ppm] = 8.61 (d, *J* = 4.0 Hz, 1H), 7.62 (t, *J* = 7.1 Hz, 1H), 7.29 – 7.16 (m, 6H), 7.08 (d, *J* = 7.9 Hz, 1H), 6.89 (t, *J* = 7.6 Hz, 1H), 5.34 (d, *J* = 13.7 Hz, 1H), 3.94 (d, *J* = 13.7 Hz, 1H), 2.46 – 2.35 (m, 1H), 1.89 – 1.78 (m, 1H), 1.66 – 1.54 (m, 7H), 1.27 – 1.15 (m, 2H), 1.14 – 1.04 (m, 1H), 1.02 – 0.91 (m, 1H), 0.71 (t, *J* = 7.5 Hz, 3H).

**^13^C NMR** (101 MHz, CDCl_3_): δ [ppm] = 177.4, 153.9, 149.6, 137.4, 130.2, 128.3, 127.6, 122.7, 119.5, 50.2, 41.5, 30.4, 30.0, 25.8, 25.7, 25.7, 21.9, 12.8.

**HRMS (ESI)** *m/z*: [M+Na] ^+^ calculated for C_23_H_28_N_2_ONa^+^: 371.2094, found: 371.2092.

***N*-benzyl*-N*-(3-methyl-1-(pyridin-2-yl)but-1-en-1-yl)cyclohexanecarboxamide (a43)**

Following the general procedure A, **a43** was obtained as a yellow liquid (869.3 mg, 2.40 mmol, 47.9% yield), after column chromatography (silica, PE/EtOAc 10:1).

**^1^H NMR** (400 MHz, CDCl_3_): δ [ppm] = 8.68 – 8.61 (m, 1H), 7.65 (td, *J* = 7.8, 1.6 Hz, 1H), 7.29 – 7.10 (m, 7H), 5.18 (d, *J* = 10.6 Hz, 1H), 4.53 (s, 2H), 3.25 – 3.13 (m, 1H), 2.71 – 2.61 (m, 1H), 1.76 – 1.55 (m, 7H), 1.26 – 1.18 (m, 1H), 1.16 – 1.03 (m, 2H), 0.91 (d, *J* = 6.6 Hz, 6H).

**^13^C NMR** (101 MHz, CDCl_3_): δ [ppm] = 177.3, 155.0, 149.8, 143.9, 138.0, 136.3, 134.8, 129.6, 128.2, 127.2, 122.6, 49.9, 42.3, 30.1, 27.4, 25.9, 25.8, 22.4.

**HRMS (ESI)** *m/z*: [M+Na] ^+^ calculated for C_24_H_30_N_2_ONa^+^: 385.2250, found: 385.2259.

***N*-benzyl-*N*-(1-(pyridin-2-yl)hept-1-en-1-yl)cyclohexanecarboxamide (a44)**

Following the general procedure A, **a44** was obtained as a yellow liquid (460.6 mg, 1.18 mmol, 23.6% yield), after column chromatography (silica, PE/EtOAc 5:1).

**^1^H NMR** (400 MHz, CDCl_3_): δ [ppm] = 8.60 (d, *J* = 4.6 Hz, 1H), 7.60 (td, *J* = 7.8, 1.6 Hz, 1H), 7.30 – 7.14 (m, 6H), 7.06 (d, *J* = 8.0 Hz, 1H), 6.91 (t, *J* = 7.6 Hz, 1H), 5.32 (d, *J* = 13.7 Hz, 1H), 3.96 (d, *J* = 13.7 Hz, 1H), 2.49 – 2.25 (m, 1H), 1.87 – 1.66 (m, 2H), 1.68 – 1.39 (m, 5H), 1.36 – 1.10 (m, 4H), 1.10 – 1.00 (m, 2H), 0.98 – 0.86 (m, 1H), 0.81 (t, *J* = 7.2 Hz, 2H).

**^13^C NMR** (101 MHz, CDCl_3_): δ [ppm] = 177.5, 154.1, 149.8, 137.5, 137.3, 137.0, 134.3, 130.2, 128.3, 127.6, 122.6, 119.4, 50.3, 41.6, 31.9, 30.3, 30.0, 28.7, 28.3, 25.8, 25.7, 25.7, 22.6, 14.1.

**HRMS (ESI)** *m/z*: [M+H] ^+^ calculated for C_26_H_35_N_2_O: 391.2744, found: 391.2739.

***N*-benzyl-*N*-(1-(pyridin-2-yl)hept-1-en-1-yl)-2-(p-tolyl)acetamide (a45)**

Following the general procedure A, **a45** was obtained as a red liquid (1627.4 mg, 3.95 mmol, 79% yield), after column chromatography (silica, PE/EtOAc 5:1).

**^1^H NMR** (400 MHz, CDCl_3_): δ [ppm] = 8.67 (d, *J* = 4.4 Hz, 1H), 7.63 (t, *J* = 7.7 Hz, 1H), 7.26 – 7.16 (m, 7H), 7.12 (d, *J* = 7.9 Hz, 3H), 7.07 (d, *J* = 7.5 Hz, 2H), 5.41 (t, *J* = 7.6 Hz, 1H), 4.59 (s, 2H), 3.74 (s, 2H), 2.44 – 2.34 (m, 2H), 2.30 (s, 3H), 1.28 – 1.11 (m, 6H), 0.82 (t, *J* = 7.0 Hz, 3H).

**^13^C NMR** (101 MHz, CDCl_3_): δ [ppm] = 8.67 δ 172.1, 154.6, 149.8, 138.2, 137.8, 136.8, 136.3, 136.1, 133.1, 129.5 129.2, 129.1, 128.3, 127.3, 123.0, 122.6, 50.6, 40.5, 31.5, 29.0, 28.5, 22.5, 21.2, 14.1.

**HRMS (ESI)** *m/z*: [M+Na] ^+^ calculated for C_28_H_32_N_2_ONa^+^: 435.2407, found: 435.2411.

**2-(Benzyl(1-(pyridin-2-yl)vinyl)amino)-2-oxo-1-phenylethyl acetate (a46)**

Following the general procedure A, **a46** was obtained as a yellow liquid (360.8 mg, 0.98 mmol, 19.6 % yield), after column chromatography (silica, PE/EtOAc 5:1).

**^1^H NMR** (400 MHz, CDCl_3_): δ [ppm] = 8.56 (d, *J* = 2.9 Hz, 1H), 7.62 – 7.19 (m, 13H), 6.34 (s, 1H), 6.08 (s, 1H), 4.74 (s, 2H), 2.14 (s, 3H).

**^13^C NMR** (101 MHz, CDCl_3_): δ [ppm] = 170.9, 168.4, 152.0, 149.9, 143.1, 136.9, 136.8, 134.5, 129.2, 129.14, 129.1, 128.7, 128.5, 127.7, 123.51, 120.8, 120.2, 74.2, 50.8, 21.0.

**HRMS (ESI)** *m/z*: [M+Na] ^+^ calculated for C_24_H_23_N_2_O_3_Na^+^_:_ 409.1522, found: 409.1516.

***N*-methyl-*N*-(1-(pyridin-2-yl)vinyl)dodecanamid (a47)**

Following the general procedure A, **a47** was obtained as a yellow liquid (265.7 mg, 0.84 mmol, 16.8 % yield), after column chromatography (silica, PE/EtOAc 10:1).

**^1^H NMR** (400 MHz, CDCl_3_): δ [ppm] = 8.63 (d, *J* = 3.6 Hz, 1H), 7.69 (t, *J* = 7.3 Hz, 1H), 7.29 – 7.18 (m, 2H), 6.39 (s, 1H), 5.41 (s, 1H), 3.13 (s, 3H), 2.19 (t, *J* = 7.5 Hz, 2H), 1.56 (s, 2H), 1.32 – 1.05 (m, 16H), 0.85 (t, *J* = 6.8 Hz, 3H).

**^13^C NMR** (101 MHz, CDCl_3_): δ [ppm] = 173.9, 153.3, 150.2, 148.0, 137.2, 123.6, 119.6, 116.4, 36.0, 33.8, 32.0, 29.7, 29.6, 29.4, 25.8, 22.8, 14.2.

**HRMS (ESI)** *m/z*: [M+Na] ^+^ calculated for C_20_H_32_N_2_ONa^+^: 339.2407, found: 339.2413.

***N*-benzyl-2-(4-isobutylphenyl)-*N*-(1-(pyridin-2-yl)vinyl)propanamide (a48)**

Following the general procedure A, **a48** was obtained as a yellow liquid (382.3 mg, 0.96 mmol, 19.2% yield), after column chromatography (silica, PE/EtOAc 5:1).

**^1^H NMR** (400 MHz, CDCl_3_): δ [ppm] = 8.62 (d, *J* = 4.1 Hz, 1H), 7.54 (s, 1H), 7.32 – 7.10 (m, 7H), 7.08 (d, *J* = 7.6 Hz, 2H), 6.95 (d, *J* = 7.7 Hz, 2H), 6.31 (s, 1H), 4.83 (s, 1H), 4.70 (s, 2H), 3.85 (d, *J* = 6.1 Hz, 1H), 2.39 (d, *J* = 7.1 Hz, 2H), 1.80 (dt, *J* = 13.4, 6.7 Hz, 1H), 1.42 (d, *J* = 6.9 Hz, 3H), 0.93 – 0.82 (m, 6H).

**^13^C NMR** (101 MHz, CDCl_3_): δ [ppm] = 174.8, 153.3, 152.4, 150.0 145.0, 144.9, 140.1, 139.3, 137.7, 136.9, 129.2, 129.1, 128.4, 127.4, 123.4, 119.7, 118.9, 50.9, 45.1, 43.3, 30.3, 22.5, 20.7.

**HRMS (ESI)** *m/z*: [M+Na] ^+^ calculated for C_27_H_30_N_2_ONa^+^: 421.2250, found: 421.2250.

**2-(4-Benzoylphenyl)-*N*-methyl-*N*-(1-(pyridin-2-yl)vinyl)propanamide (a49)**

Following the general procedure A, **a49** was obtained as a white crystal (655.3 mg, 1.77 mmol, 35.4% yield), after column chromatography (silica, PE/EtOAc 5:1).

**^1^H NMR** (400 MHz, CDCl_3_): δ 8.57 (d, *J* = 3.9 Hz, 1H), 7.70 (d, *J* = 7.5 Hz, 2H), 7.67 – 7.35 (m, 7H), 7.33 (t, *J* = 7.6 Hz, 1H), 7.18 (dd, *J* = 7.0, 5.0 Hz, 1H), 7.08 (d, *J* = 7.6 Hz, 1H), 6.40 (s, 1H), 5.30 (s, 1H), 4.02 – 3.91 (m, 1H), 3.14 (s, 3H), 1.43 (d, *J* = 6.9 Hz, 3H).

**^13^C NMR** (101 MHz, CDCl_3_): δ [ppm] = 196.6, 174.2, 152.9, 150.1, 147.6, 142.2, 137.6, 137.0, 132.5, 131.7, 130.1, 129.6, 128.6, 128.5, 128.4, 123.6, 119.7, 117.0, 43.1, 36.3, 20.5.

**HRMS (ESI)** *m/z*: [M+Na] ^+^ calculated for C_24_H_22_N_2_O_2_Na^+^: 393.1573, found: 393.1576.

***N*-benzyl-*N*-(1-(p-tolyl)vinyl)cyclohexanecarboxamide (a50)**

Following the general procedure A, **a50** was obtained as a yellow liquid (869.6 mg, 2.61 mmol, 52.2% yield), after column chromatography (silica, PE/EtOAc 5:1).

**^1^H NMR** (400 MHz, CDCl_3_): δ [ppm] = 7.33 – 7.15 (m, 9H), 5.55 (s, 1H), 4.78 (s, 1H), 4.62 (s, 2H), 2.61 – 2.52 (m, 1H), 2.38 (s, 3H), 1.71 – 1.57 (m, 7H), 1.26 – 1.17 (m, 1H), 1.14 – 1.01 (m, 2H).

**^13^C NMR** (101 MHz, CDCl_3_): δ [ppm] = 177.0, 146.1, 139.3, 138.2, 133.2, 129.8, 129.1, 128.4, 127.3, 125.9, 113.0, 50.0, 42.0, 30.2, 25.8, 25.7, 21.3.

**HRMS (ESI)** *m/z*: [M+Na] ^+^ calculated for C_23_H_27_NONa^+^: 356.1985, found: 356.1989.

***N*-(1-(pyridin-2-yl)vinyl)isobutyramide (a51)**

Following the general procedure A, **a51** was obtained as a yellow liquid (533.9 mg, 2.81 mmol, 56.2% yield), after column chromatography (silica, PE/EtOAc 10:1).

**^1^H NMR** (400 MHz, CDCl_3_): δ [ppm] = 9.38 (s, 1H), 8.50 (d, *J* = 4.6 Hz, 1H), 7.77 – 7.64 (m, 2H), 7.25 – 7.18 (m, 1H), 6.50 (s, 1H), 5.50 (s, 1H), 2.59 (dt, *J* = 13.8, 6.9 Hz, 1H), 1.25 (d, *J* = 6.9 Hz, 6H).

**^13^C NMR** (101 MHz, CDCl_3_): δ [ppm] = 176.3, 152.2, 147.8, 137.5, 137.0, 123.1, 119.0, 98.9, 37.2, 19.7.

**HRMS (ESI)** *m/z*: [M+Na]^+^ calculated for C_11_H_14_N_2_ONa^+^: 213.0998, found: 213.1003.

## 2.2 Visible-Light-Mediated Diradical Hydrogen Atom Transfer of Enamides

General Procedure B

**Fig. S2.** Visible-Light-Mediated Diradical Hydrogen Atom Transfer of Enamides

**0.2 mmol scale:** The respective enamide (0.2 mmol, 1.0 equiv.), Zn(OTf)_2_ (14.6 mg, 0.04 mmol, 20 mol%) and Ir(dFppy)_2_(dtbbpy)PF_6_ (4.0 mg, 0.004 mmol, 2 mol%) were added to an oven-dried 20 mL glass vial equipped with a stir bar and capped with a septum. After evacuated/backfilled with Argon (3 times), added the DCM (10 mL). After irradiation (λ_max_ = 427 nm, Kessil lamp, 3 vials with one lamp) at rt for 24 hours, added saturated aqueous NaHCO_3_ and extracted with EtOAc. The organic extracts were combined, washed with NaCl (aq.), dried over Na_2_SO_4_, filtered and concentrated in vacuo. The product was purified by column chromatography (silica, PE/EtOAc) to yield the product.

**2-Benzyl-3-methyl-3-(pyridin-2-yl)-2-azaspiro[3.5]nonan-1-one (b1)**

Following the general procedure B, **b1** was obtained as a colorless liquid (50.3 mg, 0.192 mmol, 91% yield), after column chromatography (silica, PE/EtOAc 5:1).

**^1^H NMR** (400 MHz, CDCl_3_): δ [ppm] = 8.60 (d, *J* = 4.4 Hz, 1H), 7.58 (t, *J* = 7.2 Hz, 1H), 7.34 – 7.24 (m, 5H), 7.18 – 7.09 (m, 2H), 4.76 (d, *J* = 15.2 Hz, 1H), 4.20 (d, *J* = 15.2 Hz, 1H), 2.07 – 1.98 (m, 1H), 1.89 – 1.78 (m, 1H), 1.73 – 1.53 (m, 4H), 1.50 (s, 3H), 1.26 – 1.10 (m, 3H), 0.92 – 0.83 (m, 1H).

**^13^C NMR** (101 MHz, CDCl_3_): δ [ppm] = 174.3, 161.5, 149.3, 137.9, 136.0, 128.8, 128.7, 127.6, 122.0, 121.2, 69.8, 62.1, 43.9, 30.3, 29.2, 25.9, 25.6, 23.8, 23.3, 20.0.

**HRMS (ESI)** *m/z*: [M+H] ^+^ calculated for C_21_H_25_N_2_O: 321.1961, found: 321.1955.

**2,3-Dimethyl-3-(pyridin-2-yl)-2-azaspiro[3.3]heptan-1-one (b2)**

Following the general procedure B, **b2** was obtained as a colorless liquid (28.1 mg, 0.130 mmol, 65% yield) after column chromatography (silica, PE/EtOAc 5:1).

**^1^H NMR** (400 MHz, CDCl_3_): δ [ppm] = 8.64 (d, *J* = 4.3 Hz, 1H), 7.68 (td, *J* = 7.8, 1.6 Hz, 1H), 7.23 – 7.14 (m, 2H), 2.77 (s, 3H), 2.35 – 2.25 (m, 2H), 2.06 – 1.88 (m, 2H), 1.67 (s, 3H), 1.65 – 1.57 (m, 1H), 1.44 – 1.33 (m, 1H).

**^13^C NMR** (101 MHz, CDCl_3_): δ [ppm] = 173.5, 160.3, 149.6, 136.7, 122.3, 120.2, 67.1, 64.6, 25.5, 25.2, 24.5, 17.9, 15.6.

**HRMS (ESI)** *m/z*: [M+Na] ^+^ calculated for C_13_H_16_N_2_ONa^+^: 239.1157, found: 239.1158.

2-Benzyl-3-methyl-3-(pyridin-2-yl)-2-azaspiro[3.6]decan-1-one **(b3)**

Following the general procedure B, **b3** was obtained as a white solid (53.5 mg, 0.160 mmol, 80%) after column chromatography (silica, PE/EtOAc 5:1).

**^1^H NMR** (400 MHz, CDCl_3_): δ [ppm] = 8.61 (d, *J* = 4.5 Hz, 1H), 7.59 (td, *J* = 7.7, 1.3 Hz, 1H), 7.39 – 7.23 (m, 5H), 7.21 – 7.07 (m, 2H), 4.76 (d, *J* = 15.2 Hz, 1H), 4.18 (s, 1H), 2.11 – 1.92 (m, 2H), 1.80 – 1.70 (m, 1H), 1.67 – 1.56 (m, 2H), 1.53 (s, 3H), 1.52 – 1.39 (m, 3H), 1.39 – 1.25 (m, 2H), 1.22 – 1.04 (m, 2H).

**^13^C NMR** (101 MHz, CDCl_3_): δ [ppm] = 175.2, 161.7, 149.3, 137.9, 136.1, 128.7, 127.6, 121.9, 121.2, 71.0, 65.2, 44.0, 31.8, 30.7, 30.2, 30.1, 23.8, 23.2, 20.9.

**HRMS (ESI)** *m/z*: [M+Na] ^+^ calculated for C_22_H_26_N_2_ONa^+^: 357.1937, found: 357.1941.

2-Benzyl-7,7-difluoro-3-methyl-3-(pyridin-2-yl)-2-azaspiro[3.5]nonan-1-one **(b4)**

Following the general procedure B, **b4** was obtained as a white solid (48.4 mg, 0.136 mmol, 68%) after column chromatography (silica, PE/EtOAc 5:1).

**^1^H NMR** (400 MHz, CDCl_3_): δ [ppm] = 8.61 (d, *J* = 4.4 Hz, 1H), 7.61 (t, *J* = 7.7 Hz, 1H), 7.37 – 7.24 (m, 5H), 7.23 – 7.16 (m, 1H), 7.16 – 7.07 (m, 1H), 4.80 (d, *J* = 15.1 Hz, 1H), 4.25 (d, *J* = 15.2 Hz, 1H), 2.45 – 2.25 (m, 1H), 2.19 – 2.02 (m, 3H), 1.98 – 1.88 (m, 1H), 1.72 (s, 1H), 1.52 (s, 3H), 1.40 – 1.29 (m, 1H), 1.22 – 1.12 (m, 1H).

**^19^F NMR** (564 MHz, CDCl_3_): δ [ppm] = -95.2 (d, *J* = 229.7 Hz, 1F), -102.0 (d, *J* = 204.0 Hz, 1F).

**^13^C NMR** (101 MHz, CDCl_3_): δ [ppm] = 172.5, 160.7, 149.5, 137.4, 136.4, 128.8, 127.8, 122.4, 121.8 (d, *J* = 240.9 Hz), 120.8, 69.1, 59.9, 44.2, 31.1 (d, *J* = 37.4 Hz), 30.7, 26.7 (d, *J* = 9.9 Hz), 25.7 (d, *J* = 9.4 Hz), 20.2.

**HRMS (ESI)** *m/z*: [M+Na] ^+^ calculated for C_21_H_22_F_2_N_2_ONa^+^: 379.1592, found: 379.1587.

2-Benzyl-3-methyl-3-(pyridin-2-yl)-7-oxa-2-azaspiro[3.5]nonan-1-one **(b5)**

Following the general procedure B, **b5** was obtained as a colorless liquid (58.0 mg, 0.180 mmol, 90%) after column chromatography (silica, PE/EtOAc 5:1).

**^1^H NMR** (400 MHz, CDCl_3_): δ [ppm] = 8.61 (d, *J* = 4.3 Hz, 1H), 7.60 (td, *J* = 7.8, 0.7 Hz, 1H), 7.41 – 7.20 (m, 5H), 7.23 – 7.15 (m, 1H), 7.13 – 7.05 (m, 1H), 4.79 (d, *J* = 15.1 Hz, 1H), 4.22 (d, *J* = 15.1 Hz, 1H), 4.02 – 3.89 (m, 2H), 3.81 – 3.71 (m, 1H), 3.65 – 3.57 (m, 1H), 1.99 – 1.84 (m, 2H), 1.49 (s, 3H), 1.22 – 1.04 (m, 2H).

**^13^C NMR** (101 MHz, CDCl_3_): δ [ppm] = 173.0, 160.6, 149.5, 137.5, 136.3, 128.8, 128.8, 127.7, 122.3, 121.1, 69.1, 65.5, 65.3, 59.3, 44.2, 30.3, 29.5, 19.7.

**HRMS (ESI)** *m/z*: [M+Na] ^+^ calculated for C_20_H_22_N_2_O_2_Na^+^: 345.1573, found: 345.1535.

Benzyl 2-benzyl-1-methyl-3-oxo-1-(pyridin-2-yl)-2,7-diazaspiro[3.5]nonane-7-carboxylate **(b6)**

Following the general procedure B, **b6** was obtained as a colorless liquid (84.7 mg, 0.186 mmol, 93%) after column chromatography (silica, PE/EtOAc 5:1).

**^1^H NMR** (400 MHz, CDCl_3_): δ [ppm] = 8.59 (d, *J* = 4.1 Hz, 1H), 7.60 (t, *J* = 7.2 Hz, 1H), 7.40 – 7.24 (m, 10H), 7.22 – 7.15 (m, 1H), 7.13 – 7.05 (m, 1H), 5.08 (s, 2H), 4.80 (d, *J* = 13.0 Hz, 1H), 4.23 (d, *J* = 12.8 Hz, 1H), 4.11 (s, 1H), 3.77 (s, 1H), 3.48 (s, 1H), 3.25 (s, 1H), 2.03 (s, 1H), 1.78 (s, 1H), 1.48 (s, 3H), 1.24 – 1.11 (m, 1H), 1.00 (s, 1H).

**^13^C NMR** (101 MHz, CDCl_3_): δ [ppm] = 172.7, 160.6, 155.2, 149.5, 137.4, 136.9, 136.3, 128.8, 128.7, 128.1, 127.9, 127.8, 122.3, 120.9, 69.1, 67.2, 59.9, 44.2, 41.8, 41.6, 19.9.

**HRMS (ESI)** *m/z*: [M+Na] ^+^ calculated for C_28_H_29_N_3_O_3_Na^+^: 478.2101, found: 478.2120.

2-Benzyl-3-methyl-3-(pyridin-2-yl)-2-azaspiro[3.4]oct-6-en-1-one **(b7)**

Following the general procedure B, **b7** was obtained as a yellow liquid (32.9 mg, 0.108 mmol, 54%) after column chromatography (silica, PE/EtOAc 5:1).

**^1^H NMR** (400 MHz, CDCl_3_): δ [ppm] = 8.61 (d, *J* = 4.6 Hz, 1H), 7.61 (t, *J* = 7.7 Hz, 1H), 7.36 – 7.21 (m, 5H), 7.21 – 7.10 (m, 2H), 5.71 – 5.60 (m, 1H), 5.57 – 5.44 (m, 1H), 4.78 (d, *J* = 15.2 Hz, 1H), 4.19 (d, *J* = 15.2 Hz, 1H), 2.81 – 2.66 (m, 2H), 2.41 – 2.28 (m, 1H), 1.65 – 1.58 (m, 1H), 1.55 (s, 3H).

**^13^C NMR** (101 MHz, CDCl_3_): δ [ppm] = 174.2, 161.2, 149.5, 137.4, 136.5, 128.9, 128.8, 128.7, 128.4, 127.7, 122.3, 120.8, 69.1, 67.8, 44.2, 37.0, 36.6, 36.0, 21.4.

**HRMS (ESI)** *m/z*: [M+Na] ^+^ calculated for C_20_H_20_N_2_ONa^+^: 327.1468, found: 327.1472.

Acetyl-2-benzyl-3-methyl-3-(pyridin-2-yl)-2,5-diazaspiro[3.4]octan-1-one **(b8)**

Following the general procedure B, **b8** was obtained as a white solid (46.8 mg, 0.134 mmol, 67%) after column chromatography (silica, PE/EtOAc 2:1). dr = 3/1. *Major diastereoisomer:*

**^1^H NMR** (400 MHz, CDCl_3_): δ [ppm] = δ 8.60 (d, *J* = 4.4 Hz, 1H), 7.62 (td, *J* = 7.8, 1.3 Hz, 1H), 7.48 – 7.41 (m, 2H), 7.37 – 7.26 (m, 3H), 7.23 – 7.16 (m, 1H), 7.14 – 7.07 (m, 1H), 4.88 (d, *J* = 15.2 Hz, 1H), 4.33 (d, *J* = 15.3 Hz, 1H), 3.60 – 3.51 (m, 1H), 3.49 – 3.41 (m, 1H), 2.15 (s, 3H), 1.96 – 1.80 (m, 2H), 1.68 – 1.59 (m, 1H), 1.57 (s, 3H), 1.35 – 1.28 (m, 1H).

*Minor diastereoisomer：*

**^1^H NMR** (400 MHz, CDCl_3_): δ [ppm] = 8.48 (d, *J* = 4.3 Hz, 1H), 7.53 – 7.45 (m, 3H), 7.37 – 7.30 (m, 5H), 4.76 (d, *J* = 15.5 Hz, 1H), 4.61 (d, *J* = 15.5 Hz, 1H), 3.37 – 3.28 (m, 2H), 2.15 (s, 3H), 2.09 – 2.01 (m, 2H), 1.70 – 1.65 (m, 1H), 1.54 (s, 3H), 1.42 (d, *J* = 6.1 Hz, 1H).

**^13^C NMR** (101 MHz, CDCl_3_): δ [ppm] = 170.4, 168.6, 160.6, 149.5, 136.6, 136.5, 129.2, 128.9, 128.6, 127.6, 122.3, 121.2, 81.5, 73.3, 49.2, 44.8, 30.3, 23.5, 23.1, 22.2, 18.6.

**HRMS (ESI)** *m/z*: [M+Na] ^+^ calculated for C_21_H_23_N_3_O_2_Na^+^: 372.1682, found: 372.1697.

**1-Benzyl-3,3,4-trimethyl-4-(pyridin-2-yl)azetidin-2-one** **(b9)**

Following the general procedure B, **b9** was obtained as a white solid (53.2 mg, 0.190 mmol, 95%) after column chromatography (silica, PE/EtOAc 2:1).

**^1^H NMR** (400 MHz, CDCl_3_): δ [ppm] = 8.59 (d, *J* = 4.2 Hz, 1H), 7.59 (td, *J* = 7.8, 1.5 Hz, 1H), 7.35 – 7.24 (m, 5H), 7.20 – 7.14 (m, 1H), 7.11 – 7.05 (m, 1H), 4.80 (d, *J* = 15.2 Hz, 1H), 4.27 (d, *J* = 15.2 Hz, 1H), 1.50 (s, 3H), 1.33 (s, 3H), 0.71 (s, 3H).

**^13^C NMR** (101 MHz, CDCl_3_): δ [ppm] = 174.5, 161.7, 149.4, 137.7, 136.3, 128.8, 127.6, 122.0, 120.7, 69.8, 58.3, 44.3, 20.7, 20.1, 18.9.

**HRMS (ESI)** *m/z*: [M+Na] ^+^ calculated for C_18_H_20_N_2_ONa^+^: 303.1468, found: 303.1472.

**1-Benzyl-3,4-dimethyl-3-phenyl-4-(pyridin-2-yl)azetidin-2-one (b10)**

Following the general procedure B, **b10** was obtained as a white solid (58.2 mg, 0.170 mmol, 85%) after column chromatography (silica, PE/EtOAc 2:1). d.r. = 8/1. *Major diastereoisomer:*

**^1^H NMR** (400 MHz, CDCl_3_): δ [ppm] = 8.38 (d, *J* = 4.1 Hz, 1H), 7.33 – 7.25 (m, 5H), 7.15 (td, *J* = 7.8, 1.7 Hz, 1H), 7.02 (d, *J* = 7.0 Hz, 2H), 6.99 – 6.80 (m, 4H), 6.72 (d, *J* = 8.0 Hz, 1H), 4.81 (d, *J* = 15.1 Hz, 1H), 4.15 (d, *J* = 15.1 Hz, 1H), 1.73 (d, *J* = 8.3 Hz, 6H).

*Minor diastereoisomer:*

**^1^H NMR** (400 MHz, CDCl_3_): δ [ppm] = 8.69 (d, *J* = 3.8 Hz, 1H), 7.65 (td, *J* = 7.7, 1.7 Hz, 1H), 7.49 (d, *J* = 7.6 Hz, 2H), 7.26 – 7.19 (m, 5H), 6.92 – 6.88 (m, 5H), 4.91 (d, *J* = 15.3 Hz, 1H), 4.37 (d, *J* = 15.3 Hz, 1H), 1.12 (d, *J* = 11.6 Hz, 6H).

**^13^C NMR** (101 MHz, CDCl_3_): δ [ppm] = 172.2, 160.2, 148.6, 140.0, 137.2, 135.4, 128.9, 128.8, 127.7, 127.1, 126.8, 121.7, 71.1, 68.0, 44.1, 20.9, 19.8.

**HRMS (ESI)** *m/z*: [M+Na] ^+^ calculated for C_23_H_22_N_2_ONa^+^: 365.1624, found: 365.1621.

**1-Benzyl-3-ethyl-4-methyl-3-phenyl-4-(pyridin-2-yl)azetidin-2-one (b11)**

Following the general procedure B, **b11** was obtained as a white solid (66.3 mg, 0.186 mmol, 93%) after column chromatography (silica, PE/EtOAc 2:1). d.r. > 15/1. *Major diastereoisomer:*

**^1^H NMR** (400 MHz, CDCl_3_): δ [ppm] = 8.38 (s, 1H), 7.37 – 7.18 (m, 5H), 7.18 – 7.07 (m, 1H), 7.07 – 6.76 (m, 6H), 6.76 – 6.65 (m, 1H), 4.82 (d, *J* = 15.1 Hz, 1H), 4.04 (d, *J* = 15.1 Hz, 1H), 2.35 – 2.19 (m, 1H), 2.09 – 1.96 (m, 1H), 1.74 (s, 3H), 0.88 (t, *J* = 7.1 Hz, 3H).

**^13^C NMR** (101 MHz, CDCl_3_): δ [ppm] = 171.9, 160.5, 148.6, 137.8, 137.3, 135.4, 128.9, 128.8, 127.9, 127.7, 127.6, 126.1, 121.8, 121.6, 72.2, 71.0, 43.8, 27.8, 19.3, 10.4.

**HRMS (ESI)** *m/z*: [M+Na] ^+^ calculated for C_24_H_24_N_2_ONa^+^: 379.1781, found: 379.1763.

1-benzyl-3-(4-(iodo-l2-methyl)phenyl)-3-isopropyl-4-methyl-4-(pyridin-2-yl)azetidin-2-one **(b12)**

Following the general procedure B, **b12** was obtained as acolorless liquid (79.2 mg, 0.196 mmol, 98%) after column chromatography (silica, PE/EtOAc 5:1). d.r. = 5/1. *Major diastereoisomer:*

**^1^H NMR** (400 MHz, CDCl_3_): δ [ppm] = 8.49 (d, *J* = 4.2 Hz, 1H), 7.45 – 7.10 (m, 9H), 7.04 – 6.88 (m, 2H), 6.71 – 6.62 (m, 1H), 4.74 (d, *J* = 15.2 Hz, 1H), 3.90 (d, *J* = 15.2 Hz, 1H), 2.73 – 2.61 (m, 1H), 1.88 (s, 3H), 1.00 (d, *J* = 6.7 Hz, 3H), 0.91 (d, *J* = 6.6 Hz, 3H).

*Minor diastereoisomer:*

**^1^H NMR** (400 MHz, `CDCl_3_): δ [ppm] = 8.66 (d, *J* = 4.2 Hz, 1H), 7.60 – 7.56 (m, 1H), 7.40 – 7.31 (m, 5H), 7.24 – 7.18 (m, 6H), 4.57 (d, *J* = 15.3 Hz, 1H), 4.38 (d, *J* = 15.3 Hz, 1H), 2.20 – 2.12 (m, 1H), 1.32 (s, 3H), 0.78 (d, *J* = 6.7 Hz, 3H), 0.23 (d, *J* = 6.7 Hz, 3H).

**^13^C NMR** (101 MHz, CDCl_3_): δ [ppm] = 171.6, 160.2, 149.4, 148.6, 137.2, 135.8, 133.3, 131.9, 130.9, 128.8, 128.6, 127.7, 122.3, 122.2, 74.8, 71.8, 43.4, 31.7, 19.5, 19.0, 18.9.

**HRMS (ESI)** *m/z*: [M+Na] ^+^ calculated for C_25_H_25_ClN_2_ONa^+^: 427.1548, found: 427.1548.

**2-Benzyl-3-methyl-3-(4-methylpyridin-2-yl)-2-azaspiro[3.5]nonan-1-one (b13)**

Following the general procedure B, **b13** was obtained as a white solid (56.1 mg, 0.168 mmol, 84%) after column chromatography (silica, PE/EtOAc 5:1).

**^1^H NMR** (400 MHz, CDCl_3_): δ [ppm] = 8.43 (d, *J* = 4.9 Hz, 1H), 7.34 – 7.25 (m, 5H), 6.94 (d, *J* = 4.8 Hz, 1H), 6.83 (s, 1H), 4.62 (d, *J* = 15.1 Hz, 1H), 4.31 (d, *J* = 15.1 Hz, 1H), 2.20 (s, 3H), 2.08 – 2.00 (m, 1H), 1.89 – 1.74 (m, 2H), 1.73 – 1.60 (m, 3H), 1.53 (s, 3H), 1.35 – 1.25 (m, 2H), 1.19 – 1.09 (m, 1H), 0.90 – 0.80 (m, 1H).

**^13^C NMR** (101 MHz, CDCl_3_): δ [ppm] = 174.5, 161.2, 149.0, 147.1, 138.0, 129.0, 128.7, 127.6, 122.9, 122.3, 69.9, 62.1, 43.8, 30.3, 29.1, 25.6, 23.8, 23.4, 21.3, 19.7.

**HRMS (ESI)** *m/z*: [M+Na] ^+^ calculated for C_22_H_26_N_2_ONa^+^: 357.1937, found: 357.1957.

**2-Benzyl-3-(5-fluoropyridin-2-yl)-3-methyl-2-azaspiro[3.5]nonan-1-one (b14)**

following the general procedure B, **b14** was obtained as a white solid (56.8 mg, 0.168 mmol, 84%) after column chromatography (silica, PE/EtOAc 4:1).

**^1^H NMR** (400 MHz, CDCl_3_): δ [ppm] = 8.43 (d, *J* = 2.8 Hz, 1H), 7.38 – 7.20 (m, 6H), 7.11 – 7.02 (m, 1H), 4.69 (d, *J* = 15.1 Hz, 1H), 4.22 (d, *J* = 15.2 Hz, 1H), 2.06 – 1.96 (m, 1H), 1.89 – 1.75 (m, 1H), 1.67 – 1.52 (m, 4H), 1.50 (s, 3H), 1.27 – 1.14 (m, 3H), 0.89 – 0.77 (m, 1H).

^19^F NMR (564 MHz, CDCl_3_): δ [ppm] = -129.7 (s, 1F).

**^13^C NMR** (101 MHz, CDCl_3_): δ [ppm] = 174.1, 158.5 (d, *J* = 230.3 Hz), 157.1, 137.7, 137.4 (d, *J* = 23.1 Hz), 128.8, 127.7, 122.6 (d, *J* = 18.3 Hz), 122.1 (d, *J* = 3.8 Hz), 69.5, 62.2, 43.8, 30.3, 29.1, 25.6, 23.7, 23.2, 19.9.

**HRMS (ESI)** *m/z*: [M+Na] ^+^ calculated for C_21_H_23_FN_2_ONa^+^: 361.1687, found: 361.1689.

**2-Benzyl-3-(4-chloropyridin-2-yl)-3-methyl-2-azaspiro[3.5]nonan-1-one (b15)**

Following the general procedure B, **b15** was obtained as a white solid (52.4 mg, 0.148 mmol, 74%) after column chromatography (silica, PE/EtOAc 5:1).

**^1^H NMR** (400 MHz, CDCl_3_): δ [ppm] = 8.46 (d, *J* = 5.2 Hz, 1H), 7.36 – 7.24 (m, 5H), 7.18 – 7.11 (m, 1H), 7.04 (s, 1H), 4.64 (d, *J* = 15.1 Hz, 1H), 4.29 (d, *J* = 15.1 Hz, 1H), 2.09 – 1.99 (m, 1H), 1.88 – 1.77 (m, 1H), 1.73 – 1.63 (m, 2H), 1.63 – 1.55 (m, 2H), 1.51 (s, 3H), 1.32 – 1.22 (m, 2H), 1.18 – 1.06 (m, 1H), 0.91 – 0.81 (m, 1H).

**^13^C NMR** (101 MHz, CDCl_3_): δ [ppm] = 174.1, 163.5, 150.1, 144.4, 137.6, 128.9, 128.7, 127.8, 122.4, 121.8, 69.8, 62.4, 43.9, 30.4, 29.1, 25.6, 23.8, 23.3, 19.6.

**HRMS (ESI)** *m/z*: [M+H] ^+^ calculated for C_21_H_24_ClN_2_O: 355.1572, found: 355.1572.

**2-Benzyl-3-(5-bromopyridin-2-yl)-3-methyl-2-azaspiro[3.5]nonan-1-one (b16)**

Following the general procedure B, **b16** was obtained as a white solid (65.3 mg, 0.164 mmol, 82%) after column chromatography (silica, PE/EtOAc 4:1).

**^1^H NMR** (400 MHz, CDCl_3_): δ [ppm] = 8.62 (s, 1H), 7.66 (d, *J* = 8.4 Hz, 1H), 7.34 – 7.21 (m, 5H), 6.97 (d, *J* = 8.4 Hz, 1H), 4.67 (d, *J* = 15.1 Hz, 1H), 4.22 (d, *J* = 15.2 Hz, 1H), 2.08 – 1.96 (m, 1H), 1.88 – 1.77 (m, 1H), 1.76 – 1.63 (m, 2H), 1.61 – 1.53 (m, 2H), 1.48 (s, 3H), 1.36 – 1.23 (m, 2H), 1.19 – 1.08 (m, 1H), 0.91 – 0.77 (m, 1H).

**^13^C NMR** (101 MHz, CDCl_3_): δ [ppm] = 174.1, 160.2, 150.3, 138.5, 137.7, 128.9, 128.8, 127.7, 122.6, 119.2, 69.6, 62.3, 43.9, 30.4, 29.1, 25.6, 23.8, 23.3, 19.7.

**HRMS (ESI)** *m/z*: [M+Na] ^+^ calculated for C_21_H_23_BrN_2_ONa^+^: 421.0886, found: 421.0874.

2-Benzyl-3-methyl-3-(5-(trifluoromethyl)pyridin-2-yl)-2-azaspiro[3.5]nonan-1-one **(b17)**

Following the general procedure B, **b17** was obtained as a coiorless liquid (56.7 mg, 0.146 mmol, 73%) after column chromatography (silica, PE/EtOAc 2:1).

**^1^H NMR** (400 MHz, CDCl_3_)): δ [ppm] = 8.84 (s, 1H), 7.76 (d, *J* = 8.3 Hz, 1H), 7.35 – 7.25 (m, 5H), 7.18 (d, *J* = 8.3 Hz, 1H), 4.67 (d, *J* = 15.1 Hz, 1H), 4.28 (d, *J* = 15.2 Hz, 1H), 2.11 – 2.00 (m, 1H), 1.91 – 1.80 (m, 1H), 1.74 – 1.64 (m, 2H), 1.63 – 1.56 (m, 2H), 1.53 (s, 3H), 1.35 – 1.26 (m, 1H), 1.24 – 1.09 (m, 2H), 0.87 – 0.77 (m, 1H).

**^19^F NMR** (564 MHz, CDCl_3_): δ [ppm] = -62.2 (s, 3F).

**^13^C NMR** (101 MHz, CDCl_3_): δ [ppm] = 174.0, 165.8, 146.2, 137.6, 133.1, 128.9, 127.8, 124.0 (dd, *J* = 195.8, 93.4 Hz), 120.9, 69.9, 62.5, 44.0, 30.6, 29.1, 25.6, 23.7, 23.3, 19.7.

**HRMS (ESI)** *m/z*: [M+Na] ^+^ calculated for C_22_H_23_F_3_N_2_ONa^+^: 411.1655, found: 411.1649.

2-Benzyl-3-methyl-3-(pyridin-3-yl)-2-azaspiro[3.5]nonan-1-one **(b18)**

Following the general procedure B, **b18** was obtained as a white solid (43.5mg, 0.136 mmol, 68%) after column chromatography (silica, PE/EtOAc 2:1).

**^1^H NMR** (400 MHz, CDCl_3_): δ [ppm] = 8.51 (d, *J* = 3.9 Hz, 1H), 8.45 (d, *J* = 1.6 Hz, 1H), 7.43 (d, *J* = 7.9 Hz, 1H), 7.33 – 7.20 (m, 6H), 4.68 (d, *J* = 15.1 Hz, 1H), 4.15 (d, *J* = 15.2 Hz, 1H), 2.09 – 2.02 (m, 1H), 1.90 – 1.80 (m, 1H), 1.70 – 1.62 (m, 1H), 1.62 – 1.52 (m, 3H), 1.50 (s, 3H), 1.36 – 1.25 (m, 2H), 1.21 – 1.09 (m, 1H), 0.83 – 0.73 (m, 1H).

**^13^C NMR** (101 MHz, CDCl_3_): δ [ppm] = 173.8, 148.4, 148.0, 137.5, 137.4, 134.5, 128.9, 128.8, 127.82, 123.0, 67.2, 62.1, 43.7, 31.3, 29.1, 25.5, 23.8, 23.3, 20.9.

**HRMS (ESI)** *m/z*: [M+Na] ^+^ calculated for C_21_H_24_N_2_ONa^+^: 361.1687, found: 361.1684.

2-Benzyl-3-methyl-3-(pyridin-4-yl)-2-azaspiro[3.5]nonan-1-one **(b19)**

Following the general procedure B, **b19** was obtained as a white solid (62.8 mg, 0.196 mmol, 98%) after column chromatography (silica, PE/EtOAc 2:1).

**^1^H NMR** (400 MHz, CDCl_3_): δ [ppm] = 8.52 (d, *J* = 5.8 Hz, 2H), 7.35 – 7.24 (m, 5H), 7.02 (d, *J* = 5.9 Hz, 2H), 4.68 (d, *J* = 15.2 Hz, 1H), 4.14 (d, *J* = 15.2 Hz, 1H), 2.07 – 1.99 (m, 1H), 1.91 – 1.80 (m, 1H), 1.68 – 1.48 (m, 4H), 1.44 (s, 3H), 1.40 – 1.26 (m, 2H), 1.19 – 1.08 (m, 1H), 0.83 – 0.70 (m, 1H).

**^13^C NMR** (101 MHz, CDCl_3_): δ [ppm] = 173.7, 151.0, 149.8, 137.4, 128.9, 128.8, 127.8, 121.7, 68.1, 62.0, 43.8, 30.9, 29.1, 25.5, 23.8, 23.3, 20.4.

**HRMS (ESI)** *m/z*: [M+Na] ^+^ calculated for C_21_H_24_N_2_ONa^+^: 343.1781, found: 343.1788.

2-Benzyl-3-(isoquinolin-1-yl)-3-methyl-2-azaspiro[3.5]nonan-1-one **(b20)**

Following the general procedure B, **b20** was obtained as a white solid (48.9 mg, 0.132 mmol, 66%) after column chromatography (silica, PE/EtOAc 2:1).

**^1^H NMR** (400 MHz, CDCl_3_): δ [ppm] = 8.47 (d, *J* = 5.6 Hz, 1H), 7.84 (d, *J* = 8.1 Hz, 1H), 7.79 (d, *J* = 8.3 Hz, 1H), 7.69 – 7.61 (m, 1H), 7.58 – 7.53 (m, 2H), 7.47 (d, *J* = 7.3 Hz, 2H), 7.36 – 7.28 (m, 2H), 7.26 – 7.21 (m, 1H), 5.14 (d, *J* = 15.2 Hz, 1H), 4.86 (d, *J* = 15.2 Hz, 1H), 2.45 – 2.33 (m, 1H), 2.14 – 2.04 (m, 1H), 1.91 – 1.81 (m, 2H), 1.78 – 1.72 (m, 1H), 1.53 (s, 3H), 1.45 – 1.35 (m, 1H), 1.30 – 1.20 (m, 2H), 1.18 – 1.06 (m, 2H).

**^13^C NMR** (101 MHz, CDCl_3_): δ [ppm] = 178.1, 167.7, 141.4, 139.1, 137.0, 129.6, 129.1, 128.5, 128.1, 127.2, 126.6, 126.2, 120.3, 71.1, 61.0, 45.5, 31.6, 29.2, 25.9, 23.9, 23.6, 22.9.

**HRMS (ESI)** *m/z*: [M+Na] ^+^ calculated for C_25_H_26_N_2_ONa^+^: 393.1937, found: 393.1942.

**2,3-Dimethyl-3-(pyridin-2-yl)-2-azaspiro[3.5]nonan-1-one (b21)**

Following the general procedure B, **b21** was obtained as a white solid (45.4 mg, 0.186 mmol, 93%) after column chromatography (silica, PE/EtOAc 3:1).

**^1^H NMR** (400 MHz, CDCl_3_): δ [ppm] = 8.60 (d, *J* = 4.5 Hz, 1H), 7.67 (td, *J* = 7.8, 1.5 Hz, 1H), 7.25 – 7.14 (m, 2H), 2.85 (s, 3H), 2.08 – 1.98 (m, 1H), 1.88 – 1.71 (m, 2H), 1.70 (s, 3H), 1.67 – 1.61 (m, 1H), 1.57 – 1.46 (m, 2H), 1.27 – 1.13 (m, 3H), 0.94 – 0.86 (m, 1H).

**^13^C NMR** (101 MHz, CDCl_3_): δ [ppm] = 174.3, 161.4, 149.5, 136.2, 122.0, 120.7, 68.3, 62.1, 30.4, 28.8, 25.6, 24.3, 23.7, 23.2, 18.3.

**HRMS (ESI)** *m/z*: [M+Na] ^+^ calculated for C_15_H_20_N_2_ONa^+^: 267.1468, found: 267.1464.

2-(4-Fluorobenzyl)-3-methyl-3-(pyridin-2-yl)-2-azaspiro[3.5]nonan-1-one **(b22)**

Following the general procedure B, **b22** was obtained as a yellow solid (63.6 mg, 0.188 mmol, 94%) after column chromatography (silica, PE/EtOAc 5:1).

**^1^H NMR** (400 MHz, CDCl_3_): δ [ppm] = 8.59 (d, *J* = 4.4 Hz, 1H), 7.59 (t, *J* = 7.6 Hz, 1H), 7.35 – 7.22 (m, 2H), 7.16 (dd, *J* = 7.2, 5.1 Hz, 1H), 7.10 (d, *J* = 7.9 Hz, 1H), 7.02 – 6.90 (m, 2H), 4.71 (d, *J* = 15.2 Hz, 1H), 4.18 (d, *J* = 15.2 Hz, 1H), 2.05 – 1.95 (m, 1H), 1.87 – 1.76 (m, 1H), 1.73 – 1.51 (m, 4H), 1.50 (s, 3H), 1.27 – 1.10 (m, 3H), 0.94 – 0.80 (m, 1H).

**^19^F NMR** (564 MHz, CDCl_3_): δ [ppm] = -115.0 (s, 1F).

**^13^C NMR** (101 MHz, CDCl_3_): δ [ppm] = 174.2, 162.3 (d, *J* = 245.9 Hz), 161.3, 149.3, 136.0, 133.7, 130.5 (d, *J* = 8.1 Hz), 122.1, 121.1, 115.6 (d, *J* = 21.5 Hz), 69.7, 62.1, 43.2, 30.3, 29.1, 25.6, 23.8, 23.3, 20.0.

**HRMS (ESI)** *m/z*: [M+Na] ^+^ calculated for C_21_H_23_FN_2_ONa^+^: 361.1687, found: 361.1684.

2-(4-Chlorobenzyl)-3-methyl-3-(pyridin-2-yl)-2-azaspiro[3.5]nonan-1-one **(b23)**

Following the general procedure B, **b23** was obtained as a white solid (62.3 mg, 0.176 mmol, 88%) after column chromatography (silica, PE/EtOAc 5:1).

**^1^H NMR** (400 MHz, CDCl_3_): δ [ppm] = 8.60 (d, *J* = 4.0 Hz, 1H), 7.66 – 7.57 (m, 1H), 7.35 – 7.24 (m, 4H), 7.21 – 7.10 (m, 2H), 4.73 (d, *J* = 15.3 Hz, 1H), 4.17 (d, *J* = 15.3 Hz, 1H), 2.06 – 1.98 (m, 1H), 1.97 – 1.74 (m, 2H), 1.71 – 1.56 (m, 3H), 1.51 (s, 3H), 1.29 – 1.10 (m, 3H), 0.94 – 0.83 (m, 1H).

**^13^C NMR** (101 MHz, CDCl_3_): δ [ppm] = 174.2, 161.3, 149.3, 136.5, 136.1, 133.5, 130.2, 128.9, 122.1, 121.1, 69.8, 62.2, 43.3, 30.3, 29.1, 25.6, 23.8, 23.3, 20.0.

**HRMS (ESI)** *m/z*: [M+Na] ^+^ calculated for C_21_H_23_ClN_2_ONa^+^: 377.1391, found: 377.1405.

2-(4-Bromobenzyl)-3-methyl-3-(pyridin-2-yl)-2-azaspiro[3.5]nonan-1-one **(b24)**

Following the general procedure B, **b24** was obtained as a yellow solid (69.3 mg, 0.174 mmol, 87%) after column chromatography (silica, PE/EtOAc 5:1).

**^1^H NMR** (400 MHz, CDCl_3_): δ [ppm] = 8.60 (d, *J* = 4.1 Hz, 1H), 7.60 (td, *J* = 7.8, 1.6 Hz, 1H), 7.43 (d, *J* = 8.3 Hz, 2H), 7.21 (d, *J* = 8.3 Hz, 2H), 7.19 – 7.08 (m, 2H), 4.71 (d, *J* = 15.3 Hz, 1H), 4.15 (d, *J* = 15.3 Hz, 1H), 2.05 – 1.98 (m, 1H), 1.86 – 1.76 (m, 1H), 1.73 – 1.52 (m, 4H), 1.50 (s, 3H), 1.27 – 1.13 (m, 3H), 0.91 – 0.80 (m, 1H).

**^13^C NMR** (101 MHz, CDCl_3_): δ [ppm] = 174.2, 161.3, 149.3, 137.0, 136.1, 131.9, 130.5, 122.1, 121.1, 69.8, 62.2, 43.4, 30.3, 29.1, 25.6, 23.8, 23.3, 20.0.

**HRMS (ESI)** *m/z*: [M+Na] ^+^ calculated for C_21_H_23_BrN_2_ONa^+^: 421.0886 , found: 421.0879.

2-(3,4-Difluorobenzyl)-3-methyl-3-(pyridin-2-yl)-2-azaspiro[3.5]nonan-1-one **(b25)**

Following the general procedure B, **b25** was obtained as a white solid (48.4 mg, 0.136 mmol, 68%) after column chromatography (silica, PE/EtOAc 2:1).

**^1^H NMR** (400 MHz, CDCl_3_): δ [ppm] = 8.60 (d, *J* = 4.7 Hz, 1H), 7.61 (t, *J* = 7.7 Hz, 1H), 7.27 – 7.15 (m, 2H), 7.14 – 7.00 (m, 3H), 4.70 (d, *J* = 15.4 Hz, 1H), 4.16 (d, *J* = 15.4 Hz, 1H), 2.06 – 1.99 (m, 1H), 1.88 – 1.78 (m, 1H), 1.77 – 1.55 (m, 4H), 1.52 (s, 3H), 1.29 – 1.10 (m, 3H), 0.90 – 0.80 (m, 1H).

**^19^F NMR** (564 MHz, CDCl_3_): δ [ppm] = -137.1 – -137.2 (m, 1F), -139.4 – -139.6 (m, 1F).

**^13^C NMR** (101 MHz, CDCl_3_): δ [ppm] = 174.2, 169.3 (d, *J* = 249.5 Hz), 165.9 (d, *J* = 232.8 Hz), 161.0, 149.2, 136.3, 124.8, 122.2, 121.1, 117.83 117.7, 117.6, 117.4, 69.9, 62.2, 43.1 30.3, 29.1, 25.5, 23.7, 23.3, 20.1.

**HRMS (ESI)** *m/z*: [M+H] ^+^ calculated for C_21_H_23_F_2_N_2_O: 357.1773, found: 357.1770.

2-(3-Fluoro-4-methoxybenzyl)-3-methyl-3-(pyridin-2-yl)-2-azaspiro[3.5]nonan-1-one **(b26)**

Following the general procedure B, **b26** was obtained as a white solid (49.3 mg, 0.134 mmol, 67%) after column chromatography (silica, PE/EtOAc 2:1).

**^1^H NMR** (400 MHz, CDCl_3_): δ [ppm] = 8.60 (d, *J* = 4.7 Hz, 1H), 7.60 (td, *J* = 7.7, 1.5 Hz, 1H), 7.20 – 7.00 (m, 4H), 6.97 – 6.86 (m, 1H), 4.68 (d, *J* = 15.2 Hz, 1H), 4.12 (d, *J* = 15.2 Hz, 1H), 3.87 (s, 3H), 2.07 – 1.97 (m, 1H), 1.87 – 1.77 (m, 1H), 1.68 – 1.55 (m, 4H), 1.52 (s, 3H), 1.28 – 1.11 (m, 3H), 0.91 – 0.82 (m, 1H).

**^13^C NMR** (101 MHz, CDCl_3_): δ [ppm] = 174.2, 161.4, 152.3 (d, *J* = 246.5 Hz), 149.3, 136.0, 130.9, 124.6, 122.1, 121.1, 116.7, 116.5, 113.5, 69.7, 62.2, 56.5, 43.1, 30.3, 29.2, 25.6, 23.8, 23.3, 20.0.

**^19^F NMR** (564 MHz, CDCl_3_): δ [ppm] = -134.64 (1F).

**HRMS (ESI)** *m/z*: [M+Na] ^+^ calculated for C_22_H_25_FN_2_O_2_Na^+^: 391.1792, found: 391.1791.

2-Benzoyl-3-methyl-3-(pyridin-2-yl)-2-azaspiro[3.5]nonan-1-one **(b27)**

Following the general procedure B, **b27** was obtained as a white solid (47.5 mg, 0.142 mmol, 71%) after column chromatography (silica, PE/EtOAc 3:1).

**^1^H NMR** (400 MHz, CDCl_3_): δ [ppm] = 8.60 (t, *J* = 5.4 Hz, 1H), 7.95 (t, *J* = 7.0 Hz, 2H), 7.71 – 7.53 (m, 2H), 7.53 – 7.40 (m, 2H), 7.30 – 7.25 (m, 1H), 7.22 – 7.12 (m, 1H), 2.24 – 2.16 (m, 1H), 2.08 (d, *J* = 6.4 Hz, 2H), 1.76 (dd, *J* = 5.1, 1.3 Hz, 2H), 1.58 – 1.48 (m, 3H), 1.46 – 1.37 (m, 1H), 1.33 – 1.24 (m, 2H), 1.21 – 1.12 (m, 1H), 1.07 – 0.96 (m, 1H).

**^13^C NMR** (101 MHz, CDCl_3_): δ [ppm] = 171.0, 167.3, 159.6, 149.1, 136.3, 133.6, 132.9, 129.6, 128.1, 122.8, 120.7, 69.1, 60.5, 30.5, 28.9, 25.4, 23.5, 23.2, 20.1.

**HRMS (ESI)** *m/z*: [M+Na] ^+^ calculated for C_21_H_23_N_3_O_2_Na^+^: 357.1573, found: 357.157.

**1-(Hex-5-en-1-yl)-3,3,4-trimethyl-4-(pyridin-2-yl)azetidin-2-one** **(b28)**

Following the general procedure B, **b28** was obtained as a white solid (42.5 mg, 0.156 mmol, 78%) after column chromatography (silica, PE/EtOAc 2:1).

**^1^H NMR** (400 MHz, CDCl_3_): δ [ppm] = 8.60 (d, *J* = 4.3 Hz, 1H), 7.68 (t, *J* = 7.7 Hz, 1H), 7.26 – 7.17 (m, 2H), 5.83 – 5.73 (m, 1H), 5.05 – 4.93 (m, 2H), 3.53 – 3.42 (m, 1H), 3.12 – 3.01 (m, 1H), 2.11 – 2.05 (m, 2H), 1.87 – 1.78 (m, 1H), 1.75 (s, 3H), 1.48 – 1.39 (m, 2H), 1.34 (s, 3H), 1.20 – 1.13 (m, 1H), 0.68 (s, 3H).

**^13^C NMR** (101 MHz, CDCl_3_): δ [ppm] = 174.6, 161.9, 149.5, 138.5, 136.3, 122.1, 120.5, 115.0, 69.1, 57.9, 40.9, 33.4, 28.7, 26.8, 20.5, 20.0, 19.0.

**HRMS (ESI)** *m/z*: [M+Na] ^+^ calculated for C_17_H_24_N_2_ONa^+^: 295.1781, found: 295.1778.

1-Benzyl-4-methyl-3-phenyl-4-(pyridin-2-yl)azetidin-2-one **(b29)**

Following the general procedure B, **b29** was obtained as a white solid (39.4 mg, 0.120 mmol, 60%) after column chromatography (silica, PE/EtOAc 2:1). d.r. = 2/1. *Major diastereoisomer:*

**^1^H NMR** (400 MHz, CDCl_3_): δ [ppm] = 8.39 (d, *J* = 4.6 Hz, 1H), 7.36 – 7.25 (m, 7H), 7.01 – 6.89 (m, 5H), 6.83 (d, *J* = 8.0 Hz, 1H), 4.88 (d, *J* = 15.1 Hz, 1H), 4.52 (s, 1H), 4.34 (d, *J* = 15.1 Hz, 1H), 1.80 (s, 3H).

*Minor diastereoisomer:*

**^1^H NMR** (400 MHz, CDCl_3_): δ [ppm] = 8.65 (d, *J* = 4.4 Hz, 1H), 7.66 (t, *J* = 7.5 Hz, 1H), 7.25 – 7.18 (m, 5H), 7.08 – 6.99 (m, 7H), 4.82 (d, *J* = 15.2 Hz, 1H), 4.65 (s, 1H), 4.16 (d, *J* = 15.1 Hz, 1H), 1.15 (s, 3H).

**^13^C NMR** (101 MHz, CDCl_3_): δ [ppm] = 168.0, 158.7, 149.7, 148.8, 137.0, 135.6, 133.3, 129.0, 128.8, 128.6, 128.0, 127.8, 127.0, 121.9, 121.7, 68.5, 68.5, 44.9, 24.3.

**HRMS (ESI)** *m/z*: [M+Na] ^+^ calculated for C_22_H_20_N_2_ONa^+^: 351.1468, found: 351.1464.

1-Benzyl-4-methyl-4-(pyridin-2-yl)-3-(p-tolyl)azetidin-2-one **(b30)**

Following the general procedure B, **b30** was obtained as a white solid (53.4 mg, 0.156 mmol, 78%) after column chromatography (silica, PE/EtOAc 2:1). d.r. = 2/1. *Major diastereoisomer:*

**^1^H NMR** (400 MHz, CDCl_3_): δ [ppm] = 8.65 (d, *J* = 4.3 Hz, 1H), 7.66 (t, *J* = 7.7 Hz, 1H), 7.31 – 7.19 (m, 8H), 7.14 (s, 3H), 4.82 (d, *J* = 15.1 Hz, 1H), 4.59 (s, 1H), 4.15 (d, *J* = 15.2 Hz, 1H), 2.33 (s, 3H), 1.14 (s, 3H).

*Minor diastereoisomer:*

^1^H NMR (400 MHz, `CDCl_3_): δ [ppm] = 8.40 (d, *J* = 4.7 Hz, 1H), 7.37 – 7.28 (m, 6H), 6.96 – 6.90 (m, 1H), 6.87 – 6.79 (m, 5H), 4.87 (d, *J* = 15.1 Hz, 1H), 4.48 (s, 1H), 4.34 (d, *J* = 15.1 Hz, 1H), 2.14 (s, 3H), 1.79 (s, 3H).

^1^**^3^C NMR** (101 MHz, CDCl_3_): δ [ppm] = 168.6, 161.6, 149.6, 137.3, 137.0, 136.9, 130.5, 129.4, 128.87, 128.8, 128.7, 127.6, 122.6, 120.0, 66.9, 66.3, 44.7, 21.3, 20.0.

**HRMS (ESI)** *m/z*: [M+H] ^+^ calculated for C_23_H_23_N_2_O: 343.1805, found: 343.1813.

**1-Benzyl-3-(4-fluorophenyl)-4-methyl-4-(pyridin-2-yl)azetidin-2-one (b31)**

Following the general procedure B, **b31** was obtained as a yellow liquid (51.2 mg, 0.148 mmol, 74%) after column chromatography (silica, PE/EtOAc 3:1). d.r. = 2/1. *Major diastereoisomer:*

**^1^H NMR** (400 MHz, CDCl_3_): δ [ppm] = 8.40 (d, *J* = 4.2 Hz, 1H), 7.37 – 7.26 (m, 6H), 6.98 – 6.90 (m, 3H), 6.83 (d, *J* = 8.0 Hz, 1H), 6.75 – 6.64 (m, 2H), 4.88 (d, *J* = 15.1 Hz, 1H), 4.48 (s, 1H), 4.32 (d, *J* = 15.1 Hz, 1H), 1.79 (s, 3H).

*Minor diastereoisomer:*

**^1^H NMR** (400 MHz, CDCl_3_): δ [ppm] = 8.65 (d, *J* = 4.4 Hz, 1H), 7.66 (td, *J* = 7.8, 1.6 Hz, 1H), 7.27 – 7.22 (m, 9H), 7.03 (t, *J* = 8.6 Hz, 2H), 4.79 (d, *J* = 15.1 Hz, 1H), 4.63 (s, 1H), 4.16 (d, *J* = 15.1 Hz, 1H), 1.15 (s, 3H).

**^19^F NMR** (564 MHz, CDCl_3_): δ [ppm] = -115.3 (s, 1F).

**^13^C NMR** (101 MHz, CDCl_3_): δ [ppm] = 167.8, 161.8 (d, *J* = 246.0 Hz), 158.5, 148.9, 136.8, 135.9, 130.3, 130.2, 129.0, 128.8, 127.9, 122.1, 121.6, 115.1, 114.8, 68.5, 67.7, 44.9, 24.8.

**HRMS (ESI)** *m/z*: [M+Na] ^+^ calculated for C_22_H_19_FN_2_ONa^+^: 369.1374, found: 369.1367.

**1-Benzyl-3-(4-chlorophenyl)-4-methyl-4-(pyridin-2-yl)azetidin-2-one (b32)**

Following the general procedure B, **b32** was obtained as a yellow solid (50.7 mg, 0.14 mmol, 70%) after column chromatography (silica, PE/EtOAc 2:1). d.r. = 2/1. *Major diastereoisomer:*

**^1^H NMR** (400 MHz, CDCl_3_): δ [ppm] = 8.40 (d, *J* = 4.4 Hz, 1H), 7.34 – 7.24 (m, 6H), 7.02 – 6.89 (m, 5H), 6.83 (d, *J* = 8.0 Hz, 1H), 4.87 (d, *J* = 15.1 Hz, 1H), 4.47 (s, 1H), 4.30 (d, *J* = 15.1 Hz, 1H), 1.79 (s, 3H).

*Minor diastereoisomer:*

**^1^H NMR** (400 MHz, CDCl_3_): δ [ppm] = 8.66 (d, *J* = 4.2 Hz, 1H), 7.66 (d, *J* = 1.5 Hz, 1H), 7.36 – 7.18 (m, 11H), 4.79 (d, *J* = 15.1 Hz, 1H), 4.65 (s, 1H), 4.17 (d, *J* = 15.2 Hz, 1H), 1.15 (s, 3H).

**^13^C NMR** (101 MHz, CDCl_3_): δ [ppm] = 167.9, 161.2, 149.8, 136.9, 136.8, 133.7, 132.3, 130.3, 129.0, 128.8, 128.7, 127.7, 122.7, 120.0, 66.3, 66.1, 44.8, 20.2.

**HRMS (ESI)** *m/z*: [M+Na] ^+^ calculated for C_22_H_19_ClN_2_ONa^+^: 385.1078, found: 385.1091.

**1-Benzyl-3-(4-methoxyphenyl)-4-methyl-4-(pyridin-2-yl)azetidin-2-one (b33)**

Following the general procedure B, **b33** was obtained as a white solid (57.3 mg, 0.160 mmol, 80%) after column chromatography (silica, PE/EtOAc 2:1). d.r. = 2/1. *Major diastereoisomer:*

**^1^H NMR** (400 MHz, CDCl_3_): δ [ppm] = δ 8.40 (d, *J* = 4.7 Hz, 1H), 7.42 – 7.26 (m, 6H), 6.98 – 6.79 (m, 4H), 6.54 (d, *J* = 8.5 Hz, 2H), 4.87 (d, *J* = 15.1 Hz, 1H), 4.46 (s, 1H), 4.34 (d, *J* = 15.1 Hz, 1H), 3.65 (s, 3H), 1.78 (s, 3H). *Minor diastereoisomer:*

**^1^H NMR** (400 MHz, cdcl_3_) δ 8.64 (d, *J* = 4.2 Hz, 1H), 7.65 (t, *J* = 7.8 Hz, 1H), 7.36 – 7.28 (m, 7H), 7.17 (d, *J* = 8.5 Hz, 2H), 6.96 – 6.89 (m, 2H), 4.81 (d, *J* = 15.2 Hz, 1H), 4.58 (s, 1H), 4.15 (d, *J* = 15.1 Hz, 1H), 3.79 (s, 3H), 1.14 (s, 3H)

**^13^C NMR** (101 MHz, CDCl_3_): δ [ppm] = 168.3, 158.5, 148.4, 143.3, 137.0, 130.7, 129.8, 129.0, 128.8, 127.8, 125.3, 122.1, 121.9, 113.5, 68.5, 68.1, 55.2, 44.9, 24.2.

**HRMS (ESI)** *m/z*: [M+Na] ^+^ calculated for C_23_H_22_N_2_O_2_Na^+^: 381.1573, found: 381.1553.

1-Benzyl-4-methyl-4-(pyridin-2-yl)-3-(thiophen-2-yl)azetidin-2-one **(b34)**

Following the general procedure B, **b34** was obtained as a white solid (48.1 mg, 0.144 mmol, 72%) after column chromatography (silica, PE/EtOAc 2:1). d.r. = 2/1. *Major diastereoisomer:*

**^1^H NMR** (400 MHz, CDCl_3_): δ [ppm] = 8.47 (d, *J* = 4.4 Hz, 1H), 7.37 – 7.26 (m, 7H), 7.04 – 7.01 (m, 1H), 6.96 (d, *J* = 6.5 Hz, 2H), 6.71 – 6.67 (m, 1H), 4.89 (d, *J* = 15.1 Hz, 1H), 4.65 (s, 1H), 4.31 (d, *J* = 15.1 Hz, 1H), 1.77 (s, 3H).

*Minor diastereoisomer:*

**^1^H NMR** (400 MHz, CDCl_3_): δ [ppm] = 8.65 (d, *J* = 4.4 Hz, 1H), 7.65 (d, *J* = 7.6 Hz, 1H), 7.40 – 7.33 (m, 5H), 7.06 – 7.02 (m, 3H), 6.77 – 6.71 (m, 2H), 4.82 (s, 1H), 4.80 (d, *J* = 15.2 Hz, 1H), 4.17 (d, *J* = 15.2 Hz, 1H), 1.25 (s, 3H).

**^13^C NMR** (101 MHz, CDCl_3_): δ [ppm] = 167.0, 158.3, 149.8, 149.0, 135.8, 129.0, 128.8, 128.8, 128.7, 127.9, 127.4, 127.3, 126.7, 125.5, 122.3, 121.7, 63.7, 45.1, 23.8, 19.6.

**HRMS (ESI)** *m/z*: [M+Na] ^+^ calculated for C_20_H_18_N_2_OSNa^+^: 357.1032, found: 357.1041.

**1-Benzyl-3-methoxy-4-methyl-4-(pyridin-2-yl)azetidin-2-one (b35)**

Following the general procedure B, **b35** was obtained as a white solid (25.4 mg, 0.090 mmol, 45%) after column chromatography (silica, PE/EtOAc 2:1). d.r. = 3/1. *Major diastereoisomer:*

**^1^H NMR** (400 MHz, CDCl_3_): δ [ppm] = 8.59 (d, *J* = 4.7 Hz, 1H), 7.65 – 7.57 (m, 1H), 7.24 – 7.15 (m, 7H), 4.65 (d, *J* = 15.0 Hz, 1H), 4.37 (s, 1H), 4.15 (d, *J* = 15.0 Hz, 1H), 3.10 (s, 3H), 1.69 (s, 3H). *Minor diastereoisomer:*

**^1^H NMR** (400 MHz, CDCl_3_): δ [ppm] = 8.60 (d, *J* = 4.0 Hz, 1H), 7.64 – 7.60 (m, 1H), 7.34 – 7.24 (m, 7H), 4.65 (d, *J* = 15.1 Hz, 1H), 4.50 (s, 1H), 4.06 (d, *J* = 15.1 Hz, 1H), 3.49 (s, 3H), 1.52 (s, 3H).

**^13^C NMR** (101 MHz, CDCl_3_): δ [ppm] = 165.6, 156.7, 148.1, 135.0, 127.9, 127.8, 127.8, 127.6, 126.9, 121.7, 121.6, 119.2, 91.8, 58.4, 57.6, 43.1, 20.7.

**HRMS (ESI)** *m/z*: [M+Na] ^+^ calculated for C_17_H_18_N_2_O_2_Na^+^: 305.1260, found: 305.1268.

1-Benzyl-3-(benzyloxy)-4-methyl-4-(pyridin-2-yl)azetidin-2-one **(b36)**

Following the general procedure B, **b36** was obtained as colorless liquid (57.3 mg, 0.160 mmol, 80%) after column chromatography (silica, PE/EtOAc 3:1). d.r. = 3/1. *Major diastereoisomer:*

**^1^H NMR** (400 MHz, CDCl_3_): δ [ppm] = 8.59 (d, *J* = 4.2 Hz, 1H), 7.57 (td, *J* = 7.8, 1.7 Hz, 1H), 7.31 – 7.16 (m, 10H), 6.95 – 6.88 (m, 2H), 4.64 (d, *J* = 15.0 Hz, 1H), 4.56 (s, 1H), 4.31 (d, *J* = 11.4 Hz, 1H), 4.23 (d, *J* = 11.4 Hz, 1H), 4.16 (d, *J* = 15.0 Hz, 1H), 1.67 (s, 3H).

**^13^C NMR** (101 MHz, CDCl_3_): δ [ppm] = 166.6, 157.8, 149.0, 136.6, 136.3, 135.9, 128.9, 128.7, 128.3, 128.0, 127.8, 123.0, 122.6, 90.6, 72.5, 69.4, 44.2, 21.4.

**HRMS (ESI)** *m/z*: [M+Na] ^+^ calculated for C_23_H_22_N_2_O_2_Na^+^: 381.1573, found: 381.1574.

**1,3,4-Trimethyl-4-(pyridin-2-yl)azetidin-2-one (b37)**

Following the general procedure B, **b37** was obtained as a white solid (17.5 mg, 0.092 mmol, 46%) after column chromatography (silica, PE/EtOAc 2:1). d.r. = 3/1. *Major diastereoisomer:*

**^1^H NMR** (400 MHz, CDCl_3_): δ [ppm] = (d, *J* = 4.3 Hz, 1H), 7.71 (t, *J* = 7.7 Hz, 1H), 7.26 – 7.18 (m, 2H), 3.23 – 3.17 (m, 1H), 2.88 (s, 3H), 1.84 (s, 3H), 0.75 (d, *J* = 7.5 Hz, 3H).

*Minor diastereoisomer:*

**^1^H NMR** (400 MHz, CDCl_3_): δ [ppm] = 8.60 (d, *J* = 4.5 Hz, 1H), 7.34 – 7.26 (m, 3H), 3.20 (d, *J* = 7.5 Hz, 1H), 2.82 (s, 3H), 1.68 (s, 3H), 1.30 (d, *J* = 7.6 Hz, 3H).

**^13^C NMR** (101 MHz, CDCl_3_): δ [ppm] = 171.0, 159.9, 149.7, 136.5, 122.3, 120.7, 57.5, 29.83, 25.1, 22.0, 10.6.

**HRMS (ESI)** *m/z*: [M+Na] ^+^ calculated for C_11_H_14_N_2_ONa^+^: 213.0998, found: 213.1003.

1,3-Dibenzyl-4-methyl-4-(pyridin-2-yl)azetidin-2-one **(b38)**

Following the general procedure B, **b38** was obtained as a white solid (27.4 mg, 0.08 mmol, 40%) after column chromatography (silica, PE/EtOAc 2:1). d.r. = 5/1. *Major diastereoisomer:*

**^1^H NMR** (400 MHz, CDCl_3_): δ [ppm] = 8.61 (d, *J* = 4.3 Hz, 1H), 7.61 – 7.53 (m, 1H), 7.26 – 7.19 (m, 7H), 7.14 – 7.09 (m, 3H), 6.80 (d, *J* = 6.6 Hz, 2H), 4.78 (d, *J* = 15.1 Hz, 1H), 4.07 (d, *J* = 15.1 Hz, 1H), 3.57 – 3.46 (m, 1H), 2.80 (dd, *J* = 15.0, 6.8 Hz, 1H), 2.39 (dd, *J* = 14.9, 8.9 Hz, 1H), 1.58 (s, 3H).

*Minor diastereoisomer:*

**^1^H NMR** (400 MHz, CDCl_3_): δ [ppm] = 8.56 (d, *J* = 4.5 Hz, 1H), 7.57 – 7.52 (m, 1H), 7.22 – 7.11 (m, 11H), 7.04 (d, *J* = 8.0 Hz, 1H), 4.70 (d, *J* = 15.1 Hz, 1H), 4.18 (d, *J* = 15.1 Hz, 1H), 3.68 – 3.62 (m, 1H), 3.26 (dd, *J* = 15.6, 6.5 Hz, 1H), 3.01 (dd, *J* = 15.4, 8.9 Hz, 1H), 1.51 (s, 3H).

**^13^C NMR** (101 MHz, CDCl_3_): δ [ppm] = 169.8, 158.9, 149.3, 136.2, 128.8, 128.7, 128.6, 128.4, 128.3, 127.7, 126.2, 122.5, 121.9, 119.9, 63.6, 44.5, 31.6, 23.8, 19.1.

**HRMS (ESI)** *m/z*: [M+Na] ^+^ calculated for C_23_H_22_N_2_ONa^+^: 365.1524, found: 365.1626.

1-Benzyl-3-(3-chloropropyl)-4-methyl-4-(pyridin-2-yl)azetidin-2-one **(b39)**

Following the general procedure B, **b39** was obtained as a white solid (24.9 mg, 0.076 mmol, 38%) after column chromatography (silica, PE/EtOAc 2:1). d.r. > 8/1. *Major diastereoisomer:*

**^1^H NMR** (400 MHz, CDCl_3_): δ [ppm] = 8.63 (d, *J* = 4.5 Hz, 1H), 7.63 (t, *J* = 7.7 Hz, 1H), 7.34 – 7.26 (m, 5H), 7.23 – 7.16 (m, 2H), 4.74 (d, *J* = 15.1 Hz, 1H), 4.14 (d, *J* = 15.1 Hz, 1H), 3.37 – 3.26 (m, 2H), 3.12 (t, *J* = 8.1 Hz, 1H), 1.82 – 1.75 (m, 1H), 1.65 (s, 3H), 1.62 – 1.55 (m, 1H), 1.38 – 1.31 (m, 1H), 1.22 – 1.15 (m, 1H).

**^13^C NMR** (101 MHz, CDCl_3_): δ [ppm] = 169.9, 159.2, 149.5, 137.0, 136.5, 128.9, 128.8, 127.8, 122.6, 121.4, 66.2, 62.2, 44.7, 44.5, 30.2, 23.8, 23.6.

**HRMS (ESI)** *m/z*: [M+Na] ^+^ calculated for C_19_H_21_ClN_2_ONa^+^: 351.1235, found: 351.1227

**1-Benzyl-3-(but-3-yn-1-yl)-4-methyl-4-(pyridin-2-yl)azetidin-2-one** **(b40)**

Following the general procedure B, **b40** was obtained as a white solid (19.5 mg, 0.064 mmol, 32%) after column chromatography (silica, PE/EtOAc 2:1). d.r. = 3/1. *Major diastereoisomer:*

**^1^H NMR** (400 MHz, CDCl_3_): δ [ppm] = 8.61 (d, *J* = 4.6 Hz, 1H), 7.61 (t, *J* = 7.8 Hz, 1H), 7.26 – 7.15 (m, 7H), 4.73 (d, *J* = 15.1 Hz, 1H), 4.11 (d, *J* = 15.1 Hz, 1H), 3.33 (t, *J* = 8.0 Hz, 1H), 2.10 – 2.03 (m, 2H), 1.86 (t, *J* = 2.6 Hz, 1H), 1.65 (s, 3H), 1.57 – 1.50 (m, 1H), 1.24 – 1.16 (m, 1H).

*Minor diastereoisomer:*

**^1^H NMR** (400 MHz, CDCl_3_): δ [ppm] = 8.56 (d, *J* = 4.6 Hz, 1H), 7.58 – 7.52 (m, 1H), 7.19 – 7.12 (m, 7H), 4.62 (d, *J* = 15.1 Hz, 1H), 4.15 (d, *J* = 15.4 Hz, 1H), 3.64 – 3.60 (m, 1H), 2.41 – 2.34 (m, 2H), 2.02 – 1.99 (m, 1H), 1.50 (s, 3H), 1.46 – 1.40 (m, 1H), 1.38 – 1.32 (m, 1H).

**^13^C NMR** (101 MHz, CDCl_3_): δ [ppm] = 169.9, 159.3, 149.6, 137.0, 136.4, 128.9, 128.7, 127.7, 122.52, 121.4, 83.3, 69.0, 66.1, 61.4, 44.5, 25.1, 18.9, 16.3.

**HRMS (ESI)** *m/z*: [M+Na] ^+^ calculated for C_20_H_20_N_2_ONa^+^: 327.1467, found: 327.1470.

2-Benzyl-3-ethyl-3-(pyridin-2-yl)-2-azaspiro[3.5]nonan-1-one **(b41)**

Following the general procedure B, **b41** was obtained as a white solid (49.5 mg, 0.148 mmol, 74%) after column chromatography (silica, PE/EtOAc 2:1).

**^1^H NMR** (400 MHz, CDCl_3_): δ [ppm] = 8.63 (d, *J* = 4.1 Hz, 1H), 7.64 (t, *J* = 7.7 Hz, 1H), 7.39 – 7.24 (m, 5H), 7.20 – 7.09 (m, 2H), 5.07 (d, *J* = 16.0 Hz, 1H), 4.23 (d, *J* = 16.0 Hz, 1H), 2.50 – 2.40 (m, 1H), 2.21 – 2.09 (m, 1H), 1.89 – 1.79 (m, 2H), 1.75 – 1.59 (m, 4H), 1.38 – 1.29 (m, 1H), 1.20 – 1.07 (m, 2H), 0.88 – 0.80 (m, 1H), 0.53 (t, *J* = 7.4 Hz, 3H).

**^13^C NMR** (101 MHz, CDCl_3_): δ [ppm] = 174.9, 160.3, 149.4, 138.5, 136.1, 128.7, 127.6, 127.3, 122.0, 121.8, 74.1, 62.1, 45.2, 30.8, 29.4, 26.3, 25.8, 24.0, 23.5, 9.9.

**HRMS (ESI)** *m/z*: [M+Na] ^+^ calculated for C_22_H_26_N_2_ONa^+^: 357.1937, found: 357.1947.

2-Benzyl-3-propyl-3-(pyridin-2-yl)-2-azaspiro[3.5]nonan-1-one **(b42)**

Following the general procedure B, **b42** was obtained as a white solid (47.4 mg, 0.136 mmol, 68%) after column chromatography (silica, PE/EtOAc 2:1).

**^1^H NMR** (400 MHz, CDCl_3_): δ [ppm] = 8.62 (d, *J* = 4.2 Hz, 1H), 7.63 (td, *J* = 7.7, 1.5 Hz, 1H), 7.39 – 7.21 (m, 5H), 7.20 – 7.10 (m, 2H), 5.10 (d, *J* = 15.9 Hz, 1H), 4.19 (d, *J* = 15.9 Hz, 1H), 2.44 – 2.31 (m, 1H), 2.24 – 2.11 (m, 1H), 1.95 – 1.84 (m, 1H), 1.80 – 1.55 (m, 5H), 1.43 – 1.31 (m, 1H), 1.20 – 1.02 (m, 2H), 0.93 – 0.77 (m, 3H), 0.46 (t, *J* = 7.2 Hz, 3H).

**^13^C NMR** (101 MHz, CDCl_3_): δ [ppm] = 174.9, 160.7, 149.4, 138.5, 136.1, 128.7, 127.7, 127.3, 121.7, 121.7, 73.6, 62.1, 45.1, 35.9, 30.8, 29.3, 25.8, 24.0, 23.5, 19.0, 14.4.

**HRMS (ESI)** *m/z*: [M+Na] ^+^ calculated for C_23_H_28_N_2_ONa^+^: 371.2094 , found: 371.2095.

2-Benzyl-3-isobutyl-3-(pyridin-2-yl)-2-azaspiro[3.5]nonan-1-one **(b43)**

Following the general procedure B, **b43** was obtained as a white solid (52.2 mg, 0.144 mmol, 72%) after column chromatography (silica, PE/EtOAc 5:1).

**^1^H NMR** (400 MHz, CDCl_3_): δ [ppm] = 8.59 (d, *J* = 4.2 Hz, 1H), 7.59 (td, *J* = 7.8, 1.6 Hz, 1H), 7.40 – 7.29 (m, 4H), 7.27 – 7.21 (m, 1H), 7.18 – 7.09 (m, 2H), 5.04 (d, *J* = 16.1 Hz, 1H), 4.46 (d, *J* = 16.1 Hz, 1H), 2.34 – 2.19 (m, 2H), 1.98 – 1.86 (m, 1H), 1.84 – 1.78 (m, 1H), 1.75 – 1.62 (m, 4H), 1.47 – 1.37 (m, 2H), 1.21 – 1.09 (m, 1H), 0.99 – 0.88 (m, 2H), 0.46 (d, *J* = 6.7 Hz, 3H), 0.28 (d, *J* = 6.6 Hz, 3H).

**^13^C NMR** (101 MHz, CDCl_3_): δ [ppm] = 175.2, 161.3, 148.9, 138.6, 135.9, 128.8, 127.6, 127.3, 121.9, 121.8, 73.8, 62.6, 45.7, 42.6, 31.6, 29.6, 25.8, 25.4, 25.2, 24.1, 23.7, 23.5.

**HRMS (ESI)** *m/z*: [M+H] ^+^ calculated for C_24_H_31_N_2_O: 363.2431 , found: 363.2424.

2-Benzyl-3-hexyl-3-(pyridin-2-yl)-2-azaspiro[3.5]nonan-1-one **(b44)**

Following the general procedure B, **b44** was obtained as a yellow solid (64.0 mg, 0.164 mmol, 82%) after column chromatography (silica, PE/EtOAc 2:1).

**^1^H NMR** (400 MHz, CDCl_3_): δ [ppm] = 8.63 (d, *J* = 4.4 Hz, 1H), 7.64 (td, *J* = 7.7, 1.5 Hz, 1H), 7.40 – 7.27 (m, 4H), 7.27 – 7.23 (m, 1H), 7.20 – 7.10 (m, 2H), 5.12 (d, *J* = 16.0 Hz, 1H), 4.18 (d, *J* = 16.0 Hz, 1H), 2.40 – 2.31 (m, 1H), 2.22 – 2.10 (m, 1H), 1.98 – 1.83 (m, 2H), 1.80 – 1.74 (m, 1H), 1.66 – 1.57 (m, 3H), 1.50 – 1.26 (m, 3H), 1.21 – 0.97 (m, 4H), 0.91 – 0.85 (m, 2H), 0.84 – 0.77 (m, 2H), 0.72 (t, *J* = 7.3 Hz, 3H), 0.68 – 0.61 (m, 1H).

**^13^C NMR** (101 MHz, CDCl_3_): δ [ppm] = 174.9, 160.7, 149.4, 138.6, 136.1, 128.7, 127.7, 127.3, 121.7, 73.6, 62.2, 45.0, 33.8, 31.5, 30.8, 29.8, 29.4, 25.8, 25.5, 24.0, 23.5, 22.6, 14.1.

**HRMS (ESI)** *m/z*: [M+Na] ^+^ calculated for C_26_H_34_N_2_ONa^+^: 413.2563, found: 413.2563.

1-Benzyl-4-hexyl-4-(pyridin-2-yl)-3-(p-tolyl)azetidin-2-one **(b45)**

Following the general procedure B, **b45** was obtained as a white solid (66.0 mg, 0.160 mmol, 80%) after column chromatography (silica, PE/EtOAc 2:1). d.r. = 3/1. *Major diastereoisomer:*

**^1^H NMR** (400 MHz, CDCl_3_): δ [ppm] = 8.40 (d, *J* = 4.4 Hz, 1H), 7.40 (d, *J* = 7.1 Hz, 2H), 7.36 – 7.22 (m, 4H), 6.97 – 6.90 (m, 1H), 6.87 – 6.70 (m, 5H), 4.99 (d, *J* = 15.3 Hz, 1H), 4.55 (s, 1H), 4.43 (d, *J* = 15.4 Hz, 1H), 2.33 – 2.15 (m, 2H), 2.14 (s, 3H), 1.31 – 1.24 (m, 1H), 1.18 – 0.93 (m, 7H), 0.80 (t, *J* = 7.2 Hz, 3H).

**^13^C NMR** (101 MHz, CDCl_3_): δ [ppm] = 168.9, 158.9, 148.8, 137.6, 136.5, 135.4, 130.3, 128.7, 127.6, 122.2, 121.7, 72.2, 65.0, 45.7, 37.1, 31.7, 29.6, 24.7, 22.6, 21.1, 14.2.

**HRMS (ESI)** *m/z*: [M+Na] ^+^ calculated for C_28_H_32_N_2_ONa^+^: 435.2407, found: 435.2410.

**1-Benzyl-2-methyl-4-oxo-3-phenyl-2-(pyridin-2-yl)azetidin-3-yl acetate** **(b46)**

Following the general procedure B, **b46** was obtained as a white solid (60.2 mg, 0.156 mmol, 78%) after column chromatography (silica, PE/EtOAc 2:1). d.r. > 10/1. *Major diastereoisomer:*

**^1^H NMR** (600 MHz, CDCl_3_): δ [ppm] = 8.39 (s, 1H), 7.37 (d, *J* = 6.3 Hz, 2H), 7.30 – 7.24 (m, 3H), 7.23 (t, *J* = 6.9 Hz, 1H), 7.14 (s, 2H), 6.99 (s, 3H), 6.90 (s, 1H), 6.77 (d, *J* = 7.7 Hz, 1H), 4.98 (d, *J* = 15.0 Hz, 1H), 4.31 (d, *J* = 15.0 Hz, 1H), 2.13 (s, 3H), 1.72 (s, 3H).

**^13^C NMR** (101 MHz, CDCl_3_): δ [ppm] = 168.4, 165.3, 158.4, 148.8, 136.6, 135.6, 134.5, 129.2, 128.8, 128.0, 127.9, 127.7, 127.5, 122.1, 122.0, 73.2, 44.7, 21.5, 20.2.

**HRMS (ESI)** *m/z*: [M+Na] ^+^ calculated for C_24_H_22_N_2_O_3_Na^+^_:_ 409.1522, found: 409.1512.

3-Decyl-1,4-dimethyl-4-(pyridin-2-yl)azetidin-2-one **(b47)**

Following the general procedure B, **b47** was obtained as a white solid (29.4 mg, 0.094 mmol, 47%) after column chromatography (silica, PE/EtOAc 3:1). d.r. = 8/1. *Major diastereoisomer:*

**^1^H NMR** (400 MHz, CDCl_3_): δ [ppm] = 8.64 (d, *J* = 4.6 Hz, 1H), 7.71 (t, *J* = 7.7 Hz, 1H), 7.33 – 7.27 (m, 1H), 7.21 (d, *J* = 6.0 Hz, 1H), 3.07 (t, *J* = 7.8 Hz, 1H), 2.81 (s, 3H), 1.84 (s, 3H), 1.26 – 1.19 (m, 10H), 1.14 – 0.96 (m, 8H), 0.86 (t, *J* = 6.7 Hz, 3H).

*Minor diastereoisomer:*

**^1^H NMR** (400 MHz, CDCl_3_): δ [ppm] = 8.60 (d, *J* = 4.5 Hz, 1H), 7.21 (d, *J* = 6.0 Hz, 3H), 3.02 – 2.97 (m, 1H), 2.79 (s, 3H), 1.69 (s, 3H), 1.14 – 1.04 (m, 21H).

**^13^C NMR** (101 MHz, CDCl_3_): δ [ppm] = 170.7, 149.5, 136.5, 122.4, 121.8, 119.1, 65.1, 63.1, 32.0, 29.7, 29.6, 29.6, 29.5, 29.4, 29.3, 27.2, 26.2, 25.6, 24.8, 22.8, 22.0, 14.2.

**HRMS (ESI)** *m/z*: [M+H] ^+^ calculated for C_20_H_33_N_2_O: 317.2587, found:317.2591.

1-Benzyl-3-(4-isobutylphenyl)-3,4-dimethyl-4-(pyridin-2-yl)azetidin-2-one **(b48)**

Following the general procedure B, **b48** was obtained as a white solid (76.4 mg, 0.192 mmol, 96%) after column chromatography (silica, PE/EtOAc 4:1). d.r. = 5/1. *Major diastereoisomer:*

**^1^H NMR** (400 MHz, CDCl_3_): δ [ppm] = 8.36 (d, *J* = 4.2 Hz, 1H), 7.39 – 7.19 (m, 7H), 7.14 – 7.09 (m, 1H), 6.90 (d, *J* = 8.0 Hz, 2H), 6.84 – 6.78 (m, 1H), 6.71 (d, *J* = 8.1 Hz, 2H), 4.81 (d, *J* = 15.1 Hz, 1H), 4.16 (d, *J* = 15.1 Hz, 1H), 2.27 – 2.19 (m, 2H), 1.72 (d, *J* = 3.8 Hz, 6H), 1.68 – 1.59 (m, 1H), 0.71 (t, *J* = 6.3 Hz, 6H).

*Minor diastereoisomer:*

^1^H NMR (400 MHz, CDCl_3_): δ [ppm] = 8.68 (d, *J* = 3.6 Hz, 1H), 7.64 (td, *J* = 7.7, 1.6 Hz, 1H), 7.43 – 7.33 (m, 6H), 7.25 – 7.17 (m, 5H), 4.90 (d, *J* = 15.3 Hz, 1H), 4.38 (d, *J* = 15.2 Hz, 1H), 2.46 (d, *J* = 6.4 Hz, 2H), 1.85 – 1.79 (m, 1H), 1.11 (d, *J* = 10.5 Hz, 6H), 0.89 (dd, *J* = 6.5, 3.2 Hz, 6H).

**^13^C NMR** (101 MHz, CDCl_3_): δ [ppm] = 172.3, 160.4, 148.5, 139.6, 137.3, 137.1, 135.2, 128.9, 128.8, 128.4, 127.7, 126.8, 121.7, 121.4, 71.1, 67.8, 44.9, 44.1, 30.2, 22.2, 22.1, 20.5, 19.8.

**HRMS (ESI)** *m/z*: [M+Na] ^+^ calculated for C_27_H_30_N_2_ONa^+^: 421.2250, found: 421.2248.

**3-(4-Benzoylphenyl)-1,3,4-trimethyl-4-(pyridin-2-yl)azetidin-2-one** **(b49)**

Following the general procedure B, **b49** was obtained as a white solid (37.8 mg, 0.102 mmol, 51%) after column chromatography (silica, PE/EtOAc 3:1). d.r. = 5/1. *Major diastereoisomer:*

**^1^H NMR** (400 MHz, CDCl_3_): δ [ppm] = 8.28 (d, *J* = 3.8 Hz, 1H), 7.53 – 7.41 (m, 3H), 7.38 – 7.30 (m, 2H), 7.26 – 7.15 (m, 4H), 7.02 – 6.94 (m, 1H), 6.85 – 6.73 (m, 2H), 2.78 (s, 3H), 1.83 (s, 3H), 1.65 (s, 3H).

*Minor diastereoisomer:*

**^1^H NMR** (400 MHz, CDCl_3_): δ [ppm] = 8.65 (d, *J* = 3.8 Hz, 1H), 7.96 (s, 1H), 7.84 – 7.69 (m, 5H), 7.60 (d, *J* = 7.2 Hz, 1H), 7.50 – 7.42 (m, 2H), 7.29 – 7.20 (m, 4H), 3.00 (s, 3H), 1.37 (s, 3H), 1.14 (s, 3H).

**^13^C NMR** (101 MHz, CDCl_3_): δ [ppm] = 196.4, 171.5, 160.0, 149.1, 140.3, 137.5, 137.2, 135.9, 132.6, 131.2, 130.1, 128.7, 128.3, 128.1, 127.7, 121.9, 120.9, 70.0, 67.7, 24.7, 20.1, 18.4.

**HRMS (ESI)** *m/z*: [M+H] ^+^ calculated for C_24_H_23_N_2_O_2:_ 371.1754 , found: 371.1753.

# 3. UV/Vis Spectra

**Fig. S3** UV/Vis Spectra of **a1**

The UV/Vis spectrum of **a1** revealed a max absorption at λ_max_ = 277 nm, **ε**_277 nm_ = 2103.9 M**^−^**^1^cm**^−^**^1^.

**Fig. S4** UV/Vis Spectra of **a1** and with various equiv. Zn(OTf)_2_

# 4. Mechanistic Studies

## 4.1 Stern-Volmer Quenching Experiments

Emission intensities were recorded using a Hitachi F-7000 fluorescence spectrometer with a 10 nm band width. Solutions of different concentration of **a1** were prepared and introduced to a 1 cm path length quartz cuvette equipped with a Teflon® septum under Ar atmosphere. In a typical experiment, to a 0.05 mM solution of photocatalyst in DCM was added the appropriate amount **a1** in screw-top quartz cuvette under Ar atmosphere and the emission of the sample was collected. [Ir(dFCF_3_ppy)_2_dtbbpy]PF**_6_** was excited at 375 nm and the emission intensity was collected at 420-700 nm The solutions mixtures were stirred for 30 s in dark, then transferred to 1.0 cm path length quartz cuvettes, sealed with Teflon® septum.^[3]^

| **PC**/ (mM) | **a1** (mM) | I_0_/I |
| --- | --- | --- |
| 0.025 | 0 | 1 |
| 0.025 | 5 | 1.08860 |
| 0.025 | 10 | 1.37307 |
| 0.025 | 15 | 1.64730 |
| 0.025 | 20 | 1.89838 |
| 0.025 | 25 | 2.19918 |

**Fig. S5** The Stern-Volmer plot for the quenching

Emission intensities were recorded using a Hitachi F-7000 fluorescence spectrometer with a 10 nm band width. Solutions of different concentration of **a1’** (**a1** and 0.2 equiv. Zn(OTf)_2_ stirred in DCM for 2 h) were prepared and introduced to a 1 cm path length quartz cuvette equipped with a Teflon® septum under Ar atmosphere. In a typical experiment, to a 0.05 mM solution of photocatalyst in CH_3_CN was added the appropriate amount **a1’** in screw-top quartz cuvette under Ar atmosphere and the emission of the sample was collected.^[3]^ [Ir(dFCF_3_ppy)_2_dtbbpy]PF was excited at 375 nm and the emission intensity was collected at 420-700 nm The solutions mixtures were stirred for 30 s in dark, then transferred to 1.0 cm path length quartz cuvettes, sealed with Teflon® septum.

| **PC**/ (mM) | **a1'** (mM) | I_0_/I |
| --- | --- | --- |
| 0.025 | 0 | 1 |
| 0.025 | 5 | 1.90852 |
| 0.025 | 10 | 3.04636 |
| 0.025 | 15 | 3.80671 |
| 0.025 | 20 | 4.85566 |
| 0.025 | 25 | 6.38601 |

**Fig. S6** The Stern-Volmer plot for the quenching

## 4.2 Deuteration Experiments and KIE

***Deuteration Experiments***

The **a10-***d_1_* (0.2 mmol, 1.0 equiv.), [Ir(dFppy)_2_dtbbpy]PF_6_ (4.0 mg, 0.004 mmol, 2 mol%) were added to an oven-dried 20 mL glass vial equipped with a stir bar and capped with a septum. After evacuated/backfilled with Argon (3 times), added the DCM (10 mL) and Zn(OTf)_2_ (14.5 mg, 0.04 mmol, 0.2 equiv.). After irradiation λ_max_ = 427 nm, Kessil lamp at rt for 5 hours, added saturated aqueous NaHCO_3_ and extracted with EtOAc. The organic extracts were combined, washed with NaCl (aq.), dried over Na_2_SO_4_, filtered and concentrated in vacuo. The product was purified by column chromatography (silica, PE/EtOAc = 4/1) to obatin **b10-***d_1_* ( mg, mmol, %) as white solid.

***N*-benzyl-2-phenyl-*N*-(1-(pyridin-2-yl)vinyl)propanamide-2-*d* (a10-***d_1_***)**

**^1^H NMR** (600 MHz, CDCl_3_): δ [ppm] = 8.63 (d, *J* = 4.0 Hz, 1H), 7.56 (s, 1H), 7.26 – 7.14 (m, 11H), 7.06 (s, 1H), 6.30 (s, 1H), 4.86 – 4.39 (m, 3H), 1.43 (s, 3H).

**^2^D NMR** (600 MHz, CDCl_3_): δ [ppm] = 2.23 (s, 1D)

**HRMS (ESI)** *m/z*: [M+H] ^+^ calculated for C_23_H_22_DN_2_O: 344.1868, found: 344.1867

**1-Benzyl-3-methyl-4-(methyl-*d*)-3-phenyl-4-(pyridin-2-yl)azetidin-2-one (b10-***d_1_***)**

**^1^H NMR** (400 MHz, CDCl_3_): δ [ppm] = δ 8.39 (d, *J* = 4.0 Hz, 1H), 7.35 – 7.26 (m, 6H), 7.15 (t, *J* = 7.7 Hz, 1H), 7.01 – 6.84 (m, 5H), 6.72 (d, *J* = 8.0 Hz, 1H), 4.81 (d, *J* = 15.1 Hz, 1H), 4.15 (d, *J* = 15.1 Hz, 1H), 1.72 (s, 5H).

**^2^D NMR** (600 MHz, CDCl_3_): δ [ppm] = 0.74 (s, 1D).

**HRMS (ESI)** *m/z*: [M+H] ^+^ calculated for C_23_H_22_DN_2_ONa^+^:366.1687, found: 366.1689

***Kinetic isotope effect***

***KIE determined from intermolecular competition***

The **a10** (0.1 mmol, 1.0 equiv.), **a10-***d_1_* (0.1 mmol, 1.0 equiv.), [Ir(dFppy)_2_dtbbpy]PF_6_ (4.0 mg, 0.004mmol, 2 mol%) were added to an oven-dried 20 mL glass vial equipped with a stir bar and capped with a septum. After evacuated/backfilled with Argon (3 times), added the DCM (10 mL) and Zn(OTf)_2_ (0.04 mmol, 14.5 mg, 0.2 equiv.). After irradiation λ_max_ = 427 nm, Kessil lamp at rt for 1 hours, added saturated aqueous NaHCO_3_ and extracted with EtOAc. The organic extracts were combined, washed with NaCl (aq.), dried over Na_2_SO_4_, filtered and concentrated in vacuo. The yield was determined by ^1^H-NMR using CH_2_Br_2_ as the internal standard. ***KIE = 2.3***.

***KIE determined from parallel reactions***

(1) The **a10** (0.2 mmol, 1.0 equiv.), [Ir(dFppy)_2_dtbbpy]PF_6_ (4.0 mg, 0.004mmol, 2 mol%) were added to an oven-dried 20 mL glass vial equipped with a stir bar and capped with a septum. After evacuated/backfilled with Argon (3 times), added the DCM (10 mL) and Zn(OTf)_2_ (0.04 mmol, 14.5 mg, 0.2 equiv.). After irradiation λ_max_ = 427 nm, Kessil lamp at rt for 1 hours, added saturated aqueous NaHCO_3_ and extracted with EtOAc. The organic extracts were combined, washed with NaCl (aq.), dried over Na_2_SO_4_, filtered and concentrated in vacuo. The yield was determined by ^1^H-NMR using CH_2_Br_2_ as the internal standard.
(2) **a10-***d_1_* (0.2 mmol, 1.0 equiv.), [Ir(dFppy)_2_dtbbpy]PF_6_ (4.0 mg, 0.004mmol, 2 mol%) were added to an oven-dried 20 mL glass vial equipped with a stir bar and capped with a septum. After evacuated/backfilled with Argon (3 times), added the DCM (10 mL) and Zn(OTf)_2_ (0.04 mmol, 14.5 mg, 0.2 equiv.). After irradiation λ_max_ = 427 nm, Kessil lamp at rt for 1 hours, added saturated aqueous NaHCO_3_ and extracted with EtOAc. The organic extracts were combined, washed with NaCl (aq.), dried over Na_2_SO_4_, filtered and concentrated in vacuo. The yield was determined by ^1^H-NMR using CH_2_Br_2_ as the internal standard. ***KIE = 2.9***

## 4.3 Controlled Experiments

**Photochemical reaction of a50**

The respective **a50** (67.1 mg, 0.2 mmol, 1.0 equiv.), Zn(OTf)_2_ (14.6 mg, 0.04 mmol, 20 mol%) and Ir(dFppy)_2_(dtbbpy)PF_6_ (4.0 mg, 0.004 mmol, 2 mol%) were added to an oven-dried 20 mL glass vial equipped with a stir bar and capped with a septum. After evacuated/backfilled with Argon (3 times), added the DCM (10 mL). After irradiation (λ_max_ = 427 nm, Kessil lamp, 3 vials with one lamp) at rt for 36 hours, added saturated aqueous NaHCO_3_ and extracted with EtOAc. The organic extracts were combined, washed with NaCl (aq.), dried over Na_2_SO_4_, filtered and concentrated in vacuo. The product was purified by column chromatography (silica, PE/EtOAc) to yield the product. The product cannot be observed by ^1^H-NMR.

**Photochemical reaction of a51**

The respective **a51** (38.4 mg, 0.2 mmol, 1.0 equiv.), Zn(OTf)_2_ (14.6 mg, 0.04 mmol, 20 mol%) and Ir(dFppy)_2_(dtbbpy)PF_6_ (4.0 mg, 0.004 mmol, 2 mol%) were added to an oven-dried 20 mL glass vial equipped with a stir bar and capped with a septum. After evacuated/backfilled with Argon (3 times), added the DCM (10 mL). After irradiation (λ_max_ = 427 nm, Kessil lamp, 3 vials with one lamp) at rt for 36 hours, added saturated aqueous NaHCO_3_ and extracted with EtOAc. The organic extracts were combined, washed with NaCl (aq.), dried over Na_2_SO_4_, filtered and concentrated in vacuo. The product cannot be observed by ^1^H-NMR.

**4.4** **Triplet Energy Quenching Experiment**

*N*-benzyl-*N*-(1-(pyridin-2-yl)vinyl)cyclohexanecarboxamide (64.0 mg, 0.20 mmol) and Zn(OTf)_2_ (14.6 mg, 0.04 mmol, 20 mol%) and Ir(dFppy)_2_(dtbbpy)PF_6_ (4.0 mg, 0.004 mmol, 2 mol%) were added to an oven-dried 20 mL glass vial equipped with a stir bar and capped with a septum. After evacuated/backfilled with Argon (3 times), added the DCM (10 mL) and 2,5-dimethylhexa-2,4-diene^[4]^ (44.0 mg, 0.4 mmol. 2.0 equiv.). After irradiation (λ_max_ = 427 nm, Kessil lamp, 3 vials with one lamp) at rt for 36 hours, added saturated aqueous NaHCO_3_ and extracted with EtOAc. The organic extracts were combined, washed with NaCl (aq.), dried over Na_2_SO_4_, filtered and concentrated in vacuo. The yield (31%) was determined by ^1^H-NMR using CH_2_Br_2_ as the internal standard.

## 4.5 DFT Studies

**Computational details**

All the geometry optimizations and frequency calculations reported in this paper were obtained with the ORCA 6.1.0 program. Electron correlation was partially taken into account using the B3LYP functional in conjunction with the D3(BJ) dispersion correction suggested by Grimme et al. , the resolution-of-identity approach, RI(JCOSX), and the double-ζ quality plus polarization functions def2-SVP basis set for all atoms. Solvent effects (solvent = dichloromethane) were taken into account by using the conductor-like polarizable continuum model (CPCM). All species were characterized by frequency calculations: reactants and adducts exhibited positive definite Hessian matrices, while transition states showed a single negative eigenvalue in their diagonalized force constant matrices. Single-point energy refinements were carried out at the same DFT level using the much larger triple-ζ quality def2-TZVPP basis sets. This level is denoted CPCM(dichloromethane)-RI-B3LYP-D3BJ/def2-TZVPP//CPCM(dichloromethane)-RI-B3LYP-D3BJ/def2-SVP.

TD-DFT calculations were performed at the PCM-B3LYP-D3/def2-SVP level using the CPCM(dichloromethane)-RI-B3LYP-D3BJ/def2-SVP optimized geometry with the Gaussian 16 suite of programs. The B3LYP Hamiltonian was chosen because it was proven to provide reasonable UV/Vis spectra for a variety of chromophores.^[5-13]^

Cartesian coordinates (in Å) and free energies (in a.u.) of all the stationary points discussed in the text. All calculations have been performed at the CPCM(dichloromethane)-RI-B3LYP-D3BJ/def2-TZVPP//PCM(dichloromethane)-RI-B3LYP-D3BJ/def2-SVP level.

```
a19’-S0, G = -882.272103

C        1.865988000      3.331531000     -1.342734000
N        2.921851000      3.683197000     -0.600630000
C        3.083166000      3.050498000      0.569122000
C        2.218289000      2.063377000      1.041896000
C        1.099965000      1.701795000      0.271377000
C        0.940272000      2.358537000     -0.960052000
H        1.741476000      3.849583000     -2.300866000
H        3.956485000      3.336048000      1.167062000
C        0.130877000      0.668763000      0.721743000
C        0.003872000      0.278774000      2.000681000
H        0.555197000      0.768868000      2.804664000
H       -0.683396000     -0.525683000      2.268494000
N       -0.641262000      0.050747000     -0.308757000
C       -2.017734000      0.076248000     -0.403069000
O       -2.596545000     -0.616887000     -1.234734000
C       -2.797301000      1.043058000      0.495710000
H       -2.404190000      0.967100000      1.518407000
C       -2.592377000      2.491483000      0.019542000
H       -3.218230000      3.173755000      0.615753000
H       -1.548294000      2.818060000      0.122789000
H       -2.882553000      2.598387000     -1.038630000
C       -4.278381000      0.663425000      0.516874000
H       -4.423967000     -0.368191000      0.872194000
H       -4.829084000      1.341411000      1.187320000
H       -4.717767000      0.734997000     -0.489524000
C        0.108754000     -0.842318000     -1.213225000
H       -0.522390000     -1.004941000     -2.094622000
H        1.024750000     -0.322640000     -1.526953000
C        0.459387000     -2.153681000     -0.547628000
C       -0.527201000     -3.133022000     -0.342696000
C        1.759375000     -2.392024000     -0.080837000
C       -0.215436000     -4.326227000      0.313526000
C        2.074566000     -3.587660000      0.574842000
C        1.087170000     -4.557195000      0.773816000
H       -1.542640000     -2.946020000     -0.699411000
H        2.531198000     -1.631733000     -0.231756000
H       -0.990770000     -5.082130000      0.464908000
H        3.093173000     -3.760173000      0.932499000
H        1.330361000     -5.491822000      1.286079000
H        2.430843000      1.573559000      1.993529000
H        0.100075000      2.118964000     -1.613171000

a19’-Zn-S0, G = -4584.700742

C        1.938643000      2.009481000     -0.762316000
N        2.858316000      2.070311000      0.220861000
C        2.521591000      1.673446000      1.465091000
C        1.265156000      1.166271000      1.757133000
C        0.315775000      1.034310000      0.732372000
C        0.669248000      1.497873000     -0.546223000
H        2.253588000      2.346573000     -1.751164000
H        3.301517000      1.750225000      2.226134000
C       -0.982385000      0.355162000      0.970631000
C       -1.617901000      0.419041000      2.151819000
H       -1.251000000      1.061583000      2.954065000
H       -2.534879000     -0.146383000      2.326212000
N       -1.489191000     -0.406403000     -0.120514000
C       -2.718112000     -0.211449000     -0.722322000
O       -3.086633000     -0.990295000     -1.594787000
C       -3.560296000      1.015057000     -0.348338000
H       -3.597881000      1.103811000      0.745414000
C       -2.911874000      2.295031000     -0.902785000
H       -3.572447000      3.157235000     -0.721949000
H       -1.945738000      2.512314000     -0.425775000
H       -2.749398000      2.211830000     -1.989884000
C       -4.989293000      0.843878000     -0.865915000
H       -5.456118000     -0.067704000     -0.462943000
H       -5.600900000      1.708700000     -0.565584000
H       -5.004654000      0.770107000     -1.963749000
C       -0.716810000     -1.578876000     -0.626279000
H       -1.360582000     -2.463444000     -0.501138000
H       -0.564446000     -1.450174000     -1.707449000
C        0.604085000     -1.789773000      0.069910000
C        0.654365000     -2.265081000      1.389322000
C        1.806264000     -1.472764000     -0.579349000
C        1.876516000     -2.380271000      2.056720000
C        3.031184000     -1.580897000      0.084085000
C        3.066410000     -2.026811000      1.409738000
H       -0.274235000     -2.526633000      1.903008000
H        1.778248000     -1.108898000     -1.607705000
H        1.900930000     -2.741977000      3.087903000
H        3.954459000     -1.298656000     -0.426516000
H        4.020841000     -2.102411000      1.937043000
H        1.059503000      0.803880000      2.764178000
H       -0.024112000      1.415216000     -1.382884000
Zn       4.767237000      2.460169000     -0.222481000
O        5.703976000      0.625205000     -1.022506000
O        5.963721000      3.556669000      0.836136000
S        5.705076000      1.186249000     -2.421457000
O        6.949346000      1.141194000     -3.174597000
O        5.009622000      2.532157000     -2.315897000
S        5.951221000      3.678938000      2.365003000
O        5.363754000      2.505035000      3.027684000
O        7.214458000      4.211713000      2.867813000
C        4.507600000      0.114056000     -3.385341000
F        4.579051000      0.421664000     -4.672284000
F        4.827203000     -1.162767000     -3.213058000
F        3.261456000      0.312211000     -2.955862000
C        4.699427000      5.047206000      2.604036000
F        5.098256000      6.152047000      1.981259000
F        3.514087000      4.678453000      2.109655000
F        4.561727000      5.303071000      3.902169000

a19’-Zn’-S0, G = -4584.695912

C        3.811932000      4.709900000     -1.154248000
N        4.770137000      5.393481000     -0.522061000
C        5.089253000      4.995707000      0.717450000
C        4.479790000      3.923867000      1.367782000
C        3.464397000      3.208796000      0.708494000
C        3.136932000      3.622255000     -0.592964000
H        3.556979000      5.037270000     -2.168906000
H        5.881336000      5.557043000      1.226162000
C        2.761847000      2.071046000      1.347374000
C        2.826924000      1.742813000      2.645897000
H        2.288184000      0.870744000      3.020147000
H        3.396737000      2.339622000      3.359506000
N        1.995826000      1.235697000      0.460629000
C        0.658607000      1.256401000      0.380893000
O        0.099151000      0.369573000     -0.311487000
C       -0.142567000      2.334145000      1.081779000
H        0.554884000      2.960509000      1.650908000
C       -0.844197000      3.231369000      0.048479000
H       -1.570089000      2.671855000     -0.558547000
H       -1.385804000      4.029251000      0.578650000
H       -0.119763000      3.702956000     -0.631702000
C       -1.133396000      1.690467000      2.061405000
H       -0.609690000      1.090230000      2.821096000
H       -1.704288000      2.478694000      2.575019000
H       -1.845606000      1.031233000      1.544517000
C        2.763724000      0.216998000     -0.300669000
H        2.194790000     -0.006086000     -1.210397000
H        3.713734000      0.688170000     -0.584860000
C        3.010736000     -1.026748000      0.520106000
C        2.038388000     -2.036525000      0.603158000
C        4.200486000     -1.163785000      1.251031000
C        2.250309000     -3.157031000      1.410329000
C        4.415960000     -2.287805000      2.054772000
C        3.438823000     -3.285034000      2.138341000
H        1.109372000     -1.935266000      0.040821000
H        4.963411000     -0.383173000      1.187683000
H        1.480690000     -3.929437000      1.472979000
H        5.348355000     -2.384127000      2.617076000
H        3.603877000     -4.163131000      2.768118000
H        4.807374000      3.650489000      2.371753000
H        2.366203000      3.114719000     -1.172417000
Zn      -1.528023000     -0.223314000     -1.156552000
O       -2.312861000      1.251161000     -2.407825000
O       -2.473348000     -1.785784000     -0.557478000
S       -1.467261000      0.882530000     -3.616667000
O       -2.135380000      0.882823000     -4.908469000
O       -0.690309000     -0.338771000     -3.206958000
S       -3.147760000     -1.990890000      0.805555000
O       -3.608308000     -0.736031000      1.410471000
O       -4.051382000     -3.138108000      0.774842000
C       -0.185520000      2.251415000     -3.702839000
F        0.435280000      2.203078000     -4.872362000
F       -0.775568000      3.431201000     -3.559991000
F        0.710626000      2.092293000     -2.725961000
C       -1.695360000     -2.544532000      1.848920000
F       -1.081903000     -3.575210000      1.266384000
F       -0.817469000     -1.550393000      1.995114000
F       -2.125458000     -2.918173000      3.050121000

a19’-T1, G = -882.194360

C        2.093991000      3.379403000     -1.092367000
N        2.946614000      3.805423000     -0.145765000
C        2.894645000      3.188808000      1.046361000
C        2.021624000      2.148096000      1.344561000
C        1.120947000      1.688126000      0.348122000
C        1.178799000      2.348775000     -0.908002000
H        2.140400000      3.893886000     -2.059456000
H        3.592911000      3.545355000      1.812765000
C        0.195770000      0.630083000      0.608398000
C        0.049059000     -0.024544000      1.911640000
H       -0.756700000      0.255486000      2.600002000
H        0.647104000     -0.911037000      2.153414000
N       -0.558973000      0.096593000     -0.482041000
C       -1.918786000      0.262008000     -0.614001000
O       -2.569442000     -0.369456000     -1.440962000
C       -2.581944000      1.312769000      0.272756000
H       -1.829187000      1.748460000      0.946713000
C       -3.127010000      2.437270000     -0.619666000
H       -3.618085000      3.206987000     -0.004032000
H       -2.317622000      2.918304000     -1.191786000
H       -3.864179000      2.038826000     -1.334025000
C       -3.685463000      0.658503000      1.111904000
H       -3.276155000     -0.120379000      1.774992000
H       -4.190414000      1.412286000      1.735887000
H       -4.436634000      0.190320000      0.456862000
C        0.138272000     -0.868960000     -1.352209000
H       -0.512916000     -1.023399000     -2.222060000
H        1.079273000     -0.411400000     -1.689331000
C        0.415946000     -2.176574000     -0.646308000
C       -0.640446000     -3.048279000     -0.333080000
C        1.716396000     -2.512689000     -0.247981000
C       -0.397467000     -4.231072000      0.368382000
C        1.962392000     -3.698589000      0.453919000
C        0.905425000     -4.559054000      0.765253000
H       -1.655989000     -2.786989000     -0.640977000
H        2.542487000     -1.835735000     -0.484058000
H       -1.226870000     -4.902512000      0.606112000
H        2.981823000     -3.948528000      0.759662000
H        1.094780000     -5.485024000      1.314503000
H        2.030022000      1.686947000      2.334416000
H        0.503175000      2.061323000     -1.714984000

a19’-Zn-T1, G = -4584.624988

C        2.262421000      1.835784000     -0.982629000
N        3.113839000      2.032911000      0.050470000
C        2.689765000      1.761618000      1.308154000
C        1.426750000      1.268806000      1.568713000
C        0.528712000      1.006890000      0.498862000
C        0.987311000      1.338141000     -0.805538000
H        2.639190000      2.083691000     -1.977058000
H        3.403237000      1.962229000      2.110483000
C       -0.779320000      0.474488000      0.741661000
C       -1.344076000      0.524100000      2.098079000
H       -1.726108000      1.455306000      2.526442000
H       -1.206513000     -0.335383000      2.765603000
N       -1.443836000     -0.210923000     -0.287637000
C       -2.806381000     -0.078265000     -0.577397000
O       -3.333334000     -0.821675000     -1.391059000
C       -3.573396000      1.060883000      0.086888000
H       -3.392271000      0.991012000      1.171969000
C       -3.049380000      2.422702000     -0.404318000
H       -3.615644000      3.235305000      0.075953000
H       -1.984324000      2.572171000     -0.173424000
H       -3.179784000      2.513728000     -1.494951000
C       -5.073976000      0.912567000     -0.160956000
H       -5.447707000     -0.056564000      0.201926000
H       -5.619002000      1.713696000      0.361313000
H       -5.302720000      0.980047000     -1.235626000
C       -0.709411000     -1.250102000     -1.061357000
H       -1.476787000     -1.979268000     -1.352108000
H       -0.306228000     -0.827452000     -1.993149000
C        0.383731000     -1.914115000     -0.262061000
C        0.091257000     -2.548527000      0.955840000
C        1.707929000     -1.898930000     -0.716733000
C        1.107571000     -3.138237000      1.710775000
C        2.727957000     -2.494225000      0.033199000
C        2.430878000     -3.109797000      1.252461000
H       -0.940264000     -2.571502000      1.318211000
H        1.947644000     -1.400664000     -1.658301000
H        0.867157000     -3.624807000      2.659664000
H        3.756474000     -2.463000000     -0.333828000
H        3.226756000     -3.567875000      1.845291000
H        1.139594000      1.058834000      2.599737000
H        0.338076000      1.222587000     -1.672194000
Zn       4.961839000      2.691904000     -0.309031000
O        6.456573000      1.264670000     -0.861142000
O        5.799338000      4.207203000      0.569268000
S        6.174440000      1.385130000     -2.341208000
O        7.312035000      1.446458000     -3.245416000
O        5.115996000      2.458003000     -2.466695000
S        5.738126000      4.414070000      2.086396000
O        5.370139000      3.185259000      2.806985000
O        6.865369000      5.205004000      2.572372000
C        5.267789000     -0.199968000     -2.750014000
F        4.782950000     -0.138234000     -3.983276000
F        6.097129000     -1.230081000     -2.648490000
F        4.259343000     -0.366756000     -1.891333000
C        4.244294000      5.527891000      2.233309000
F        4.468038000      6.683744000      1.615093000
F        3.178581000      4.942585000      1.680637000
F        3.987207000      5.762636000      3.517520000

a19’-Zn’-T1, G = -4584.613910

C        3.952014000      4.670133000     -1.163000000
N        4.757309000      5.443456000     -0.417584000
C        4.909421000      5.107233000      0.873565000
C        4.287169000      4.014875000      1.468209000
C        3.434924000      3.191018000      0.686952000
C        3.277309000      3.554723000     -0.677316000
H        3.832900000      4.956491000     -2.214389000
H        5.568053000      5.746886000      1.472391000
C        2.767607000      2.067766000      1.263500000
C        2.862856000      1.674431000      2.670212000
H        3.619503000      0.951588000      2.995525000
H        2.106025000      1.992611000      3.397020000
N        2.012195000      1.206309000      0.394597000
C        0.672478000      1.224908000      0.337306000
O        0.094368000      0.336479000     -0.335481000
C       -0.095209000      2.318780000      1.049369000
H        0.625614000      2.905917000      1.635608000
C       -0.733384000      3.264486000      0.017934000
H       -1.456741000      2.738563000     -0.621209000
H       -1.263672000      4.069626000      0.548400000
H        0.029052000      3.722338000     -0.629601000
C       -1.125033000      1.711738000      2.010160000
H       -0.638535000      1.069943000      2.760224000
H       -1.653347000      2.521383000      2.535692000
H       -1.873107000      1.102287000      1.482800000
C        2.769397000      0.163317000     -0.347637000
H        2.180735000     -0.080052000     -1.240384000
H        3.713976000      0.624848000     -0.662190000
C        3.030044000     -1.063642000      0.494854000
C        2.031685000     -2.035116000      0.673681000
C        4.264565000     -1.227725000      1.140407000
C        2.260673000     -3.140691000      1.496405000
C        4.496941000     -2.337180000      1.959656000
C        3.492916000     -3.293821000      2.142335000
H        1.070930000     -1.916204000      0.172075000
H        5.049147000     -0.478442000      1.000492000
H        1.470691000     -3.882542000      1.633723000
H        5.463638000     -2.453986000      2.456251000
H        3.671303000     -4.160103000      2.784668000
H        4.452178000      3.792933000      2.524514000
H        2.629393000      2.981426000     -1.340931000
Zn      -1.576637000     -0.205454000     -1.134612000
O       -2.346130000      1.306616000     -2.346716000
O       -2.536950000     -1.750923000     -0.518408000
S       -1.556085000      0.915591000     -3.586475000
O       -2.269264000      0.940953000     -4.853681000
O       -0.806281000     -0.332222000     -3.206460000
S       -3.192531000     -1.948963000      0.854712000
O       -3.637633000     -0.690674000      1.463639000
O       -4.102195000     -3.091747000      0.839383000
C       -0.232982000      2.241467000     -3.716907000
F        0.349469000      2.167541000     -4.904652000
F       -0.781174000      3.440026000     -3.562806000
F        0.686293000      2.057962000     -2.766805000
C       -1.728367000     -2.508281000      1.879745000
F       -1.117769000     -3.534250000      1.285397000
F       -0.849702000     -1.514142000      2.023688000
F       -2.146300000     -2.890043000      3.082555000

TS1-T1, G = -882.181195 (i = -1528 cm-1)
C        2.783956000      2.027223000     -0.921824000
N        3.625566000      2.039148000      0.124906000
C        3.129923000      1.614990000      1.300013000
C        1.825958000      1.169523000      1.481220000
C        0.939220000      1.119046000      0.372398000
C        1.466420000      1.588083000     -0.861035000
H        3.182871000      2.396569000     -1.874458000
H        3.817353000      1.629048000      2.154408000
C       -0.407867000      0.656055000      0.525095000
C       -1.105282000      0.671128000      1.835381000
H       -0.886156000      1.512126000      2.501301000
H       -1.202651000     -0.300524000      2.345668000
N       -1.106796000      0.118194000     -0.568679000
C       -2.498655000      0.194696000     -0.661956000
O       -3.134605000     -0.491456000     -1.454940000
C       -3.154294000      1.153588000      0.292544000
H       -2.406688000      0.936456000      1.318200000
C       -2.909383000      2.621034000     -0.033720000
H       -3.252348000      3.261674000      0.793893000
H       -1.845567000      2.833012000     -0.218522000
H       -3.474644000      2.905918000     -0.938596000
C       -4.587886000      0.801939000      0.620808000
H       -4.686194000     -0.246278000      0.941402000
H       -4.971192000      1.455067000      1.419856000
H       -5.228462000      0.939708000     -0.267976000
C       -0.418389000     -0.804414000     -1.497969000
H       -1.225808000     -1.346628000     -2.007881000
H        0.135633000     -0.248133000     -2.265575000
C        0.497392000     -1.762161000     -0.773174000
C        0.003745000     -2.553915000      0.276020000
C        1.848601000     -1.868538000     -1.124489000
C        0.847104000     -3.429701000      0.963029000
C        2.695253000     -2.749454000     -0.442234000
C        2.197780000     -3.529493000      0.605623000
H       -1.049506000     -2.475593000      0.560168000
H        2.245035000     -1.244753000     -1.929728000
H        0.450090000     -4.038441000      1.779699000
H        3.748906000     -2.817908000     -0.725267000
H        2.859243000     -4.212862000      1.144497000
H        1.499592000      0.832781000      2.466922000
H        0.840304000      1.643066000     -1.751499000

INT1-T1, G = -882.215669
C        2.245846000      2.915467000     -1.369903000
N        3.186497000      3.197604000     -0.453064000
C        3.023857000      2.655388000      0.764847000
C        1.958619000      1.833704000      1.118522000
C        0.964030000      1.520136000      0.153686000
C        1.143587000      2.103143000     -1.130500000
H        2.375724000      3.369480000     -2.359689000
H        3.794158000      2.887557000      1.510116000
C       -0.160694000      0.690432000      0.457476000
C       -0.499063000      0.271309000      1.853246000
H       -0.252423000      1.045043000      2.593767000
H        0.062411000     -0.645612000      2.119787000
N       -0.880035000      0.104652000     -0.607703000
C       -2.276554000      0.016612000     -0.607286000
O       -2.851620000     -0.916757000     -1.182405000
C       -3.041399000      1.091281000      0.028696000
H       -1.566761000      0.023931000      1.946705000
C       -2.493576000      2.461101000      0.265891000
H       -2.079002000      2.565033000      1.286938000
H       -1.691225000      2.724400000     -0.437381000
H       -3.299002000      3.208828000      0.179453000
C       -4.474615000      0.833379000      0.349826000
H       -4.727037000     -0.232458000      0.281420000
H       -4.717167000      1.208374000      1.360260000
H       -5.135102000      1.386996000     -0.346361000
C       -0.136441000     -0.855783000     -1.454630000
H       -0.837353000     -1.206825000     -2.220342000
H        0.688581000     -0.326195000     -1.949605000
C        0.406588000     -2.011768000     -0.644853000
C       -0.448954000     -3.027928000     -0.188312000
C        1.758885000     -2.045750000     -0.277119000
C        0.041852000     -4.058220000      0.617519000
C        2.252452000     -3.075888000      0.531524000
C        1.394265000     -4.084232000      0.981118000
H       -1.505443000     -2.993436000     -0.464269000
H        2.429035000     -1.253620000     -0.622615000
H       -0.632889000     -4.845331000      0.964775000
H        3.309185000     -3.089431000      0.811542000
H        1.777234000     -4.889787000      1.613192000
H        1.910931000      1.423940000      2.128217000
H        0.406833000      1.938142000     -1.918246000

INT1-S0, G = -882.229018

C       -2.574060000     -2.729309000      1.184601000
N       -3.505767000     -3.250216000      0.379059000
C       -3.841438000     -2.558372000     -0.715690000
C       -3.273754000     -1.327185000     -1.054004000
C       -2.283531000     -0.797860000     -0.216812000
C       -1.931450000     -1.513811000      0.934720000
H       -2.315884000     -3.304351000      2.081082000
H       -1.177699000     -1.132280000      1.625294000
C       -1.661428000      0.523979000     -0.508693000
C       -2.539973000      1.715793000     -0.333481000
H       -2.111164000      2.615089000     -0.791609000
H       -3.535312000      1.489869000     -0.746327000
N       -0.421012000      0.611843000     -0.876060000
C        0.289096000      1.932522000     -1.129016000
O        0.678724000      2.054884000     -2.317922000
C        0.498101000      2.701815000     -0.008201000
C        0.073024000      2.378667000      1.396611000
H        0.913412000      2.552284000      2.094216000
H       -0.754145000      3.026341000      1.753279000
H       -0.244447000      1.335574000      1.536798000
C        1.282996000      3.974758000     -0.178816000
H        1.556512000      4.132201000     -1.231527000
H        0.711213000      4.859378000      0.164075000
H        2.213747000      3.961325000      0.422446000
H       -2.680073000      1.903892000      0.744615000
C        0.402301000     -0.559858000     -1.272460000
H        0.715186000     -0.328270000     -2.299162000
H       -0.226809000     -1.455000000     -1.283314000
C        1.604971000     -0.737204000     -0.376449000
C        2.707553000      0.125197000     -0.497609000
C        1.631487000     -1.750965000      0.590726000
C        3.810145000     -0.019581000      0.347690000
C        2.737177000     -1.897869000      1.435642000
C        3.825800000     -1.028117000      1.318952000
H        2.689493000      0.902833000     -1.263959000
H        0.783797000     -2.434731000      0.681141000
H        4.663943000      0.655469000      0.246192000
H        2.746966000     -2.693721000      2.184872000
H        4.689842000     -1.139443000      1.979330000
H       -4.606092000     -2.999671000     -1.364918000
H       -3.587656000     -0.802627000     -1.958886000

TS2-S0, G = -882.204638 (i = -108 cm-1)

C        2.149938000      2.594741000     -1.356785000
N        3.099991000      2.838808000     -0.442784000
C        2.896201000      2.362514000      0.791890000
C        1.769102000      1.631359000      1.166375000
C        0.775302000      1.347769000      0.203807000
C        0.989394000      1.873040000     -1.090708000
H        2.315977000      3.001958000     -2.360767000
H        3.674352000      2.572264000      1.534893000
C       -0.425234000      0.589530000      0.533187000
C       -0.704311000      0.272090000      1.978219000
H       -0.833708000      1.177466000      2.591280000
H        0.157815000     -0.281565000      2.397632000
N       -1.095465000     -0.044849000     -0.476498000
C       -2.488828000     -0.111336000     -0.259145000
O       -3.135994000     -1.166455000     -0.396239000
C       -3.009916000      1.139871000      0.212821000
H       -1.596319000     -0.355314000      2.084122000
C       -2.556350000      2.503035000     -0.220016000
H       -3.355398000      2.981596000     -0.820814000
H       -2.394899000      3.167910000      0.648156000
H       -1.641226000      2.494063000     -0.821667000
C       -4.338507000      1.094562000      0.893318000
H       -4.548427000      0.102125000      1.316101000
H       -4.390705000      1.859531000      1.687956000
H       -5.156574000      1.336332000      0.184199000
C       -0.448968000     -0.959979000     -1.441902000
H       -1.280788000     -1.459319000     -1.955884000
H        0.127291000     -0.396865000     -2.186486000
C        0.425077000     -1.968020000     -0.735165000
C       -0.158316000     -2.898484000      0.141450000
C        1.816498000     -1.955115000     -0.892374000
C        0.639990000     -3.803821000      0.843063000
C        2.617687000     -2.865112000     -0.192210000
C        2.031396000     -3.789756000      0.676825000
H       -1.244109000     -2.890876000      0.269738000
H        2.275852000     -1.223163000     -1.562451000
H        0.177536000     -4.524472000      1.522776000
H        3.702828000     -2.847000000     -0.323368000
H        2.656226000     -4.498340000      1.226834000
H        1.682781000      1.276047000      2.192808000
H        0.247017000      1.740141000     -1.877948000

b19’-S0, G = -882.277110

C        1.626339000      3.440314000     -1.462461000
N        1.999047000      4.283842000     -0.493457000
C        1.595005000      3.996895000      0.750047000
C        0.827142000      2.877082000      1.076733000
C        0.435928000      1.990969000      0.061941000
C        0.851928000      2.298620000     -1.240153000
H        1.957272000      3.680637000     -2.479874000
H        1.897486000      4.694878000      1.539752000
C       -0.430722000      0.782724000      0.364793000
C        0.042890000      0.051555000      1.622939000
H       -0.106241000      0.669544000      2.519328000
H        1.113955000     -0.187021000      1.543698000
N       -0.618164000     -0.114693000     -0.794931000
C       -1.967743000      0.033782000     -0.882804000
O       -2.796314000     -0.497811000     -1.597671000
C       -2.011792000      1.061351000      0.264100000
H       -0.504231000     -0.889324000      1.756720000
C       -2.420772000      2.463130000     -0.183652000
H       -2.239109000      3.194987000      0.619252000
H       -1.870809000      2.791798000     -1.076292000
H       -3.496061000      2.471135000     -0.421273000
C       -2.847778000      0.602977000      1.456126000
H       -2.637060000     -0.435809000      1.745290000
H       -2.666505000      1.251446000      2.328022000
H       -3.916347000      0.669376000      1.198654000
C        0.268859000     -1.069129000     -1.431624000
H       -0.181131000     -1.303578000     -2.409898000
H        1.240446000     -0.588521000     -1.619780000
C        0.462331000     -2.340101000     -0.627355000
C       -0.643363000     -3.147963000     -0.314809000
C        1.730444000     -2.716338000     -0.168509000
C       -0.481142000     -4.306457000      0.448103000
C        1.896548000     -3.879053000      0.593412000
C        0.790688000     -4.674946000      0.905827000
H       -1.635913000     -2.860992000     -0.672684000
H        2.595338000     -2.089242000     -0.403534000
H       -1.349248000     -4.927217000      0.685316000
H        2.892092000     -4.159722000      0.947410000
H        0.917405000     -5.581474000      1.503386000
H        0.535692000      2.714059000      2.115347000
H        0.567824000      1.665199000     -2.081196000

TS1-Zn-T1, G = -4584.617641 (i = -1524 cm-1)

C        2.348033000      1.934574000     -1.085014000
N        3.083467000      2.149578000      0.034006000
C        2.498627000      1.956066000      1.242053000
C        1.192147000      1.534818000      1.372995000
C        0.413853000      1.247278000      0.216542000
C        1.042153000      1.494689000     -1.037547000
H        2.855344000      2.112918000     -2.035609000
H        3.122633000      2.140014000      2.118014000
C       -0.921961000      0.759374000      0.340524000
C       -1.731561000      0.997533000      1.558094000
H       -1.583994000      1.950629000      2.075109000
H       -1.862167000      0.128136000      2.220895000
N       -1.494340000     -0.017961000     -0.668015000
C       -2.885119000     -0.058520000     -0.868749000
O       -3.406423000     -0.928064000     -1.551317000
C       -3.669687000      1.027319000     -0.186765000
H       -2.991170000      1.090355000      0.903998000
C       -3.486873000      2.404115000     -0.811436000
H       -3.948854000      3.176609000     -0.177221000
H       -2.425734000      2.659082000     -0.953114000
H       -3.979222000      2.436935000     -1.799093000
C       -5.098791000      0.643573000      0.122538000
H       -5.154354000     -0.312685000      0.664419000
H       -5.583074000      1.424460000      0.728415000
H       -5.674986000      0.531210000     -0.812538000
C       -0.686959000     -1.034424000     -1.384677000
H       -1.422963000     -1.752628000     -1.769979000
H       -0.185531000     -0.592788000     -2.256259000
C        0.311675000     -1.718262000     -0.481193000
C       -0.109753000     -2.305219000      0.722471000
C        1.670018000     -1.761442000     -0.818248000
C        0.812983000     -2.912673000      1.576833000
C        2.596634000     -2.373045000      0.033417000
C        2.170988000     -2.945892000      1.235182000
H       -1.168245000     -2.275750000      0.996053000
H        2.011583000     -1.294288000     -1.744468000
H        0.472478000     -3.362176000      2.513342000
H        3.654128000     -2.385001000     -0.239911000
H        2.894486000     -3.415842000      1.906323000
H        0.782252000      1.381575000      2.371632000
H        0.499957000      1.363217000     -1.972428000
Zn       5.003141000      2.656032000     -0.043814000
O        6.343698000      1.868904000     -1.214009000
O        5.678135000      4.570357000      0.534119000
S        6.084703000      1.484100000     -2.672875000
O        7.324317000      1.405260000     -3.441017000
O        4.935513000      2.195662000     -3.252821000
S        5.930375000      4.189825000      1.978142000
O        5.566142000      2.728141000      2.082863000
O        7.185640000      4.621421000      2.574136000
C        5.504669000     -0.284450000     -2.477037000
F        4.462272000     -0.331898000     -1.637855000
F        5.122855000     -0.767241000     -3.655928000
F        6.477814000     -1.044963000     -1.986702000
C        4.582211000      5.081929000      2.917964000
F        4.806804000      6.389006000      2.878622000
F        3.399196000      4.822767000      2.364093000
F        4.572956000      4.671120000      4.180498000

TS1-Zn‘-T1, G = -4584.601164 (i = -1316 cm-1)

C        3.651093000      3.214104000     -1.741554000
N        4.554404000      3.827581000     -0.961275000
C        4.410051000      3.686032000      0.365789000
C        3.394284000      2.945799000      0.962460000
C        2.450693000      2.273201000      0.144930000
C        2.601523000      2.439588000     -1.257277000
H        3.763235000      3.352037000     -2.823390000
H        5.149101000      4.193917000      0.996401000
C        1.378788000      1.516780000      0.725530000
C        0.864842000      1.754921000      2.094356000
H        1.058381000      0.965083000      2.835859000
H        0.911172000      2.778386000      2.478064000
N        0.717278000      0.531120000     -0.036192000
C       -0.614436000      0.306268000      0.103584000
O       -1.093286000     -0.768177000     -0.348862000
C       -1.410966000      1.308383000      0.862489000
H       -0.535788000      1.563563000      1.758414000
C       -1.594780000      2.646697000      0.153948000
H       -2.341449000      2.535776000     -0.648686000
H       -1.964163000      3.400742000      0.865457000
H       -0.660541000      3.015949000     -0.293599000
C       -2.658008000      0.757277000      1.520278000
H       -2.477021000     -0.210188000      2.007026000
H       -3.035194000      1.473026000      2.266259000
H       -3.459043000      0.606110000      0.776017000
C        1.508059000     -0.505682000     -0.759988000
H        0.922477000     -0.820079000     -1.630389000
H        2.430647000     -0.031495000     -1.107511000
C        1.824575000     -1.670179000      0.151022000
C        1.085295000     -2.860123000      0.094716000
C        2.851965000     -1.548298000      1.100842000
C        1.363451000     -3.908940000      0.976361000
C        3.130230000     -2.595482000      1.983206000
C        2.384160000     -3.778509000      1.923267000
H        0.276359000     -2.957358000     -0.630901000
H        3.439673000     -0.626968000      1.147197000
H        0.770635000     -4.825200000      0.928575000
H        3.933313000     -2.489814000      2.717224000
H        2.599639000     -4.597922000      2.613963000
H        3.341232000      2.870354000      2.050058000
H        1.882672000      2.007095000     -1.954299000
Zn      -2.650871000     -1.274687000     -1.371586000
O       -3.469572000      0.555868000     -2.125399000
O       -3.609777000     -2.863692000     -0.876415000
S       -2.902394000      0.334949000     -3.511736000
O       -3.785702000      0.557977000     -4.644884000
O       -2.170104000     -0.986944000     -3.434318000
S       -4.153353000     -3.220455000      0.514542000
O       -4.586630000     -2.046212000      1.278575000
O       -5.029524000     -4.387612000      0.442515000
C       -1.536702000      1.608083000     -3.679249000
F       -1.019807000      1.539231000     -4.896877000
F       -2.033124000      2.821278000     -3.472251000
F       -0.575410000      1.373030000     -2.782337000
C       -2.602257000     -3.832630000      1.378233000
F       -1.901079000     -4.621736000      0.563184000
F       -1.833903000     -2.807405000      1.747213000
F       -2.944343000     -4.524420000      2.461420000

INT1-Zn-T1, G = -4584.650749

C        1.659583000      2.002937000     -1.256639000
N        2.527763000      2.226195000     -0.240927000
C        2.122952000      1.973870000      1.027787000
C        0.871245000      1.470813000      1.317470000
C       -0.046461000      1.181154000      0.267685000
C        0.393257000      1.499041000     -1.049376000
H        2.017893000      2.237164000     -2.261128000
H        2.846262000      2.194770000      1.815834000
C       -1.330466000      0.621890000      0.533421000
C       -1.884977000      0.604780000      1.925599000
H       -1.380676000     -0.190894000      2.506801000
H       -1.719915000      1.555861000      2.451717000
N       -2.013134000     -0.062870000     -0.470512000
C       -3.427503000     -0.053927000     -0.545810000
O       -4.035697000     -1.046587000     -0.955702000
C       -4.130010000      1.177648000     -0.201495000
H       -2.958065000      0.374037000      1.938779000
C       -3.481680000      2.522831000     -0.218127000
H       -4.193451000      3.274703000     -0.597504000
H       -3.203210000      2.849947000      0.801878000
H       -2.573428000      2.552799000     -0.835074000
C       -5.588329000      1.097081000      0.095419000
H       -5.937443000      0.059444000      0.167771000
H       -5.817545000      1.629127000      1.036888000
H       -6.173455000      1.609014000     -0.692349000
C       -1.310033000     -1.082014000     -1.290302000
H       -2.092878000     -1.790852000     -1.589010000
H       -0.905387000     -0.631802000     -2.208406000
C       -0.217834000     -1.795999000     -0.531777000
C       -0.508834000     -2.471923000      0.663900000
C        1.101965000     -1.782509000     -0.998770000
C        0.504336000     -3.110938000      1.382096000
C        2.119202000     -2.425116000     -0.284637000
C        1.823234000     -3.087033000      0.910363000
H       -1.537296000     -2.489600000      1.035359000
H        1.340743000     -1.249481000     -1.921482000
H        0.265281000     -3.631744000      2.313039000
H        3.144733000     -2.394704000     -0.660442000
H        2.616793000     -3.583478000      1.474834000
H        0.618352000      1.270520000      2.358102000
H       -0.268148000      1.367554000     -1.904320000
Zn       4.366302000      2.872589000     -0.632068000
O        5.863693000      1.445328000     -1.176788000
O        5.230106000      4.386100000      0.226584000
S        5.564276000      1.536556000     -2.655245000
O        6.692583000      1.589237000     -3.571908000
O        4.497420000      2.599227000     -2.789669000
S        5.186485000      4.617590000      1.740449000
O        4.813786000      3.405620000      2.486512000
O        6.326685000      5.405302000      2.201361000
C        4.667559000     -0.062821000     -3.026822000
F        4.184859000     -0.033757000     -4.262470000
F        5.503943000     -1.084849000     -2.900019000
F        3.659053000     -0.216714000     -2.166636000
C        3.705124000      5.747984000      1.883053000
F        3.935445000      6.893631000      1.248332000
F        2.630460000      5.165499000      1.344984000
F        3.458718000      6.002100000      3.165777000

INT1-Zn-S0, G = -4584.659629

C        1.596117000      2.447802000     -1.076704000
N        2.491115000      2.247619000     -0.092570000
C        2.075739000      1.803507000      1.111402000
C        0.742622000      1.509643000      1.358245000
C       -0.183668000      1.639401000      0.315487000
C        0.247460000      2.157780000     -0.911686000
H        1.982947000      2.819729000     -2.027178000
H        2.844318000      1.679496000      1.877594000
C       -1.591452000      1.198875000      0.516982000
C       -2.416709000      1.980977000      1.477814000
H       -1.797741000      2.282293000      2.336410000
H       -2.758923000      2.909318000      0.988293000
N       -2.042563000      0.162308000     -0.123904000
C       -3.430719000     -0.400118000      0.106128000
O       -3.391012000     -1.525367000      0.664276000
C       -4.463072000      0.350697000     -0.405980000
H       -3.302717000      1.418822000      1.799641000
C       -4.340410000      1.687430000     -1.082106000
H       -4.944095000      1.704241000     -2.008367000
H       -4.730892000      2.514499000     -0.454060000
H       -3.310800000      1.950455000     -1.364491000
C       -5.856829000     -0.204866000     -0.284328000
H       -5.844408000     -1.213246000      0.153100000
H       -6.498716000      0.434295000      0.354026000
H       -6.359630000     -0.255797000     -1.269051000
C       -1.192583000     -0.715384000     -0.973927000
H       -1.832232000     -1.572301000     -1.216196000
H       -0.966669000     -0.178865000     -1.905823000
C        0.071961000     -1.179002000     -0.289933000
C        0.007405000     -1.741521000      0.993511000
C        1.315726000     -1.022047000     -0.912839000
C        1.181466000     -2.103775000      1.657767000
C        2.492129000     -1.380776000     -0.248446000
C        2.426065000     -1.912781000      1.043889000
H       -0.972219000     -1.865490000      1.463571000
H        1.366399000     -0.585719000     -1.911721000
H        1.127449000     -2.529860000      2.662939000
H        3.457727000     -1.219092000     -0.732534000
H        3.344402000     -2.178831000      1.573552000
H        0.447339000      1.123448000      2.334160000
H       -0.445762000      2.305505000     -1.740853000
Zn       4.454554000      2.402116000     -0.486600000
O        5.220384000      0.553393000     -1.356642000
O        5.771081000      3.227862000      0.673418000
S        5.315047000      1.172893000     -2.729263000
O        6.553886000      0.999469000     -3.471096000
O        4.795751000      2.589915000     -2.563619000
S        5.740652000      3.191457000      2.206205000
O        4.958113000      2.062547000      2.732353000
O        7.057536000      3.461174000      2.775681000
C        3.995304000      0.306379000     -3.743192000
F        4.176670000      0.576485000     -5.027273000
F        4.077909000     -1.003474000     -3.546014000
F        2.784562000      0.726397000     -3.374494000
C        4.710889000      4.707729000      2.576950000
F        5.305740000      5.797467000      2.101868000
F        3.505794000      4.593002000      2.010223000
F        4.555799000      4.832221000      3.891787000

TS2-Zn-S0, G = -4584.640793 (i = -102 cm-1)

C        0.738327000      1.119955000     -1.052069000
N        1.577409000      1.305923000     -0.010159000
C        1.123868000      1.077231000      1.243385000
C       -0.154313000      0.606041000      1.489914000
C       -1.009384000      0.292346000      0.407512000
C       -0.539718000      0.623406000     -0.886061000
H        1.131932000      1.346452000     -2.044034000
H        1.830987000      1.265385000      2.054243000
C       -2.284104000     -0.370574000      0.596092000
C       -2.869680000     -0.446905000      1.981177000
H       -2.140846000     -0.920734000      2.665826000
H       -3.098270000      0.548719000      2.392718000
N       -2.750449000     -1.146760000     -0.434742000
C       -4.148735000     -1.171133000     -0.564056000
O       -4.784812000     -2.204802000     -0.821663000
C       -4.745904000      0.119910000     -0.317537000
H       -3.788013000     -1.045468000      1.993229000
C       -4.187491000      1.441246000     -0.746279000
H       -4.840305000      1.870106000     -1.532146000
H       -4.203542000      2.168862000      0.085375000
H       -3.166364000      1.387001000     -1.138449000
C       -6.188884000      0.121064000      0.052526000
H       -6.498810000     -0.829341000      0.508347000
H       -6.409308000      0.961425000      0.733171000
H       -6.819957000      0.283224000     -0.845151000
C       -1.958620000     -2.191942000     -1.133530000
H       -2.647868000     -3.050446000     -1.179649000
H       -1.754623000     -1.878531000     -2.168484000
C       -0.682845000     -2.556753000     -0.418008000
C       -0.724216000     -3.093318000      0.878410000
C        0.562946000     -2.307944000     -1.008299000
C        0.457722000     -3.344239000      1.579543000
C        1.749368000     -2.553541000     -0.309631000
C        1.697434000     -3.064790000      0.991028000
H       -1.689989000     -3.300958000      1.347070000
H        0.603887000     -1.890927000     -2.015947000
H        0.412368000     -3.755565000      2.591241000
H        2.711208000     -2.324254000     -0.773569000
H        2.621262000     -3.247638000      1.545831000
H       -0.451037000      0.423092000      2.521467000
H       -1.164269000      0.465310000     -1.764964000
Zn       3.515406000      1.613015000     -0.352636000
O        4.466403000     -0.241200000     -1.089090000
O        4.764712000      2.562371000      0.786611000
S        4.632133000      0.340293000     -2.468791000
O        5.950602000      0.282966000     -3.082105000
O        3.951854000      1.697243000     -2.419880000
S        4.624400000      2.838157000      2.285686000
O        3.803937000      1.834014000      2.979292000
O        5.899650000      3.232038000      2.878278000
C        3.531985000     -0.701682000     -3.569942000
F        3.718887000     -0.358962000     -4.836271000
F        3.839028000     -1.983058000     -3.404866000
F        2.250516000     -0.518048000     -3.250832000
C        3.577823000      4.386878000      2.265885000
F        4.200634000      5.356687000      1.602508000
F        2.407689000      4.140225000      1.669721000
F        3.347979000      4.788930000      3.512754000

b19’-Zn-S0, G = -4584.707275

C        1.120972000      3.441977000     -0.888707000
N        1.874372000      3.954366000      0.101494000
C        1.627643000      3.585039000      1.373644000
C        0.651268000      2.650588000      1.688280000
C       -0.122547000      2.076661000      0.667410000
C        0.110962000      2.523276000     -0.641104000
H        1.358637000      3.764858000     -1.904350000
H        2.238328000      4.062570000      2.143325000
C       -1.163466000      1.022171000      0.976173000
C       -0.661166000      0.044800000      2.044259000
H        0.348787000     -0.314855000      1.805678000
H       -0.633328000      0.528719000      3.030640000
N       -1.733192000      0.372801000     -0.226145000
C       -3.010474000      0.802588000     -0.035408000
O       -4.032801000      0.597029000     -0.658727000
C       -2.643106000      1.606158000      1.225704000
H       -1.329083000     -0.822380000      2.107900000
C       -2.803399000      3.115423000      1.048206000
H       -3.874514000      3.370060000      1.044753000
H       -2.324047000      3.656808000      1.879278000
H       -2.367095000      3.477543000      0.106737000
C       -3.346419000      1.131122000      2.493643000
H       -3.318654000      0.039028000      2.608924000
H       -2.891188000      1.588915000      3.386063000
H       -4.403033000      1.438633000      2.455359000
C       -1.190560000     -0.490869000     -1.270890000
H       -2.002082000     -1.176348000     -1.560114000
H       -0.936554000      0.106797000     -2.162863000
C        0.025552000     -1.259940000     -0.812965000
C       -0.114380000     -2.471276000     -0.120179000
C        1.311829000     -0.744105000     -1.027078000
C        1.008975000     -3.145746000      0.364435000
C        2.438481000     -1.412767000     -0.538418000
C        2.288378000     -2.613869000      0.162477000
H       -1.113949000     -2.881733000      0.048060000
H        1.435870000      0.191989000     -1.575013000
H        0.887190000     -4.088876000      0.903424000
H        3.432500000     -0.991976000     -0.708368000
H        3.167146000     -3.139408000      0.545092000
H        0.510030000      2.374705000      2.732736000
H       -0.474415000      2.144691000     -1.477215000
Zn       3.450252000      5.126916000     -0.341954000
O        5.237080000      3.938557000     -0.631907000
O        3.812888000      6.852841000      0.445395000
S        4.984445000      3.782442000     -2.111598000
O        6.107813000      3.944890000     -3.020613000
O        3.735338000      4.593515000     -2.394852000
S        3.508972000      7.204866000      1.908630000
O        3.448070000      6.021442000      2.779083000
O        4.279794000      8.364617000      2.347624000
C        4.423233000      1.999962000     -2.296405000
F        3.783558000      1.846416000     -3.448889000
F        5.475930000      1.194839000     -2.260707000
F        3.599271000      1.683276000     -1.295756000
C        1.740903000      7.799214000      1.776101000
F        1.660293000      8.823157000      0.931694000
F        0.954401000      6.813652000      1.335495000
F        1.312117000      8.186845000      2.974208000

a1-S0, G = -997.83370912

C        1.073222000      2.000575000     -1.699650000
C        1.291313000      3.298481000     -1.237317000
C        0.793926000      3.636044000      0.027736000
N        0.132048000      2.783030000      0.807403000
C       -0.074352000      1.527696000      0.370645000
C        0.379151000      1.101413000     -0.888928000
H        1.440183000      1.684493000     -2.679190000
H        1.831172000      4.036337000     -1.834459000
H        0.942770000      4.647793000      0.423572000
H        0.209985000      0.080808000     -1.225629000
C       -0.813550000      0.609292000      1.283569000
C       -1.292043000      1.000566000      2.473890000
H       -1.147343000      2.027893000      2.809393000
H       -1.820824000      0.295494000      3.118487000
N       -0.943916000     -0.760738000      0.876325000
C       -1.860002000     -1.191863000     -0.049233000
O       -1.880048000     -2.357350000     -0.441705000
C        0.038326000     -1.718487000      1.416982000
H        0.146564000     -1.519283000      2.492006000
H       -0.391147000     -2.717969000      1.280129000
C        1.376622000     -1.612093000      0.721260000
C        2.404046000     -0.827862000      1.262952000
C        1.574146000     -2.226731000     -0.525806000
C        3.605428000     -0.649503000      0.569014000
C        2.772914000     -2.050248000     -1.221178000
C        3.790741000     -1.257289000     -0.676769000
H        2.254656000     -0.341552000      2.230996000
H        0.768534000     -2.827417000     -0.954869000
H        4.396911000     -0.031021000      1.000413000
H        2.914662000     -2.531515000     -2.192541000
H        4.727723000     -1.116865000     -1.222053000
C       -2.893066000     -0.182703000     -0.531122000
C       -3.139027000     -0.324395000     -2.040337000
C       -4.201516000     -0.380696000      0.263862000
H       -2.540009000      0.839684000     -0.332627000
C       -4.236811000      0.630261000     -2.518753000
H       -3.428820000     -1.367652000     -2.248287000
H       -2.200033000     -0.138965000     -2.588449000
C       -5.297395000      0.572084000     -0.222961000
H       -4.530228000     -1.427320000      0.138395000
H       -4.007659000     -0.230956000      1.338831000
C       -5.535982000      0.431302000     -1.730201000
H       -4.415822000      0.486340000     -3.597477000
H       -3.892007000      1.673363000     -2.393511000
H       -6.230431000      0.386589000      0.335188000
H       -4.995937000      1.611819000      0.001222000
H       -6.302844000      1.149859000     -2.065222000
H       -5.933973000     -0.578608000     -1.940568000
```

# 5. Transformations of the Products

**2-(1-Benzyl-2,3,3-trimethylazetidin-2-yl)pyridine (c1)**

Charge a round bottom flask equipped with a magnetic stir bar with **b9** (56.1 mg, 0.2 mmol, 1.0 equiv.) and THF (4mL) under an argon atmosphere and cool the mixture at 0°C. Then added LiAlH_4_ (0.24 ml, 0.24 mmol, 1M in THF) slowly and stirred the reaction mixture at room temperature for 2 h. Quench the reaction by adding saturated NH_4_Cl (aq.) at 0°C. Filter the mixture and extract the aqueous layer with EtOAc (3 x 10 mL), wash the combined organic layers with brine (2 x 10 mL). Dry the combined organic extracts with Na_2_SO_4_, filtered and concentrated in vacuo. The product was purified by column chromatography (silica, PE/EtOAc = 4/1) to obtain **c1** (49.0 mg, 0.184 mmol, 92%) as white solid.

**^1^H NMR** (400 MHz, CDCl_3_): δ [ppm] = 8.64 (d, *J* = 4.1 Hz, 1H), 7.69 (td, *J* = 7.8, 1.4 Hz, 1H), 7.36 – 7.17 (m, 7H), 3.99 (d, *J* = 10.7 Hz, 1H), 3.62 (d, *J* = 11.3 Hz, 1H), 3.26 (d, *J* = 10.7 Hz, 1H), 3.06 (d, *J* = 11.3 Hz, 1H), 1.76 (s, 3H), 0.99 (s, 3H), 0.60 (s, 3H).

**^13^C NMR** (101 MHz, CDCl_3_): δ [ppm] = 161.2, 148.0, 139.6, 135.8, 128.7, 127.4, 123.3, 121.8, 110.1, 71.9, 66.3, 47.9, 40.6, 22.6, 19.9, 19.0.

**HRMS (ESI)** *m/z*: [M+H] ^+^ calculated for C_18_H_23_N_2_: 267.1856, found: 267.1853.

**2-(1-Benzyl-2,3,3-trimethyl-4-oxoazetidin-2-yl)-1l4-pyridin-1-olate (c2)**

According to a literature procedure^[14]^, *m*-Chloroperoxybenzoic acid (41.3 mg, 0.24 mmol, 1.2 equiv) was added portion-wise to a stirred **b9** (56.1 mg, 0.2 mmol, 1.0 equiv) in dichloromethane (5 mL) at 0 °C, and the stirring was continued at this temperature for a further 12 h. Then, the mixture was poured into a saturated aqueous NaHCO_3_ (15 mL), extracted with dichloromethane (30 mL), and the organic layer was washed with NaHCO_3_ (aq), dried with Na2SO4, filtered and concentration in vacuo. The crude product was purified by column chromatography (PE/EA = 2/1) to afford **c2** (53.3 mg, 0.180 mmol, 90%) as white solid.

**^1^H NMR** (400 MHz, CDCl_3_): δ [ppm] = 8.15 (d, *J* = 6.3 Hz, 1H), 7.45 – 7.29 (m, 5H), 7.17 – 7.10 (m, 1H), 7.04 – 6.93 (m, 2H), 4.63 (d, *J* = 15.5 Hz, 1H), 4.37 (d, *J* = 15.5 Hz, 1H), 1.81 (s, 3H), 1.47 (s, 3H), 1.08 (s, 3H).

**^13^C NMR** (101 MHz, CDCl_3_): δ [ppm] = 176.1, 153.1, 140.8, 137.6, 129.1, 128.6, 128.0, 125.4, 124.6, 124.0, 69.8, 59.1, 44.5, 18.9, 17.9, 16.4.

**HRMS (ESI)** *m/z*: [M+H] ^+^ calculated for C_18_H_21_N_2_O_2_:297.1598, found: 297.1599.

1-Benzyl-3,3,4-trimethyl-4-(pyridin-2-yl)azetidine-2-thione **(c3)**

Charge a round bottom flask equipped with a magnetic stir bar with **b9** (56.1 mg, 0.2 mmol, 1.0 equiv.), and Lawesson's Reagent (80.8 mg, 0.2 mmol, 1.0 equiv.) to a flame-dried Schlenk tube. After evacuated/backfilled with Argon (3 times), added the THF (4 mL). After stirring the reaction mixture for 2 hours at 80 °C, EtOAc (30 mL) and water (10 mL) were added and extracted with EtOAc. The organic extracts were combined, washed with NaCl (aq.), dried over Na_2_SO_4_, filtered and the solvent was removed under reduced pressure and the crude product was purified by column chromatography (PE/EA = 5/1) to afford **c3** (55.7 mg, 0.184 mmol, 94%) as pale colorless liquid.

**^1^H NMR** (400 MHz, CDCl_3_): δ [ppm] = 8.61 (d, *J* = 4.7 Hz, 1H), 7.63 (td, *J* = 7.8, 1.7 Hz, 1H), 7.41 (dd, *J* = 7.3, 1.4 Hz, 2H), 7.34 – 7.23 (m, 3H), 7.23 – 7.15 (m, 1H), 7.04 (d, *J* = 8.0 Hz, 1H), 5.37 (d, *J* = 14.8 Hz, 1H), 4.52 (d, *J* = 14.7 Hz, 1H), 1.49 (s, 3H), 1.35 (s, 3H), 0.74 (s, 3H).

**^13^C NMR** (101 MHz, CDCl_3_): δ [ppm] = 210.8, 160.1, 149.3, 136.5, 129.1, 128.8, 128.0, 122.4, 120.8, 78.7, 59.6, 47.2, 21.6, 20.9, 20.6.

**HRMS (ESI)** *m/z*: [M+Na] ^+^ calculated for C_18_H_20_N_2_SNa^+^: 319.1239, found: 319.1235.

**3-(Pyridin-2-yl)-2-azaspiro[3.5]nonan-1-one (c4)**

Charge a round bottom flask equipped with a magnetic stir bar with **b26** (73.6 mg, 0.2 mmol, 1.0 equiv.) and MeCN (2mL) under an argon atmosphere and cool the mixture at 0°C. Then added Ammonium ceric nitrate (530 mg, 1.0 mmol) in 0.5 mL H_2_O, slowly and stirred the reaction mixture at room temperature for 12 h. The reaction was monitored by TLC, evaporate the solvent by rotary evaporation. Filtered the mixture and extracted the aqueous layer with EtOAc (3 x 10 mL), washed the combined organic layers with brine (2 x 10 mL). Dried the combined organic extracts with Na_2_SO_4_, filtered and concentrated in vacuo. The product was purified by column chromatography (silica, PE/EtOAc = 1/1) to obtain **c4** (18.4 mg, 0.080 mmol, 40%) as white solid.

**^1^H NMR** (400 MHz, CDCl_3_): δ [ppm] = 8.60 (d, *J* = 4.2 Hz, 1H), 7.69 (t, *J* = 7.7 Hz, 1H), 7.39 (d, *J* = 7.9 Hz, 1H), 7.23 – 7.14 (m, 1H), 2.25 – 2.14 (m, 1H), 1.86 – 1.75 (m, 2H), 1.72 (s, 3H), 1.67 – 1.54 (m, 3H), 1.43 – 1.37 (m, 1H), 1.18 – 1.14 (m, 1H), 1.06 – 0.96 (m, 1H), 0.91 – 0.81 (m, 1H).

**^13^C NMR** (101 MHz, CDCl_3_): δ [ppm] = 172.4, 149.2, 136.4, 122.1, 120.2, 61.6, 57.5, 31.3, 29.1, 25.6, 23.6, 23.2, 22.8.

**HRMS (ESI)** *m/z*: [M+Na] ^+^ calculated for C_14_H_18_N_2_ONa^+^:253.1311, found:253.1306.

# 6. X-Ray Crystallographic Details

The single crystal cultivation method is as follows: weigh the compound (30 mg) and dissolve it in a mixture system (DCM/PE = 5:1) that dichloromethane (DCM) as a benign solvent and petroleum ether (PE) as a poor solvent, the system was then sealed with a parafilm, and an appropriate amount of small holes were pricked with a needle, and it was slowly volatilized at room temperature until crystals were precipitated. The X-ray data of **b10** is deposited in the Cambridge Crystallographic Data Centre with a number of **CCDC** **2496582**; for **b24**, **CCDC** **2496581**; for **b29**, **CCDC** **2554983**; for **b43**, **CCDC** **2496583.**

**6.1 X-Ray Crystallographic Details of b10**

| **Crystal data and structure refinement for 20250715-Xiong Yang-HYA-5-7-R-P-200K.** | |
| --- | --- |
| Identification code | 20250715-Xiong Yang-HYA-5-7-R-P-200K |
| Empirical formula | C_23_H_22_N_2_O |
| Formula weight | 342.42 |
| Temperature/K | 200.00(10) |
| Crystal system | monoclinic |
| Space group | Cc |
| a/Å | 12.6893(3) |
| b/Å | 10.3751(2) |
| c/Å | 14.0918(3) |
| α/° | 90 |
| β/° | 95.429(2) |
| γ/° | 90 |
| Volume/Å^3^ | 1846.90(7) |
| Z | 4 |
| ρ_calc_g/cm^3^ | 1.231 |
| μ/mm^‑1^ | 0.592 |
| F(000) | 728.0 |
| Crystal size/mm^3^ | 0.27 × 0.25 × 0.24 |
| Radiation | Cu Kα (λ = 1.54184) |
| 2Θ range for data collection/° | 11.036 to 140.208 |
| Index ranges | -15 ≤ h ≤ 12, -12 ≤ k ≤ 11, -17 ≤ l ≤ 16 |
| Reflections collected | 4071 |
| Independent reflections | 2334 [R_int_ = 0.0156, R_sigma_ = 0.0182] |
| Data/restraints/parameters | 2334/2/237 |
| Goodness-of-fit on F^2^ | 1.103 |
| Final R indexes [I>=2σ (I)] | R_1_ = 0.0493, wR_2_ = 0.1380 |
| Final R indexes [all data] | R_1_ = 0.0497, wR_2_ = 0.1390 |
| Largest diff. peak/hole / e Å^-3^ | 0.41/-0.41 |
| Flack parameter | -0.11(19) |

**6.2 X-Ray Crystallographic Details of b24**

| **Crystal data and structure refinement for 20240513-Xiong Yang-HYA-4-57-P-200K.** | |
| --- | --- |
| Identification code | 20240513-Xiong Yang-HYA-4-57-P-200K |
| Empirical formula | C_21_H_23_N_2_OBr |
| Formula weight | 399.32 |
| Temperature/K | 200.00(10) |
| Crystal system | triclinic |
| Space group | P-1 |
| a/Å | 9.3994(4) |
| b/Å | 10.6005(4) |
| c/Å | 11.2469(4) |
| α/° | 65.996(4) |
| β/° | 66.530(4) |
| γ/° | 69.809(4) |
| Volume/Å^3^ | 916.31(7) |
| Z | 2 |
| ρ_calc_g/cm^3^ | 1.447 |
| μ/mm^‑1^ | 3.133 |
| F(000) | 412.0 |
| Crystal size/mm^3^ | 0.32 × 0.26 × 0.24 |
| Radiation | Cu Kα (λ = 1.54184) |
| 2Θ range for data collection/° | 9.026 to 140.168 |
| Index ranges | -11 ≤ h ≤ 11, -12 ≤ k ≤ 12, -12 ≤ l ≤ 13 |
| Reflections collected | 7673 |
| Independent reflections | 3470 [R_int_ = 0.0142, R_sigma_ = 0.0143] |
| Data/restraints/parameters | 3470/0/227 |
| Goodness-of-fit on F^2^ | 1.075 |
| Final R indexes [I>=2σ (I)] | R_1_ = 0.0301, wR_2_ = 0.0784 |
| Final R indexes [all data] | R_1_ = 0.0313, wR_2_ = 0.0795 |
| Largest diff. peak/hole / e Å^-3^ | 0.30/-0.80 |

**6.3 X-Ray Crystallographic Details of b29**

| **Crystal data and structure refinement for 20260228-Xiong Yang-HYA-b29.** | |
| --- | --- |
| Identification code | 20260228-Xiong Yang-HYA-b30 |
| Empirical formula | C_22_H_20_N_2_O |
| Formula weight | 328.40 |
| Temperature/K | 293(2) |
| Crystal system | monoclinic |
| Space group | P2_1_/c |
| a/Å | 10.0287(8) |
| b/Å | 9.2002(6) |
| c/Å | 19.7143(11) |
| α/° | 90 |
| β/° | 90.857(6) |
| γ/° | 90 |
| Volume/Å^3^ | 1818.8(2) |
| Z | 4 |
| ρ_calc_g/cm^3^ | 1.199 |
| μ/mm^‑1^ | 0.581 |
| F(000) | 696.0 |
| Crystal size/mm^3^ | 0.21 × 0.18 × 0.16 |
| Radiation | Cu Kα (λ = 1.54184) |
| 2Θ range for data collection/° | 8.818 to 140.802 |
| Index ranges | -11 ≤ h ≤ 12, -8 ≤ k ≤ 11, -23 ≤ l ≤ 24 |
| Reflections collected | 9041 |
| Independent reflections | 3434 [R_int_ = 0.0445, R_sigma_ = 0.0390] |
| Data/restraints/parameters | 3434/105/283 |
| Goodness-of-fit on F^2^ | 1.039 |
| Final R indexes [I>=2σ (I)] | R_1_ = 0.1048, wR_2_ = 0.3139 |
| Final R indexes [all data] | R_1_ = 0.1230, wR_2_ = 0.3316 |
| Largest diff. peak/hole / e Å^-3^ | 0.45/-0.39 |

**6.4 X-Ray Crystallographic Details of b43**

| **Crystal data and structure refinement for 20250709-Xiong Yang-HYA-5-21-P.** | |
| --- | --- |
| Identification code | 20250709-Xiong Yang-HYA-5-21-P |
| Empirical formula | C_24_H_30_N_2_O |
| Formula weight | 362.50 |
| Temperature/K | 293(2) |
| Crystal system | monoclinic |
| Space group | P2_1_/n |
| a/Å | 15.7908(4) |
| b/Å | 13.6929(3) |
| c/Å | 20.7130(6) |
| α/° | 90 |
| β/° | 108.108(3) |
| γ/° | 90 |
| Volume/Å^3^ | 4256.8(2) |
| Z | 8 |
| ρ_calc_g/cm^3^ | 1.131 |
| μ/mm^‑1^ | 0.531 |
| F(000) | 1568.0 |
| Crystal size/mm^3^ | 0.23 × 0.21 × 0.2 |
| Radiation | Cu Kα (λ = 1.54184) |
| 2Θ range for data collection/° | 6.196 to 140.298 |
| Index ranges | -17 ≤ h ≤ 19, -16 ≤ k ≤ 14, -25 ≤ l ≤ 23 |
| Reflections collected | 26069 |
| Independent reflections | 8048 [R_int_ = 0.0253, R_sigma_ = 0.0198] |
| Data/restraints/parameters | 8048/0/491 |
| Goodness-of-fit on F^2^ | 1.025 |
| Final R indexes [I>=2σ (I)] | R_1_ = 0.0498, wR_2_ = 0.1393 |
| Final R indexes [all data] | R_1_ = 0.0688, wR_2_ = 0.1606 |
| Largest diff. peak/hole / e Å^-3^ | 0.15/-0.15 |

# 7. References

[1] X.-H. Chang, Z.-L. Wang, M. Zhao, C. Yang, J.-J. Li, W.-W. Ma, Y.-H. Xu, Synthesis of Functionalized Vinylsilanes via Metal-Free Dehydrogenative Silylation of Enamides. *Org. Lett.* 22 (2020), 1326-1330.
[2] K. Tanaka, M. Takahashi, H. Imase, T. Osaka, K. Noguchi, M. Hirano, Enantioselective synthesis of α,α-disubstituted α-amino acids by Rh-catalyzed [2+2+2] cycloaddition of 1,6-diynes with protected dehydroamino acid. *Tetrahedron* 64 (2008), 6289-6293.
[3] R.-Y. Guo, S. Adak, P. Bellotti, X. Gao, W. W. Smith, S. N. Le, J.-J. Ma, K. N. Houk, F. Glorius, S.-M Chen, M. K. Brown, Photochemical dearomative cyclo-additions of quinolines and alkenes: scope and mechanism studies. *J. Am. Chem. Soc*. 144 (2022), 17680-17691.
[4] J.-J. Huang, T.-P. Zhou, N.-N. Sun, H.-B. Yu, X.-X. Yu, R.-Z. Liao, W.-J. Yao, Z.-F. Da, G.-J. Wu, F.-R. Zhong, Accessing ladder-shape azetidine-fused indoline pentacycles through intermolecular regiodivergent aza-Paternò–Büchi reactions. *Nat. Commun.* 15 (2024), 1431-1441.
[5] J. D. Chai, M. Head-Gordon, Long-range corrected hybrid density functionals with damped atom–atom dispersion corrections. *Phys. Chem. Chem. Phy*s. 10 (2008), 6615-6620.
[6] F. Weigend, R. Ahlrichs, Balanced basis sets of split valence, triple zeta valence and quadruple zeta valence quality for H to Rn: Design and assessment of accuracy. *Phys. Chem. Chem. Phys*. 7 (2005), 3297-3305.
[7] S. Miertuš, E. Scrocco, J. Tomasi, Electrostatic interaction of a solute with a continuum. A direct utilizaion of AB initio molecular potentials for the prevision of solvent effects. *Chem. Phys*. 55 (1981), 117-129.
[8] Jr, J. W. McIver, A. Komornicki, Structure of transition states in organic reactions. General theory and an application to the cyclobutene-butadiene isomerization using a semiempirical molecular orbital method. *J. Am. Chem. Soc.* 94 (1972), 2625-2633.
[9] C. Gonzalez, H. B. Schlegel, Reaction path following in mass-weighted internal coordinates. *J. Phys. Chem*. 94 (1990), 5523-5527.
[10] S. Grimme, Supramolecular binding thermodynamics by dispersion‐corrected density functional theory. *Chem. Eur. J*. 18 (2012), 9955-9964.
[11] G. Luchini, J. V. Alegre-Requena, I. Funes-Ardoiz, R. S. Paton, GoodVibes: automated thermochemistry for heterogeneous computational chemistry data. *F1000Research* 9 (2020), 291.
[12] Q. Liu, Y. Huang, X. Zhou, I. Fernández, Y. Xiong, Visible Light-Mediated [4+2] Annulation of Silylimines with Olefins to 1-Aminotetralins Enabled by Diradical Hydrogen Atom Transfer of C-H Bonds. *Angew. Chem. Int. Ed*. 64 (2024), e202421464.
[13] Casida, M. E. *Recent Developments and Applications of Modern Density Functional Theory*, 4, Elsevier, Amsterdam (1996).
[14] A. V. Malkov, M. Orsini, D. Pernazza, W. Muir, K. V. Langer, P. Meghani, P. Kočovský, Chiral 2,2’-Bipyridine-Type *N*-Monoxides as organocatalysts in the enantioselective allylation of aldehydes with allyltrichlorosilane. *Org. Lett*. 4 (2002), 1047-1049.

# 8. NMR-Spectra of Compounds

## 8.1 NMR-Spectra of Enamides

**a1-^1^H NMR** (400 MHz, CDCl_3_)

**a1-^13^C NMR** (101 MHz, CDCl_3_)

**a2-^1^H NMR** (400 MHz, CDCl_3_)

**a2-^13^C NMR** (101 MHz, CDCl_3_)

**a3-^1^H NMR** (400 MHz, CDCl_3_)

**a3-^13^C NMR** (101 MHz, CDCl_3_)

**a4-^1^H NMR** (400 MHz, CDCl_3_)

**a4-^13^C NMR** (101 MHz, CDCl_3_)

**a4-^19^F NMR** (564 MHz, CDCl_3_)

**a5-^1^H NMR** (400 MHz, CDCl_3_)

**a5-^13^C NMR** (101 MHz, CDCl_3_)

**a6-^1^H NMR** (400 MHz, CDCl_3_)

**a6-^13^C NMR** (101 MHz, CDCl_3_)

**a7-^1^H NMR** (400 MHz, CDCl_3_)

**a7-^13^C NMR** (101 MHz, CDCl_3_)

**a8-^1^H NMR** (400 MHz, CDCl_3_)

**a8-^13^C NMR** (101 MHz, CDCl_3_)

**a9-^1^H NMR** (400 MHz, CDCl_3_)

**a9-^13^C NMR** (101 MHz, CDCl_3_)

**a10-^1^H NMR** (400 MHz, CDCl_3_)

**a10-^13^C NMR** (101 MHz, CDCl_3_)

**a11-^1^H NMR** (400 MHz, CDCl_3_)

**a11-^13^C NMR (101 MHz, CDCl_3_)**

**a12-^1^H NMR** (400 MHz, CDCl_3_)

**a12-^13^C NMR** (101 MHz, CDCl_3_)

**a13-^1^H NMR** (400 MHz, CDCl_3_)

**a13-^13^C NMR** (101 MHz, CDCl_3_)

**a14-^1^H NMR** (400 MHz, CDCl_3_)

**a14-^13^C NMR** (101 MHz, CDCl_3_)

**a14^-19^F NMR** (564 MHz, CDCl_3_)

**a15-^1^H NMR** (400 MHz, CDCl_3_)

**a15-^13^C NMR** (101 MHz, CDCl_3_)

**\**

**a16-^1^H NMR** (400 MHz, CDCl_3_)

**a16-^13^C NMR** (101 MHz, CDCl_3_)

**a17-^1^H NMR** (400 MHz, CDCl_3_)

**a17-^13^C NMR** (101 MHz, CDCl_3_)

**a17-^19^F NMR** (564 MHz, CDCl_3_)

**a18-^1^H NMR** (400 MHz, CDCl_3_)

**a18-^13^C NMR** (101 MHz, CDCl_3_)

**a19-^1^H NMR** (400 MHz, CDCl_3_)

**a19-^13^C NMR** (101 MHz, CDCl_3_)

**a20-^1^H NMR** (400 MHz, CDCl_3_)

**a20-^13^C NMR** (101 MHz, CDCl_3_)

**a21-^1^H NMR** (400 MHz, CDCl_3_)

**a21-^13^C NMR** (101 MHz, CDCl_3_)

**a22-^1^H NMR** (400 MHz, CDCl_3_)

**a22-^13^C NMR** (101 MHz, CDCl_3_)

**a22-^19^F NMR** (564 MHz, CDCl_3_)

**a23-^1^H NMR** (400 MHz, CDCl_3_)

**a23-^13^C NMR** (101 MHz, CDCl_3_)

**a24-^1^H NMR** (400 MHz, CDCl_3_)

**a24-^13^C NMR** (101 MHz, CDCl_3_)

**a25-^1^H NMR** (400 MHz, CDCl_3_)

**a25-^13^C NMR** (101 MHz, CDCl_3_)

**A25-^19^F NMR** (564 MHz, CDCl_3_)

**a26-^1^H NMR** (400 MHz, CDCl_3_)

**a26-^13^C NMR** (101 MHz, CDCl_3_)

**a26-^19^F NMR** (564 MHz, CDCl_3_)

**a27-^1^H NMR** (400 MHz, CDCl_3_)

**a27-^13^C NMR** (101 MHz, CDCl_3_)

**a28-^1^H NMR** (400 MHz, CDCl_3_)

**a28-^13^C NMR** (101 MHz, CDCl_3_)

**a29-^1^H NMR** (400 MHz, CDCl_3_)

**a29-^13^C NMR** (101 MHz, CDCl_3_)

**a30-^1^H NMR** (400 MHz, CDCl_3_)

**a30-^13^C NMR** (101 MHz, CDCl_3_)

**a31-^1^H NMR** (400 MHz, CDCl_3_)

**a31-^13^C NMR** (101 MHz, CDCl_3_)

**a31-^19^F NMR** (564 MHz, CDCl_3_)

**a32-^1^H NMR** (400 MHz, CDCl_3_)

**a32-^13^C NMR** (101 MHz, CDCl_3_)

**a33-^1^H NMR** (400 MHz, CDCl_3_)

**a33-^13^C NMR** (101 MHz, CDCl_3_)

**a34-^1^H NMR** (400 MHz, CDCl_3_)

**a34-^13^C NMR** (101 MHz, CDCl_3_)

**a35-^1^H NMR** (400 MHz, CDCl_3_)

**a35-^13^C NMR** (101 MHz, CDCl_3_)

**a36-^1^H NMR** (400 MHz, CDCl_3_)

**a36-^13^C NMR** (101 MHz, CDCl_3_)

**a37-^1^H NMR** (400 MHz, CDCl_3_)

**a37-^13^C NMR** (101 MHz, CDCl_3_)

**a38-^1^H NMR** (400 MHz, CDCl_3_)

**a38-^13^C NMR** (101 MHz, CDCl_3_)

**a39-^1^H NMR** (400 MHz, CDCl_3_)

**a39-^13^C NMR** (101 MHz, CDCl_3_)

**a40-^1^H NMR** (400 MHz, CDCl_3_)

**a40-^13^C NMR** (101 MHz, CDCl_3_)

**a41-^1^H NMR** (400 MHz, CDCl_3_)

**a41-^13^C NMR** (101 MHz, CDCl_3_)

**a42-^1^H NMR** (400 MHz, CDCl_3_)

**a42-^13^C NMR** (101 MHz, CDCl_3_)

**a43-^1^H NMR** (400 MHz, CDCl_3_)

**a43-^13^C NMR** (101 MHz, CDCl_3_)

**a44-^1^H NMR** (400 MHz, CDCl_3_)

**a44-^13^C NMR** (101 MHz, CDCl_3_)

**a45-^1^H NMR** (400 MHz, CDCl_3_)

**a45-^13^C NMR** (101 MHz, CDCl_3_)

**a46-^1^H NMR** (400 MHz, CDCl_3_)

**a46-^13^C NMR** (101 MHz, CDCl_3_)

**a47-^1^H NMR** (400 MHz, CDCl_3_)

**a47-^13^C NMR** (101 MHz, CDCl_3_)

**a48-^1^H NMR** (400 MHz, CDCl_3_)

**a48-^13^C NMR** (101 MHz, CDCl_3_)

**a49-^1^H NMR** (400 MHz, CDCl_3_)

**a49-^13^C NMR** (101 MHz, CDCl_3_)

**a50-^1^H NMR** (400 MHz, CDCl_3_)

**a50-^13^C NMR** (101 MHz, CDCl_3_)

**a51-^1^H NMR** (400 MHz, CDCl_3_)

**a51-^13^C NMR** (101 MHz, CDCl_3_)

## 8.2 NMR-Spectra of Products of Photochemical Reactions

**b1-^1^H NMR** (400 MHz, CDCl_3_)

**b1-^13^C NMR** (101 MHz, CDCl_3_)

**b2-^1^H NMR** (400 MHz, CDCl_3_)

**b2-^13^C NMR** (101 MHz, CDCl_3_)

**b3-^1^H NMR** (400 MHz, CDCl_3_)

**b3-^13^C NMR** (101 MHz, CDCl_3_)

**b4-^1^H NMR** (400 MHz, CDCl_3_)

**b4-^13^C NMR** (101 MHz, CDCl_3_)

**b4-^19^F NMR** (564 MHz, CDCl_3_)

**b5-^1^H NMR** (400 MHz, CDCl_3_)

**b5-^13^C NMR** (101 MHz, CDCl_3_)

**b6-^1^H NMR** (400 MHz, CDCl_3_)

**b6-^13^C NMR** (101 MHz, CDCl_3_)

**b7-^1^H NMR** (400 MHz, CDCl_3_)

**b7-^13^C NMR** (101 MHz, CDCl_3_)

**b8-^1^H NMR** (400 MHz, CDCl_3_)

Minor diastereoisomer:

**b8-^1^H NMR** (400 MHz, CDCl_3_)

**b8-^13^C NMR** (101 MHz, CDCl_3_)

**b9-^1^H NMR** (400 MHz, CDCl_3_)

**b9-^13^C NMR** (101 MHz, CDCl_3_)

**b10-^1^H NMR** (400 MHz, CDCl_3_)

Minor diastereoisomer:

**b10-^1^H NMR** (400 MHz, CDCl_3_)

**b10-^13^C NMR** (101 MHz, CDCl_3_)

**b11-^1^H NMR** (400 MHz, CDCl_3_)

**b11-^13^C NMR** (101 MHz, CDCl_3_)

**b12-^1^H NMR** (400 MHz, CDCl_3_)

Minor diastereoisomer:

**b12-^1^H NMR** (400 MHz, CDCl_3_)

**b12-^13^C NMR** (101 MHz, CDCl_3_)

**b13-^1^H NMR** (400 MHz, CDCl_3_)

**b13-^13^C NMR** (101 MHz, CDCl_3_)

**b14-^1^H NMR** (400 MHz, CDCl_3_)

**b14-^13^C NMR** (101 MHz, CDCl_3_)

**b14-^19^F NMR** (564 MHz, CDCl_3_)

**b15-^1^H NMR** (400 MHz, CDCl_3_)

**b15-^13^C NMR** (101 MHz, CDCl_3_)

**b16-^1^H NMR** (400 MHz, CDCl_3_)

**b16-^13^C NMR** (101 MHz, CDCl_3_)

**b17-^1^H NMR** (400 MHz, CDCl_3_)

**b17-^13^C NMR** (101 MHz, CDCl_3_)

**b17-^19^F NMR** (564 MHz, CDCl_3_)

**b18-^1^H NMR** (400 MHz, CDCl_3_)

**b18-^13^C NMR** (101 MHz, CDCl_3_)

**b19-^1^H NMR** (400 MHz, CDCl_3_)

**b19-^13^C NMR** (101 MHz, CDCl_3_)

**b20-^1^H NMR** (400 MHz, CDCl_3_)

**b20-^13^C NMR** (101 MHz, CDCl_3_)

**b21-^1^H NMR** (400 MHz, CDCl_3_)

**b21-^13^C NMR** (101 MHz, CDCl_3_)

**b22-^1^H NMR** (400 MHz, CDCl_3_)

**b22-^13^C NMR** (101 MHz, CDCl_3_)

**b22-^19^F NMR** (564 MHz, CDCl_3_)

**b23-^1^H NMR** (400 MHz, CDCl_3_)

**b23-^13^C NMR** (101 MHz, CDCl_3_)

**b24-^1^H NMR** (400 MHz, CDCl_3_)

**b24-^13^C NMR** (101 MHz, CDCl_3_)

**b25-^1^H NMR** (400 MHz, CDCl_3_)

**b25-^13^C NMR** (101 MHz, CDCl_3_)

**b25-^19^F NMR** (564 MHz, CDCl_3_)

**b26-^1^H NMR** (400 MHz, CDCl_3_)

**b26-^13^C NMR** (101 MHz, CDCl_3_)

**b26-^19^F NMR** (564 MHz, CDCl_3_)

**b27-^1^H NMR** (400 MHz, CDCl_3_)

**b27-^13^C NMR** (101 MHz, CDCl_3_)

**b28-^1^H NMR** (400 MHz, CDCl_3_)

**b28-^13^C NMR** (101 MHz, CDCl_3_)

**b29-^1^H NMR** (400 MHz, CDCl_3_)

Minor diastereoisomer:

**b29-^1^H NMR** (400 MHz, CDCl_3_)

**b29-^13^C NMR** (101 MHz, CDCl_3_)

**b30-^1^H NMR** (400 MHz, CDCl_3_)

Minor diastereoisomer:

**b30-^1^H NMR** (400 MHz, CDCl_3_)

**b30-^13^C NMR** (101 MHz, CDCl_3_)

**b31-^1^H NMR** (400 MHz, CDCl_3_)

Minor diastereoisomer:

**b31-^1^H NMR** (400 MHz, CDCl_3_)

**b31-^13^C NMR** (101 MHz, CDCl_3_)

**b31-^19^F NMR** (564 MHz, CDCl_3_)

**b32-^1^H NMR** (400 MHz, CDCl_3_)

Minor diastereoisomer:

**b32-^1^H NMR** (400 MHz, CDCl_3_)

**b32-^13^C NMR** (101 MHz, CDCl_3_)

**b33-^1^H NMR** (400 MHz, CDCl_3_)

Minor diastereoisomer:

**b33-^1^H NMR** (400 MHz, CDCl_3_)

**b33-^13^C NMR** (101 MHz, CDCl_3_)

**b34-^1^H NMR** (400 MHz, CDCl_3_)

Minor diastereoisomer:

**b34-^1^H NMR** (400 MHz, CDCl_3_)

**b34-^13^C NMR** (101 MHz, CDCl_3_)

**b35-^1^H NMR** (400 MHz, CDCl_3_)

Minor diastereoisomer:

**b35-^1^H NMR** (400 MHz, CDCl_3_)

**b35-^13^C NMR** (101 MHz, CDCl_3_)

**b36-^1^H NMR** (400 MHz, CDCl_3_)

**b36-^13^C NMR** (101 MHz, CDCl_3_)

**b37-^1^H NMR** (400 MHz, CDCl_3_)

Minor diastereoisomer:

**b37-^1^H NMR** (400 MHz, CDCl_3_)

**b37-^13^C NMR** (101 MHz, CDCl_3_)

**b38-^1^H NMR** (400 MHz, CDCl_3_)

Minor diastereoisomer:

**b38-^1^H NMR** (400 MHz, CDCl_3_)

**b38-^13^C NMR** (101 MHz, CDCl_3_)

**b39-^1^H NMR** (400 MHz, CDCl_3_)

**b39-^13^C NMR** (101 MHz, CDCl_3_)

**b40-^1^H NMR** (400 MHz, CDCl_3_)

Minor diastereoisomer:

**b40-^1^H NMR** (400 MHz, CDCl_3_)

**b40-^13^C NMR** (101 MHz, CDCl_3_)

**b41-^1^H NMR** (400 MHz, CDCl_3_)

**b41-^13^C NMR** (101 MHz, CDCl_3_)

**b42-^1^H NMR** (400 MHz, CDCl_3_)

**b42-^13^C NMR** (101 MHz, CDCl_3_)

**b43-^1^H NMR** (400 MHz, CDCl_3_)

**b43-^13^C NMR** (101 MHz, CDCl_3_)

**b44-^1^H NMR** (400 MHz, CDCl_3_)

**b44-^13^C NMR** (101 MHz, CDCl_3_)

**b45-^1^H NMR** (400 MHz, CDCl_3_)

**b45-^13^C NMR** (101 MHz, CDCl_3_)

**b46-^1^H NMR** (400 MHz, CDCl_3_)

**b46-^13^C NMR** (101 MHz, CDCl_3_)

**b47-^1^H NMR** (400 MHz, CDCl_3_)

Minor diastereoisomer:

**b47-^1^H NMR** (400 MHz, CDCl_3_)

**b47-^13^C NMR** (101 MHz, CDCl_3_)

**b48-^1^H NMR** (400 MHz, CDCl_3_)

Minor diastereoisomer:

**b48-^1^H NMR** (400 MHz, CDCl_3_)

**b48-^13^C NMR** (101 MHz, CDCl_3_)

**b49-^1^H NMR** (400 MHz, CDCl_3_)

Minor diastereoisomer:

**b49-^1^H NMR** (400 MHz, CDCl_3_)

**b49-^13^C NMR** (101 MHz, CDCl_3_)

## 8.3 NMR-Spectra of Transformations of the Products

**c1-^1^H NMR** (400 MHz, CDCl_3_)

**c1-^13^C NMR** (101 MHz, CDCl_3_)

**c2-^1^H NMR** (400 MHz, CDCl_3_)

**c2-^13^C NMR** (101 MHz, CDCl_3_)

**c3-^1^H NMR** (400 MHz, CDCl_3_)

**c3-^13^C NMR** (101 MHz, CDCl_3_)

**c4-^1^H NMR** (400 MHz, CDCl_3_)

**c4-^13^C NMR** (101 MHz, CDCl_3_)

## 8.4 NMR-Spectra of the Deuterated Experiments

**a10-*d_1_*-^1^H NMR** (600 MHz, CDCl_3_)

**b10-*d_1_*-^1^H NMR** (600 MHz, CDCl_3_)
